# A hybrid computational framework for intelligent inter-continent SARS-CoV-2 sub-strains characterization and prediction

**DOI:** 10.1038/s41598-021-93757-w

**Published:** 2021-07-15

**Authors:** Moses Effiong Ekpenyong, Mercy Ernest Edoho, Udoinyang Godwin Inyang, Faith-Michael Uzoka, Itemobong Samuel Ekaidem, Anietie Effiong Moses, Martins Ochubiojo Emeje, Youtchou Mirabeau Tatfeng, Ifiok James Udo, EnoAbasi Deborah Anwana, Oboso Edem Etim, Joseph Ikim Geoffery, Emmanuel Ambrose Dan

**Affiliations:** 1 Department of Computer Science, University of Uyo, P.M.B. 1017, Uyo, 520003 Nigeria; 2 Centre for Research and Development, University of Uyo, P.M.B. 1017, Uyo, 520003 Nigeria; 3 Department of Mathematics and Computing, Mount Royal University, 4825 Mt Royal Gate SW, Calgary, AB T3E 6K6 Canada; 4 College of Health Sciences, University of Uyo, P.M.B. 1017, Uyo, 520003 Nigeria; 5 National Institute for Pharmaceutical Research and Development (NIPRD), Plot 942, Cadastral Zone C16, Idu, Industrial District, Abuja, FCT Nigeria; 6 College of Health Sciences, Niger Delta University, Wilberforce Island, P.M.B. 071, Amassama, 560103 Nigeria; 7 Department of Botany and Ecological Studies, University of Uyo, P.M.B. 1017, Uyo, 520003 Nigeria; 8 Department of Biochemistry, University of Uyo, P.M.B. 1017, Uyo, 520003 Nigeria

**Keywords:** Biochemistry, Biotechnology, Computational biology and bioinformatics, Genetics, Medical research, Molecular medicine, Mathematics and computing

## Abstract

Whereas accelerated attention beclouded early stages of the coronavirus spread, knowledge of actual pathogenicity and origin of possible sub-strains remained unclear. By harvesting the Global initiative on Sharing All Influenza Data (GISAID) database (https://www.gisaid.org/), between December 2019 and January 15, 2021, a total of 8864 human SARS-CoV-2 complete genome sequences processed by gender, across 6 continents (88 countries) of the world, Antarctica exempt, were analyzed. We hypothesized that data speak for itself and can discern true and explainable patterns of the disease. Identical genome diversity and pattern correlates analysis performed using a hybrid of biotechnology and machine learning methods corroborate the emergence of inter- and intra- SARS-CoV-2 sub-strains transmission and sustain an increase in sub-strains within the various continents, with nucleotide mutations dynamically varying between individuals in close association with the virus as it adapts to its host/environment. Interestingly, some viral sub-strain patterns progressively transformed into new sub-strain clusters indicating varying amino acid, and strong nucleotide association derived from same lineage. A novel cognitive approach to knowledge mining helped the discovery of transmission routes and seamless contact tracing protocol. Our classification results were better than state-of-the-art methods, indicating a more robust system for predicting emerging or new viral sub-strain(s). The results therefore offer explanations for the growing concerns about the virus and its next wave(s). A future direction of this work is a defuzzification of confusable pattern clusters for precise intra-country SARS-CoV-2 sub-strains analytics.

## Introduction

The coronavirus disease pandemic had forced complete shutdown on all economies of the world^1,2^. Since then, its breadth and depth have grown tremendously, causing disruptions that demand a hybrid of computational approaches sufficient to discover the changing nature of the virus as it transmits from country to country. A hybrid computational approach combines more than one methodology or system for the purpose of creating new and better models. This approach is adopted in this paper to complement the efforts of biotechnology/bioinformatic solutions for intelligent mining of the SARS-CoV-2 genomes. While there exist claims that the virus has remained unchanged^3^, a growing number of studies have reported the emergence of several sub-strains^4,5^. This explains why the rapid human to human transmission of the pathogenic SARS-CoV-2 to most parts of the world has exhibited differences in disease severity and fatality even within a demographic region of a country. The disparity on the one hand has been attributed to factors such as gender, age, ethnicity, race, and co-morbidities^6^. However, the dissimilarity in genome sequencing of early viral samples obtained from infected individuals in European, North American, Asian, and Oceanian regions^7^ disgorged several studies aimed at analyzing and understanding the evolutionary history and relationships among the different SARS-CoV-2 strains.

SARS-CoV-2 is a β-coronavirus–an enveloped non-segmented positive-sense RNA virus (subgenus–sarbecovirus, subfamily–Orthocoronavirinae)^8^, which proliferation begun in December 2019 in Wuhan China. It has since been confirmed that two strains of the new coronavirus (the L- and S-strains) are spreading around the world today^9^, and the fact that the L-type is more prevalent suggests that it is “more aggressive” than the S-type. Greater proportion of research progress on SARS-CoV-2 has taken the biotechnology dimension^10,11^, specifically focusing on species characterization and variants analysis through features extraction. Consequently, Artificial Intelligence (AI) and Machine Learning (ML) methods are expanding biotechnology capacity into the bioinformatics realm, through intelligent genome probing for precise viral features classification. So far, AI/ML research on SARS-CoV-2 has permeated four key areas of medicine and healthcare, namely, screening and treatment^12–15^, contact tracing^16^, prediction and forecasting^17,18^, and drugs and vaccine discovery^19–21^.

To understand the origin and structure of SARS-CoV-2, a sequence of the viral genetic material is required. Sequencing viral genomes is performed to identify regions of similarity that may have consequences for functional, structural, or evolutionary associations^22^. Furthermore, it can reveal the possibility of future health risks and vaccine remedies. Phylogenetic tree and genomic tree (also referred to as hierarchical clustering) are common determinants for representing genetic diversity and evolutionary relationships of sequenced genomes. While phylogenetic tree reflects slow evolution within the genome (point mutations), hierarchical clustering describes major genetic re-arrangement events (insertions or deletions). Converting massive amount of complete genome sequences into meaningful biological representations has limited progress of discovering viral sub-strains and detailed transmission routes. Although numerous algorithms/tools have evolved to target specific gene sites/locations for “on-the-fly” online phylogeny representations, incomplete representation and clustering errors abound–as different genome sites undergo different evolutionary changes, resulting in disparate multi-dimensional patterns at different sites. Attempts at estimating phylogenies by comparing entire genomes have been made by focusing mainly on gene content and gene order comparisons. While early attempts concentrated on morphological characters with the premise that direct genes comparison makes more sense, modern attempts use sequences from homologous genes^22^ but are burdened by the fact that a gene’s evolutionary history may differ from the evolutionary history of the organism, as some genes sufficiently conserved across the species of interest may escape detection. Alignment-free genome comparison methods are therefore becoming popular^22,23^ and have evolved to crash the heavy computational requirements of traditional alignment-based methods. Randhawa et al.^24^ for instance proposed an alignment-free approach based on ML, for fast, inexpensive, and taxonomic classification of complete COVID-19 genomes in real time.

Variants of SARS-CoV-2 have emerged with reported new peaks of infection. A variant is a strain when it has a different characteristic. Variants with few mutations belong to the same lineage. Lineages are important for showing how a virus spreads through communities or populations. Interestingly, the less virulent strains are disappearing while those showing significant mutant variations prevail. A few documented cases of the spread of the viral sub-strains are observed based on locations, as follows: In USA, 4 sub-strains and 11 top mutations were discovered from the analysis of 12,754 complete SARS-CoV-2 genome sequences, where 2 out of 4 discovered sub-strains were potentially more infectious^25^. These sub-strains and 5 mutants were first detected in China, Singapore, and the United Kingdom^26^. In England, a sub-strain of replicative advantage was also discovered as variant of SARS-CoV-2, characterized by 9 spike protein mutations consisting of 3 deletions and 6 substitutions^27^. Some of these variants were prevalent in Netherlands, Switzerland, and France. In Southwestern Wisconsin, Southeastern Minnesota, Northeast Iowa, the sequencing of whole viral genomes of COVID-19 positive patients showed the spread of sub-strains to individuals in 13 cities from epicenters of the infection^28^. However, no viral sub-strain was observed in China^5^.

Vaccine types are also being circulated with several conspiracy theories and disbeliefs about the virus existence spreading across the globe. There is fear that emerging sub-strain variants may confer resistance to antibody neutralization, as evolving variants of concern are rapidly growing lineage to SARS-CoV-2 with high replicable mutants that may hinder the efficiency of existing vaccines and expand in response to the increasing after‐infection or vaccine‐induced seroprevalence^27^. Currently, most COVID-19 vaccines target the viral spike protein. Although mutations may reduce their efficacy, they do not obliterate their effects. Inactivated virus vaccines that target the whole virus have been developed in China, as the immune responses they induce target more than a single part of the spike protein; hence, inducing several protective immune responses and instilling redundancy in the protective immune responses.

Mining additional knowledge from clinical data would assist complete features extraction, missing information recovery, hidden patterns understanding, and facilitate output targets labeling. Most biotechnology/bioinformatics tools are ‘black boxes’ and not open to contributions from the research community including reproducible research. Furthermore, extracted features are incomplete to aid meaningful knowledge integration. To support the growing field of medical- and bio- informatics, this paper adopted a novel approach to genome sequence mining. Transitions in nucleotide (dinucleotide) and changes in gene (mutation) information were exploited as input features or predictors, as these features have direct connection with the behavior of the virus. A hierarchical agglomerative clustering method was applied on the extracted features to detect optimal natural clusters for determining the evolutionary group of the various isolates, across countries. Using a self-organizing map (SOM), genome patterns with low similarity profile (or highly variable genomes) including the reference genome, were discerned to visually establish which sub-strain group(s) the various genome samples or isolates belong. By decoupling the SOM map through correlation hunting, a cognitive map that associates similar isolate clusters was obtained. The generated patterns and isolate similarity information provided details for enriching the input dataset through a supervised labelling of the classification targets. Statistical analysis validated the variability of the SARS-CoV-2 isolates. This research has therefore made substantial contributions to knowledge, as it provides the following:

(i) *Useful Intermediate Results* As opposed to most biotechnology and bioinformatic tools, useful intermediate results are produced in this paper to give further insights into the prevalence and transmission of SARS-CoV-2. The research is also replicable, as the algorithms and data are available to reproduce and validate our results.
(ii) *Support for the Contact Tracing of Undocumented Source of Infection* Tracing infectious diseases routes for efficient documentation of infected cases is very crucial in emerging pandemic situations. While the excavated data holds only few traces of transmission history, our pattern clustering and cognitive knowledge mining results groups the various isolates into sub-strain clusters. This information is then used to label the output targets for classification and prediction, hence, providing understanding of which of the viral sub-strain(s) maintain(s) the reference genome pattern or is/are spreading within a particular country or been acquired from a different country. Furthermore, pattern progressions indicating emerging cluster transitions are revealed by the self-organizing map deployed in this study.
(iii) *Intelligent System Framework* From labelled classification targets, accurate sub-strain classification and prediction is achieved. The proposed framework combines machine learning techniques and cognitive knowledge mining to extract dinucleotide and mutation frequencies for base variant analysis. Also, hidden sub-strains interactions between nucleotide sequences and other information not hitherto seen in the raw data are uncovered.
(iv) *Gender-Specific Isolates Mining *To engage meaningful research in SARS-CoV-2, characterization and prediction by gender is crucial. This aspect which is often missing in the literature was excavated from GSAID. A metadata of excavated SARS-CoV-2 genomes by gender is available (Data S7: SupplData7.xlsx). The metadata permits the intelligent mining of SARS-CoV-2 demographic information, as ambiguities in annotation labels inherent in the Global initiative on Sharing All Influenza Data (GISAID) database (https://www.gisaid.org/) have been resolved in this paper. Input features and classification target labels of unique isolates based on SOM cluster analysis and cognitive knowledge mining are also available (Data S8: SupplData8.xlsx). These resources can be integrated into expert decision-making systems to support early contact tracing and global disease surveillance.

## Related works

Several studies have dwelled on the characterization of SARS-CoV-2 genome for tracing the evolution, strains, and diversity of the virus. In Tang et al.^9^, for instance, a population genetic analysis of 103 SARS-CoV-2 genomes was performed. Their analysis revealed two dominant types of SARS-CoV-2 namely the L type (~ 70%) and S type (~ 30%). In another study, Stefanelli et al.^7^ investigated the phylogeny of 2 patients in Italy; a Chinese tourist from Wuhan and an Italian diagnosed, isolated, and hospitalized in January and February 2020. They found the Italian patient’s strain to be different from the tourist’s strain, as it clustered with strains from Germany and Mexico, while the Chinese tourist’s strain was grouped with strains from Europe and Australia. Similarly, Somasundaram et al.^29^ systematically explored the phylogenetic and viral clade of 28 Indian isolates of SARS-CoV-2. A total of 449 complete genome samples from USA, Europe, China, East Asia, Oceania, Middle East (Kuwait and Saudi Arabia) and India were collected from GISAID. A phylogenetic analysis by maximum likelihood was achieved using IQ tree. Out of the Indian isolates, 26 samples were equally distributed into 2 clusters (A and B). Cluster A consisted of mostly Oceania/Kuwait and 13 Indian samples, while cluster B contained Europe and some of Middle East/South Asian samples together with another 13 Indian samples. The remaining 2 Indian isolates which neither belonged to cluster A nor cluster B, were present in the cluster with mostly China and East-Asia samples. However, the use of small datasets and the lack of travel history rendered their findings inconclusive.

Application of ML in the combat of COVID-19 has inspired new discoveries as well as improved methods based on experience of previous/related epidemic. Familiar areas of application center around medical imaging, disease tracing, epidemiology modeling and medicine (analysis of protein structure and drug discovery) and virulent nature of the virus. Whereas the processing of input data for informed decision support is necessary, the types of data exploited in the case of SARS-CoV-2 and related pandemic are mainly demographic and/or (control or clinical data) contributed by patients/volunteers around the world. Table 1 presents a summary of works carried out on ML/AI in related areas of application, indicating the objective, number of isolates collected and data source, methods, results/findings, and drawbacks. From the related works, we observe the following: (i) Most of the works explore hybrid tools that combine biotechnology and ML/AI methodologies, which have advanced precision in approach and solution to the pandemic. (ii) While 50% of the works rely on limited genomic evidence, others are mainly simulation studies. (iii) The fulcrum of most of the works revolve around characterization and forecasting with comparative analysis of SARS-CoV-2 evolution, and relationship between it and (other) related viruses. (iv) All the works are silent on the gender dimension. (v) None of these works to the best of our knowledge has engaged the possibility of SARS-CoV-2 sub-strains discovery.

**Table 1:** Summary of ML/AI application of SARS-CoV-2 characterization and prediction.

Reference	Objective	Number of isolate and source	Method	Result/finding	Drawback
Randhawa et al.^21^	To combine machine learning-based alignment-free approach with COVID-19 genomic signature for real-time taxonomic predictions of unclassified new sequences of COVID-19	5538 unique viral genome sequences, totaling 61.8 million bp, including 29 COVID-19 virus sequences available on January 27, 2020. Sequence data came from NCBI, Virus- Host-DB, and GISAID	Combined supervised machine learning with digital signal processing (MLDSP), augmented by decision tree, for genome analysis. Spearman’s rank correlation was then used for result validation	Results support the bat origin and classified the COVID-19 virus as Sarbecovirus, within Betacoronavirus Their method achieved high classification accuracy for the COVID-19 virus sequences; and can provide a reliable real-time option for taxonomic classification	Study only compared the relatedness of the COVID-19 virus sequences to the known genera of Coronaviridae family and known sub-genera of the genus Betacoronavirus
Khanday et al.^30^	To classify textual clinical reports on SARS-related viruses using classical and ensemble machine learning algorithms	212 patients’ data showing symptoms of coronavirus and other viruses were collected from GitHub^31^	Feature engineering was performed using Term frequency/inverse document frequency (TF/IDF), Bag of words (BOW) and report length. These features were then learned using traditional and ensemble machine learning classifiers that classified the text into four different categories: COVID, SARS, ARDS and Both (COVID, ARDS)	Logistic regression and Multinomial Naive Bayes performed better than other ML algorithms	Study relied on limited amount of data
Melin et al.^32^	To analysis the spatial evolution of coronavirus pandemic around the world	Publicly available datasets were obtained from the Humanitarian Data Exchange (HDX)^33^, from countries where COVID-19 cases had occurred between January 22, 2020 and May 13, 2020	The proposed method used the Kohonen self-organizing maps to form clusters of countries in the world. The classification was achieved using 4 classes of COVID-19 severity cases (Very High, High, Medium, and Low)	Interesting conclusions that may be helpful in deciding the best strategies in dealing with the virus were drawn from extensive simulation	The research was mainly a simulation study
Melin et al.^34^	To develop a multiple ensemble neural network model with fuzzy response aggregation for the COVID‐19 time series	Dataset from confirmed COVID‐19 cases and death cases, which consists of 12 states in Mexico and the total data of the country	A 3-module ensemble architecture was deployed, with each ensemble having its own fuzzy aggregator, for final prediction of the ensemble	The proposed multiple ensemble neural network models with fuzzy response integration closely followed real data and yielded precise predictions in the validation dataset	The research was mainly a simulation study
Castillo and Melin^35^	To forecast confirmed COVID-19 cases and death based on the complexity of their time series using a hybrid fuzzy-fractal approach	Publicly available datasets of 10 countries were obtained from the Humanitarian Data Exchange (HDX) and data from countries where COVID-19 cases have occurred from January 22, 2020 to March 31, 2020	The datasets were used to build the fuzzy model with time series in a fixed period. Then the fuzzy fractal model was tested by forecasting other times series in window periods of 10 days	Simulated forecast results were close to the real values, confirming that the fuzzy fractal approach works well in time series prediction	The research was mainly a simulation study and limited to COVID-19 cases
Lopez-Rincon et al.^36^	Deep learning is coupled with explainable artificial intelligence techniques to discover representative genomic sequences in SARS-CoV-2	10,712 SARS-CoV-2 sequences were excavated on December 23, 2020 from The Global Initiative on Sharing All Influenza Data (GISAID) repository	Convolutional neural network classifier was first trained on 553 sequences, separating the genome of different virus strains from the Coronavirus family. The network’s behavior was then analyzed, to discover sequences used to model SARS-CoV-2 identification. The sequences were later validated on the excavated samples	12 meaningful 21-bps sequences that best characterized SARS-CoV-2 were discovered. For all the analyzed data, these sequences appeared only in SARS-CoV-2 samples and not in any other viruses	The study concentrated on specific genome sites
Lopez-Rincon et al.^37^	To propose an assisted detection test that combines molecular testing with deep learning	Dataset of 553 complete genome non-repeated sequences that vary from 1260 to 31,029 bps in length was collected from 2019 Novel Coronavirus Resource (2019nCoVR) repository^38^	Deep convolutional neural network using tenfold classification was deployed for automatic features creation starting from the genome sequence of the virus	The proposed approach could correctly classify SARS-CoV-2, distinguishing it from other coronavirus strains, regardless of missing information and errors in sequencing (noise)	Their work concentrated on specific genome sites
Kaden et al.^39^	To investigate SARS-CoV-2 virus sequences based on alignment-free methods for RNA sequence comparison	Viral sequence genomes from GISAID–with 156 genomes, and NCBI and GenBank–with 892 complete genomes, by April 19, 2020, were excavated	A Generalized Matrix Learning Vector Quantizer (GMLVQ) model for labeled dataset with virus type information, obtained by phylogenetic tree analysis, was performed using tenfold cross validation. From classification correlation matrix delivered by GMLVQ optimization, features contributing decisively to a typed separation were determined	The GMLVQ approach produced lower complexity and allowed easy out-of-training generalization	Rejected sequences could only allow speculations about new virus types with respect to nucleotide base mutations in the viral sequences
Sawmya et al.^40^	To track SARS-CoV-2 evolution among countries using phylogenetic analysis and perform deep learning classification for identification of virulent strains	10,179 sequences from 67 countries were excavated from GISAID as of April 24, 2020	ML and Deep learning models were used to identify the virulence of the strains. From the classification pipeline, important features were identified as sites of interest (SOI) in the virus strains for further analysis	As regards virulent strain prediction, LightGBM classifier was superior to deep learning classifiers. As regards mutation prediction, CNN-LSTM and CNN-bidirectional LSTM gave near similar performance for different SoI of the genome	Their work was unable to explain some strong relationships between countries, as inferred by the phylogenetic tree
Sun and Wang^41^	To develop mathematical model for characterizing imported and asymptomatic patients	Study relied on demographic data on COVID-19 epidemic in Heilongjiang province from January 23 to March 25, 2020	An ordinary differential equation model was trained to fit the epidemic data and the simulation extended to characterize an infected/imported case as well as asymptomatic patients	Imported case was responsible for the newly confirmed COVID-19 infections in the province. Stochastic simulations showed significant increase in local contacts and outbreak of COVID-19. Reported number of asymptomatic patients was markedly lower than the model predictions, implying large unidentified asymptomatic pool	The research was mainly a simulation study and limited to COVID-19 cases
Dey and Mukhopadhyay^42^	To build machine learning models that predict protein–protein interactions (PPIs) between the virus and human proteins	SARS-CoV-2 human PPI database^43^ containing 332 unique interactions between 332 human proteins and four structural and as well as 20 accessory coronavirus proteins	Classification models were prepared based on different sequence-based features of human proteins like amino acid composition, pseudo amino acid composition, and conjoint triad	The ensemble voting classifier using SVM^Radial^, SVM^Polynomial^, and Random Forest technique, gave greater accuracy, precision, specificity, recall, and F1 score compared to other models	Their classifier yielded 70% accuracy due to limited experimental data
Dlamini et al.^44^	To analyze intrinsic dinucleotide genomic signatures for whole genome sequence data of 8 pathogenic species, including SARS-CoV-2	About 33,000 Fully assembled, whole genome sequence in fast-all (FASTA) format were retrieved from GISAID, for 8 pathogenic species	The genome sequences were transformed into dinucleotide relative frequencies and classified using extreme gradient boosting (XGBoost) model	Their result was able to discriminate between distantly related species such as viruses and bacteria, closely related species such as SARS-CoV-2 and MERS-CoV, as well as samples of the same species that originate from different regions	Classes with small sample size (e.g., Africa), yielded high misclassification rate

The abundance of repetitive DNA in human genome assembly has introduced huge gap of multi-megabase heterochromatic regions that challenges standard mapping and assembly algorithms. Consequently, the composition of the sequence and potential functions of these regions have largely remained unexplored. Furthermore, existing genome tools cannot readily engage complete genome analysis to predict complex details and reveal hidden patterns, essential to offer explanations to the increased diversity of viral diseases. This work is therefore motivated by the existing gap between scientific knowledge and clinical application. Despite current advancement in state-of-the-art predictions, application of personalized genomics into clinical practice is yet to flourish. By identifying relevant genetic variants using experiential knowledge we provide inference of the genetic impact of the variants on functional genomic elements.

## Results and discussion

**Figure 1:**
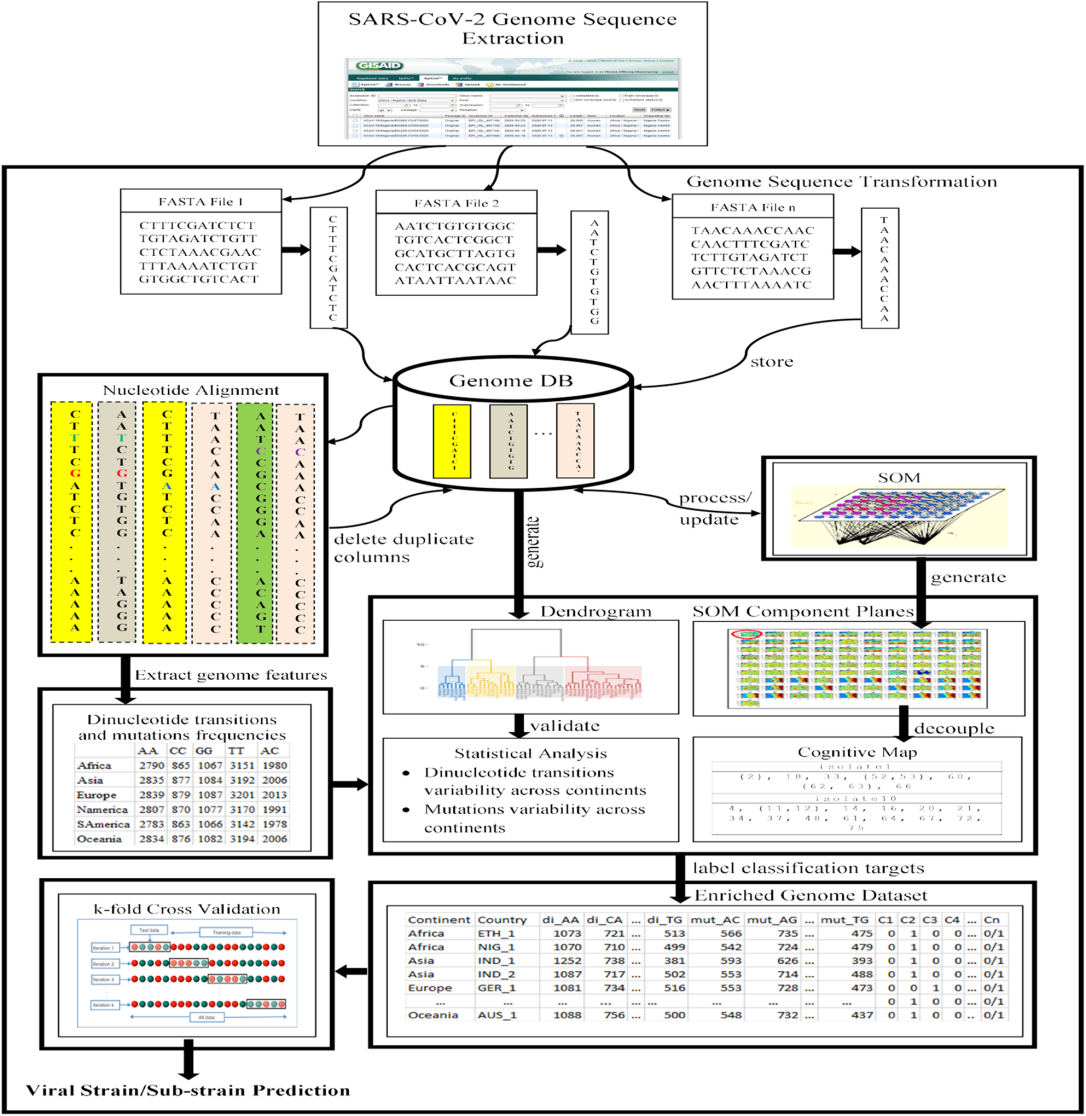
Workflow describing the proposed hybrid approach. The workflow begins with the excavation of FASTA files of human SARS-CoV-2 genome sequences from GISAID. These files were stripped and processed into a genome database (DB) as multiple columns of nucleotide sequence. AI/ML techniques were then applied to extract knowledge from the genome datasets as follows: Using ML techniques, compute dis(similarities) scores between the various pairs of genome sequences and obtain a genomic tree of highly dis(similar) isolates grouped in the form of a dendrogram/phylogenetic tree. Determine the optimal number of natural clusters—to provide additional knowledge for supervised learning. Separate the viral sub-strains using SOM component planes—for possible transmission pathway/pattern visualization. Perform nucleotide alignment of the entire genome sequences (owing to varying sequence lengths of the different genome isolates, a cutoff at the last nucleotide of the genome isolates or the reference genome serves as the maximum pair for comparison), remove duplicate columns while imposing a similarity threshold–to yield unique genome sequences. Extract genome features by computing dinucleotide transitions and mutation frequencies. Generate cognitive map–for intelligent sub-strains prediction. Label classification targets of extracted features using derived SOM clusters and cognitive map. Learn and predict new/emerging sub-strains using ANN with k-fold validation method.

The general workflow describing the proposed hybrid computational framework is presented in Fig. 1, and the sequence of steps implementing the workflow is given on Supplementary Table S1. In addition to describing the steps, a visual demonstration of the implementation is incorporated.

### Base variant analysis

Dinucleotide transitions and nucleotide mutations were computed for male and female isolates and averaged across the various continents namely Africa (Data S1: SupplData1.xlsx), Asia (Data S2: SupplData2.xlsx), Europe (Data S3: SupplData3.xlsx), North America (Data S4: SupplData4.xlsx), South America (Data S5: SupplData5.xlsx), and Oceania (Data S6: SupplData6.xslx). We analyze the average base transitions and mutations, and how they influence the overall behavior of the datasets.

#### Dinucleotide transitions

**Figure 2:**
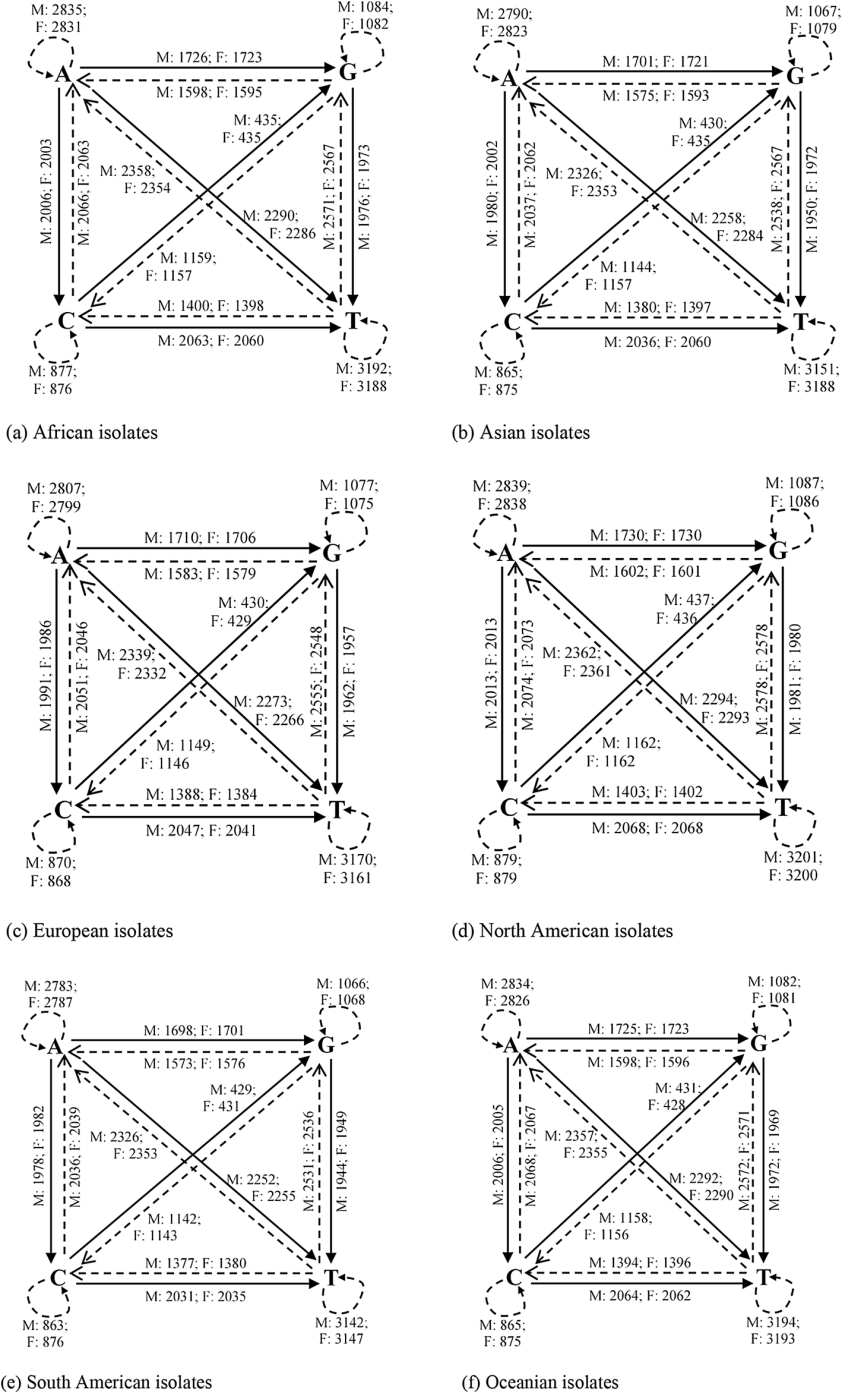
Base pair transitions in SARS-CoV-2 genomes for male and female isolates. Thick arrows indicate transition, while dotted arrows represent transversion. Looped (dotted) arrows represent same base transition. Inscriptions on/near the arrows represent transition/transversion frequencies for male and female isolates.

Averages of dinucleotide transitions of SARS-CoV-2 genomes computed across the various continents are presented in Fig. 2. These transitions are represented as quadrilaterals dissected along its diagonals. Wang et al.^45^ found that the SARS-CoV-2 reference genome has 29.94% of A, 32.08% of T, 19.61% of G and 18.37% of C. Hence, the expected dinucleotide transitions proportion is the product of the two nucleotide bases. For instance, the CG dinucleotide in the viral genome is 3.60% (i.e., 19.61% × 18.37%). From this, we arrive at the following computations for the respective dinucleotides/features identified in this study: AA = 8.96%; CC = 3.37%; GG = 3.84%; TT = 10.29%; AC = 5.50%; AG = 5.87%; AT = 9.60%; CG = 3.60%; CT = 5.87%; GT = 6.29%; TG = 6.29%; TC = 5.87%; TA = 9.60%; GC = 3.60%; GA = 5.87%; and CA = 5.50%. Our results corroborate Wang et al.^45^ on CG dinucleotide reduction of SARS-CoV-2, as the CG transitions for both male (M) and female (F) isolates across the various continents present lowest dinucleotide transitions compared to the rest of the transitions. Furthermore, slightly different variations exist between male and female transitions, which may not be unconnected with genome sequencing errors and the presence of new viral sub-strain(s).

#### Average dinucleotide transitions variant

Observed transitions variants between male and female isolates (M–F) computed from Fig. 2, across the various continents are shown in Table 2. Positive values indicate male frequency dominance while negative values indicate female frequency dominance. Table 2 reveals that female isolates from Africa greatly dominated the dinucleotide transitions space compared to male isolates, a possible pointer to sequencing errors observed in the raw genomes. Other continents however show negligible variations.

**Table 2:** Observed average dinucleotide transitions variants between male and female isolates.

Continent	AA	CC	GG	TT	AC	AG	AT	CG	CT	GT	TG	TC	TA	GC	GA	CA
Africa	− 32	− 10	− 12	− 38	− 23	− 20	− 26	− 5	− 24	− 23	− 29	− 17	− 27	− 13	− 18	− 24
Asia	4	1	2	4	2	3	3	0	3	3	4	2	4	2	2	2
Europe	2	0	0	1	1	1	1	0	1	1	1	1	1	0	0	1
North America	9	2	2	10	5	5	7	1	6	5	6	4	7	3	5	5
South America	− 3	− 2	− 2	− 5	− 4	− 3	− 3	− 1	− 4	− 5	− 5	− 3	− 4	− 2	− 3	− 3
Oceania	7	1	2	1	1	2	2	3	2	3	1	4	2	2	1	1

#### Nucleotide mutations

**Figure 3:**
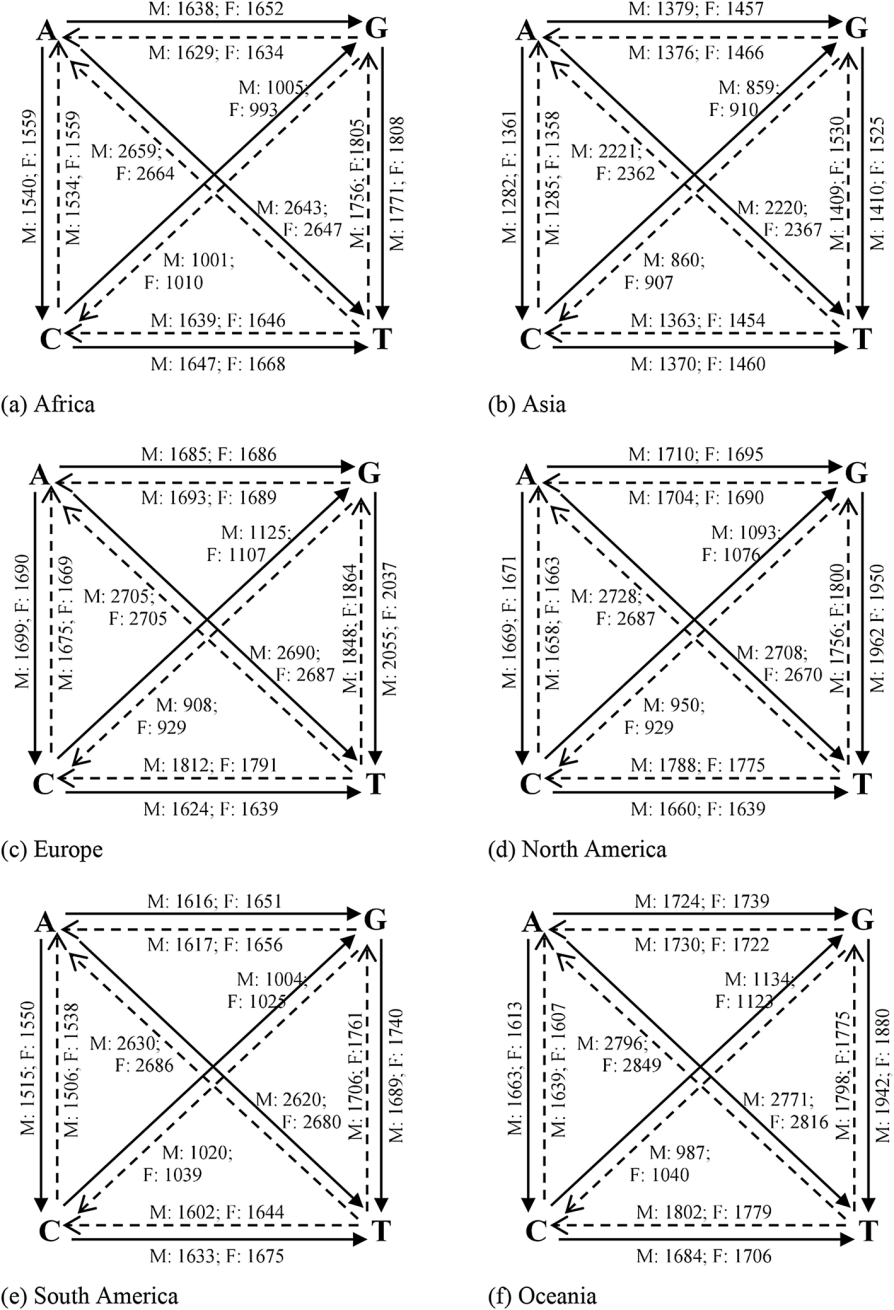
Base pair changes in SARS-CoV-2 genomes for male and female isolates. Thick arrows indicate transitions, while dotted arrows represent transversions. Inscriptions on/near the arrows represent transition frequencies for male and female isolates.

Mutations in base pairs are important for understanding the pathogenicity of SARS-CoV-2. These computations were compiled after direct pairwise comparisons with the reference genome, averaged across the various continents, to produce Fig. 3. As expected, changes in base pairs were observed after pairwise comparisons. Also, genome sequences with very negligeable changes or (no significant mutations) from the reference genome were noticed across the various continents for male and female isolates (see Table 3). Overall, total insignificant mutants of 587, representing 14.98% of the total number of isolates was observed for male patients, while female patients showed 258 insignificant mutants, representing 9.06% of the total number of isolates.

**Table 3:** Isolates with insignificant mutants across continents.

Continent	Country	Male			Female		
		No. of insignificant mutants	Total isolates	%	No. of insignificant mutants	Total isolates	%
Africa	South Africa	27	503	5.37	56	1004	5.58
	Tunisia	4	19	2.11	0	0	–
Asia	Singapore	10	487	2.05	6	53	1.13
	China	47	189	24.86	47	131	35.88
	Sri Lanka	2	23	8.70	0	0	–
	Bangladesh	8	22	36.36	0	0	–
	India	5	1041	0.48	2	557	0.36
	Kazakstan	5	14	35.71	9	10	90
	Indonesia	10	64	15.63	4	27	14.81
	Turkey	2	80	2.50	0	0	–
	Taiwan	24	34	70.59	20	30	66.67
	Philippines	1	6	16.67	0	0	–
	Israel	–	–	–	1	15	6.67
	Saudi Arabia	384	408	94.12	77	91	86.83
	Oman	1	81	1.23	0	0	–
	United Arab Emirates	22	73	30.14	9	38	23.68
Europe	Romania	1	18	5.56	0	0	–
	Spain	3	148	2.03	4	117	3.42
	Italy	6	309	1.94	5	253	1.98
	Russia	1	42	2.38	2	83	2.41
	France	1	78	1.28	1	53	1.89
North America	Mexico	2	66	3.30	2	44	4.55
	Dominican Republic	–	–	–	1	5	0.20
South America	Chile	1	1	100	0	0	–
	Colombia	3	133	2.26	2	77	2.60
	Ecuador	4	21	19.07	0	0	–
	Brazil	13	58	22.41	10	261	3.83
Total		587	3918	14.98	258	2849	9.06

#### Average nucleotide mutations variant

In an analysis of SARS-CoV-2 mutations in the United States, CT mutant variants were found to have strong gender dependence^22^. Observed mutation variants between male and female isolates (M–F) computed from Fig. 3, across the various continents are shown in Table 4. Positive values indicate male frequency dominance while negative values indicate female frequency dominance. Table 4 reveals that female isolates from Asia greatly dominate the nucleotide mutations compared to male isolates. This trend is consistently followed by female isolates from South America with dominant transitions compared to male isolates. However, other mutation statistics have mixed dominant values with varying degree of dominance. The result indicates that nucleotide mutations (not only the CT mutant) dynamically vary between individuals and are more associated with the virus adaptability to its host or environment.

**Table 4:** Observed mutant variants between male and female isolates.

Continent	AC	AG	AT	CG	CT	GT	TG	TC	TA	GC	GA	CA
Africa	− 19	− 14	− 3	12	− 21	− 37	− 48	− 8	− 5	− 8	− 5	− 26
Asia	− 79	− 78	− 147	− 52	− 91	− 115	− 121	− 91	− 141	− 47	− 90	− 73
Europe	9	− 1	3	18	− 15	18	− 16	21	0	− 21	4	6
North America	− 2	15	38	17	21	12	14	13	41	21	14	− 4
South America	− 35	− 35	− 59	− 20	− 42	− 51	− 55	− 41	− 56	− 19	− 39	− 32
Oceania	50	− 15	− 45	11	− 23	62	23	23	− 53	− 54	9	32

### Hierarchical clustering analysis (agglomerative nesting: AGNES)

Li et al.^46^ investigated the angiotensin-converting enzyme 2 (ACE2)—the receptor agent for the SARS-CoV-2 virus—a known contributor to viral infections susceptibility and/or resistance^47^. ACE2 generates small proteins by cutting up larger protein angiotensinogen, in turn affecting the nucleotide/protein. They compared ACE2 expression levels across 31 normal human tissues between males and females and between younger and older persons using two-sided student’s t-test. By examining the expression patterns, they found that protein expression levels were similarly expressed between males and females or between younger and older persons in experimented tissues. Furthermore, men showed worse prognosis than women. Their findings however lacked experimental and clinical data validation.

Using clinical evidence, we provide results of hierarchical clustering analysis to examine the arrangement of the nucleotide (protein) sequences/clusters across the entire genome through mutant accumulation, for male and female patients. Three distance measures were experimented, the ward, complete and single methods. The ward method had the highest agglomerative coefficient of (male = 0.9746; female = 0.9683), indicating more compact clusters; closely followed by complete (male = 0.9579; female = 0.9523); average (male = 0.9423; female = 0.9445); and single (male = 0.8710; female = 0.9058) methods.

To determine if differences exist in the genome sequences between genders, an independent t-test was imposed on the AGNES dis(similarity) scores. Results showed that male patients had statistically insignificantly longer genome sequences (0.9726 ± 0.0377) compared to female patients (0.9673 ±  0.0344), $t\left(3280\right)=1.710$, $p=0.0871$. However, there was no statistically significant difference in mean similarity between the nucleotide (protein) structures of the two groups at 95% confidence interval, hence, no significant genetic variations were observed. This result therefore corroborates the findings in Li et al.^43^ and validates the claim that no significant genetic variation exists in human SARS-CoV-2 genomes for both groups.

### Genome pattern analysis

Component planes reveal the distribution of single feature values on a SOM map. They permit an investigation of continents that share similar variant(s) or sub-strain(s) of SARS-CoV-2 and which variant permeates the different regions. Each component plane expresses the genome pattern of an isolate, where similar nucleotides are placed closely together in a 2D space. Hence, the patterns are established based on accumulation of nucleotides rather than individual nucleotide changes. To account for the variability in SOM neighborhood structure at every SOM run, the reference genome was included as part of the experiment datasets during each training phase. Hence, 4 reference genome pattern possibilities were generated to establish the very topology suitable for the trained datasets.

**Figure 4:**
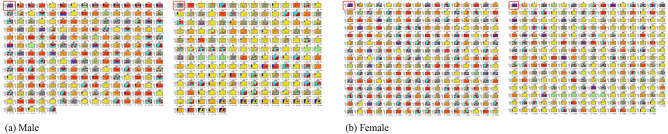
SOM component planes visualization for African isolates. Component planes 1 (encircled) represent the SARS-CoV-2 reference genome. The male and female isolates have 2 SOM maps each with country and (component plane map position(s)) distributed as follows: Male—(**a**) Map 1: Cameroon (2), Ghana (3–15), South Africa (16–200). Map 2: South Africa (2–63), Gambia (64–66), Algeria (67), Egypt (68–81), Tunisia (82–90), Morocco (91–92), Mozambique (93–96), Nigeria (97–107), Senegal (108–156), Rwanda (157–173). Female—(**b**) Map 1: Ghana (2), South Africa (3–240). Map 2: South Africa (2–186), Gambia (187), Algeria (188), Egypt (189–194), Tunisia (195–203), Madagascar (204), Nigeria (205–208), Senegal (209–237), Rwanda (238–239).

**Figure 5:**
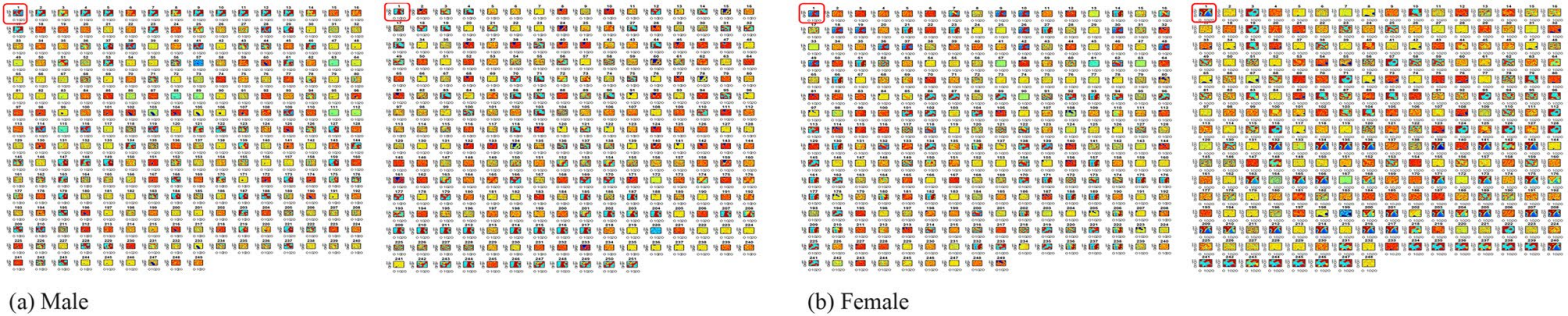
SOM component planes visualization for Asian isolates. Component planes 1 (encircled) represent the SARS-CoV-2 reference genome. The male and female isolates have 2 SOM maps each with country and (component plane map position(s)) distributed as follows: Male—(**a**) Map 1: Singapore (2–18), Iraq (19), China (20–71), Kuwait (72–74), Malaysia (75–94), Sri Lanka (95–109), Bangladesh (110–119), India (120–249). Map 2: India (1–145), South Korea (146–149), Kazakhstan (150), Indonesia (151–164), Turkey (165–180), Iran (181–184), Taiwan (185–191), Vietnam (192–200), Israel (201), Saudi Arabia (202–221), Mongolia (222–224), Oman (225–231), Lebanon (232–240), United Arab Emirates (241–251). Female—(**b**) Map 1: Singapore (205), Iraq (6), China (7–54), Malaysia (55–79), Sri Lanka (80–85), Bangladesh (86–90), India (91–249). Map 2: India (2–129), South Korea (130–131), Kazakhstan (132–136), Indonesia (137–149), Turkey (150–159), Iran (160–162), Taiwan (163–176), Vietnam (177–193), Israel (194–197), Philippines (198–199), Saudi Arabia (200–217), Pakistan (218–219), Oman (220–227), Lebanon (228–233), United Arab Emirates (234–247), Bahrain (248).

**Figure 6:**
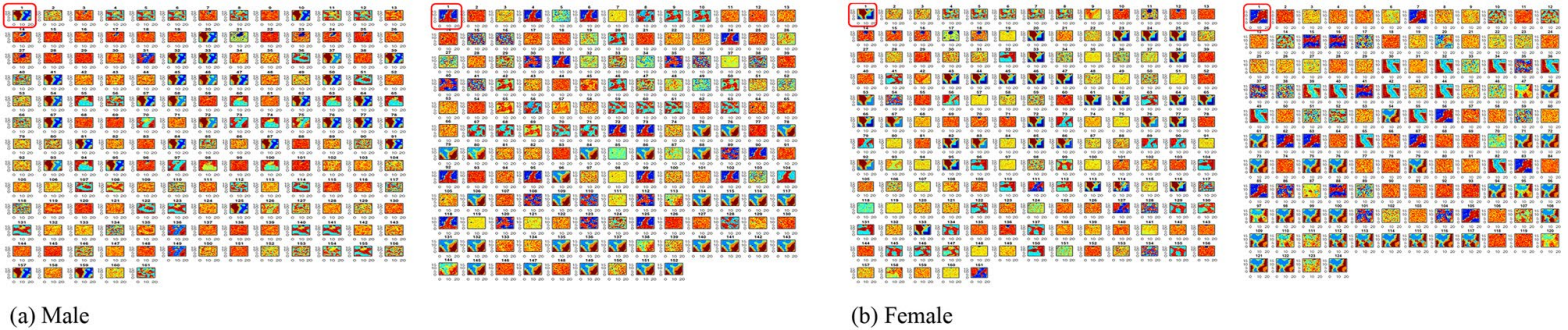
SOM component planes visualization for European isolates. Component planes 1 (encircled) represent the SARS-CoV-2 reference genome. The male and female isolates have 2 SOM maps each with country and (component plane map position(s)) distributed as follows: Male—(**a**) Map 1: Switzerland (2), Faroe Island (3–7), Belgium (8–9), Poland (10–23), Greece (14–29), Romania (30–43), Spain (44–102), Georgia (103–105), Italy (106–161). Map 2: Italy (2–59), Russia (60–73), France (74–112), Slovakia (113), Hungary (114–118), Cyprus (119), Ukraine (120–125), Sweden (126), Austria (127), Croatia (128–129), Bosnia and Herzegovina (130), Czech Republic (131–152). Female—(**b**) Map 1: Switzerland (2), Faroe Islands (3–6), Belgium (7–8), Greece (9–19), Germany (20–26), Romania (27–47), Spain (48–95), Georgia (96), Italy (97–161). Map 2: Italy (2–28), Russia (29–55). France (56–87), Slovakia (88–90), Moldovia (91–93), Hungary (94–100), Ukraine (101–104), Austria (105), Finland (106), Bosnia and Herzegovina (107), Czech Republic (107–123).

**Figure 7:**
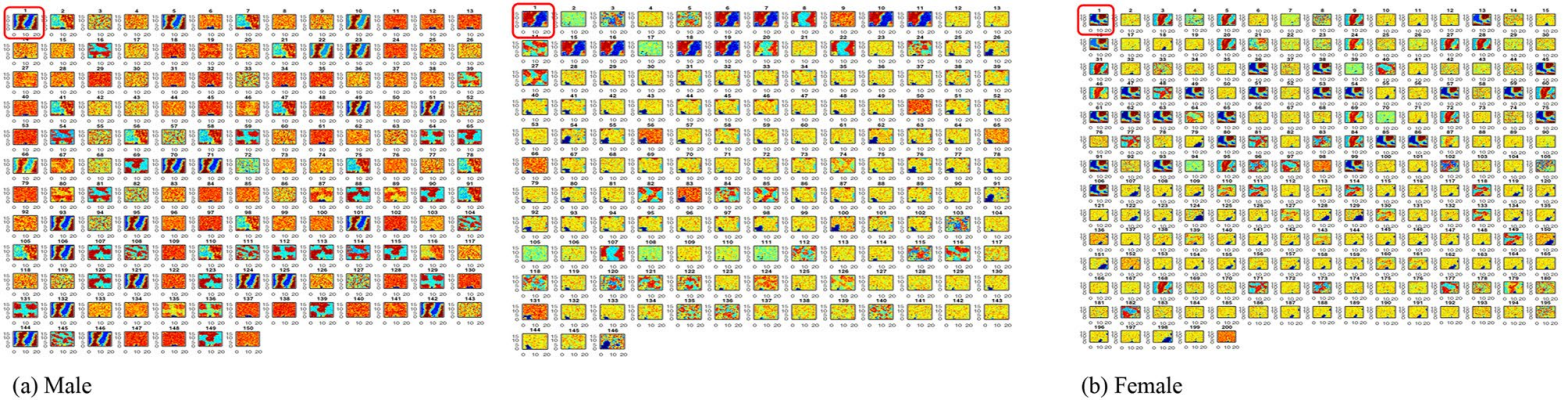
SOM component planes visualization for North American isolates. Component planes 1 (encircled) represent the SARS-CoV-2 reference genome. The male isolates have 2 SOM maps while the female isolates have 1 map, each with country and (component plane map position(s)) distributed as follows: Male—(**a**) Map 1: Mexico (2–46), USA (47–150). Map 2: USA (2–23), Panama (25–102), Saint Martin (103–105), Guadeloupe (106–109), Canada (110–112), Costa Rica (113–145), Dominican Republic (146). Female—(**b**) Map 1: Mexico (2–34), USA (35–106), Panama (107–165), Saint Martin (166–168), Guadeloupe (169–176), Canada (177–182), Costa Rica (183–196), Dominican Republic (197–200).

**Figure 8:**
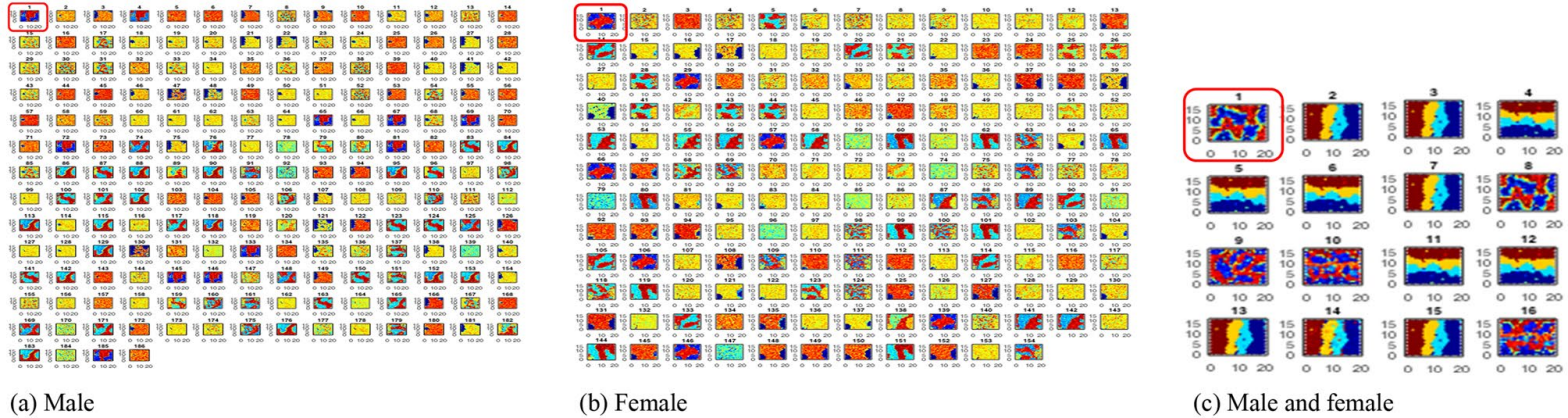
SOM component planes visualization for South American and Oceanian isolates. Component planes 1 (encircled) represent the SARS-CoV-2 reference genome. For South American isolates, the male isolates (**a**) and female isolates (**b**) have 1 SOM map each. For Oceanian isolates, the male and female isolates (**c**) are condensed into 1 map, each with country and (component plane map position(s)) distributed as follows: Male—(**a**) Map 1: Venezuela (2–3), Chile (4), Argentina (5), Colombia (6–62), Ecuador (63–72), Peru (73), Brazil (74–186).

Our topologies possess random (but controllable) discontinuities that permit more flexible self-organization with high-dimensional data, thus, preserving as much as possible, the map structure. The SOM training was carried out by gender, per continent. To ensure clear visualization of the generated maps, most of the gender-specific runs were split into 2 runs. This method was adopted to reduce the computational burden accompanying the huge datasets in this study. A total of 18 SOM maps were generated (see Figs. 4, 5, 6, 7, 8). We observed single-, double- and multiple-source transmissions. Overall, 7 pattern clusters were discovered as documented in Table 5. Cluster 1 represents the reference genome. Clusters 2, 3, 4, 5 and 6 are inter-continent pattern clusters or sub-strain(s). Cluster 7 indicates discovered intra-country pattern clusters or sub-strains. The analysis of Wang’s et al.^22^ suggests the presence of four sub-strains in the United States. Our results therefore sustain an increase in sub-strains within the various continents and offer explanations for the growing concerns and next wave(s) of the virus.

**Table 5:** Cluster distribution by gender across continents.

Continent	Gender	Cluster 1 (reference genome)		Cluster 2 (inter-country sub-strain G1)		Cluster 3 (inter-country sub-strain G2)		Cluster 4 (inter-country sub-strain G3)		Cluster 5 (inter-country sub-strain G4)		Cluster 6 (inter country sub-strain G5)		Cluster 7 (intra-country sub-strain)		Total
		No	%	No	%	No	%	No	%	No	%	No	%	No	%	%
Africa	Male	14	3.77	75	20.22	86	23.18	10	2.70	0	0	25	6.74	161	43.40	100
	Female	17	3.56	111	23.27	99	20.75	10	2.10	0	0	64	13.42	176	36.90	100
Asia	Male	26	5.22	136	27.31	87	17.47	8	1.61	2	0.40	82	16.47	157	31.53	100
	Female	40	8.08	119	24.04	81	16.36	19	3.84	1	0.20	68	13.74	167	33.74	100
Europe	Male	41	13.18	108	34.73	13	4.18	8	2.57	2	0.64	20	6.43	119	38.26	100
	Female	39	13.78	77	27.21	32	11.31	9	3.18	10	3.53	8	2.83	108	38.16	100
North America	Male	27	9.18	74	25.17	103	35.03	7	2.38	0	0	18	6.12	65	22.11	100
	Female	23	11.56	5	2.51	110	55.28	8	4.02	0	0	8	4.02	45	22.61	100
South America	Male	10	5.41	46	24.86	43	23.24	8	4.32	0	0	7	3.78	71	38.38	100
	Female	6	3.92	29	18.95	48	31.37	7	4.58	2	1.31	13	8.50	48	31.37	100
Oceania	Male	3	33.33	0	0	0	0	0	0	0	0	0	0	6	66.67	100
	Female	1	16.67	0	0	0	0	0	0	0	0	0	0	5	83.33	100

Female—(**b**) Venezuela (2), Argentina (3), Colombia (4–47), Ecuador (48–50), Brazil (51–154). Male and female—(**c**) Map 1: Male—Australia (2–7), Guam (8–9), New Zealand (10). Female—Australia (11–15), New Zealand (16).

A distribution of discovered clusters (7 in this case) by gender, across the various continents under study, is presented on Table 5. Notice that cluster 7 has the highest proportion of data points, indicating increased intra-country transmissions; save North America, where cluster 3 has the highest proportion of data points, an indication of increased inter-country transmissions. A further analysis across the continents reveals that the African, Asian, and South American isolates clustered around sub-strains G1, G2 and G5 (where G represents a generic/general sub-strain) with number of isolates and cluster proportions for male and female patients distributed as follows:

- Africa—G1: 186 (M = 20.22%, F = 23.27%), G2: 185 (M = 23.18%, F = 20.75%), and G5: 89 (M = 6.74%, F = 13.34%). The least sub-strains proportions come from Reference: 31 (M = 3.77%, F = 3.56%) and G4: 0 (M = 0%, F = 0%).
- Asia—G1: 255 (M = 27.31%, F = 24.04%), G2: 168 (M = 17.47%, F = 16.36%), and G5: 150 (M = 16.47%, F = 13.74%). The least sub-strains proportions come from cluster 4: 27 (M = 1.61%, F = 3.84%) and G4 (M = 0.40%, F = 0.20%).
- South America—G1: 75 (M = 24.86%, F = 18.95%), G2: 91 (M = 23.24%, F = 31.37%) and 6: 20 (M = 3.78%, F = 8.50%). The least sub-strains proportions come from cluster 4: 15 (M = 4.32%, F = 4.58%) and G4: 2 (M = 0%, F = 1.31%).
  European and North American isolates clustered around the Reference genome, the G1 and G2 sub-strains, with number of isolates and cluster proportions for male and female patients distributed as follows:
- Europe—cluster 1: 80 (M = 13.18%, F = 13.78%), cluster 2: 185 (M = 34.73%, F = 27.21%) and cluster 3: 45 (M = 4.18%, F = 11.31%). The least sub-strains proportions come from cluster 4: 17 (M = 2.57, F = 3.18%) and cluster 5: 12 (M = 0.64, F = 3.53%).
- North America—cluster 1: 50 (M = 9.18%, F = 11.56%), cluster 2: 79 (M = 25.17%, F = 2.51%) and cluster 3: 113 (M = 35.03%, F = 55.28%). The least sub-strains proportions come from cluster 4: 15 (M = 2.38%, F = 4.08%) and cluster 5: 0 (M = 0%, F = 0%).

Due to paucity of data, the Oceanian isolates have data for only cluster 1: 2 (M = 24.86%, F = 18.95%). Table 6 summarizes the clusters distribution, by gender across the various continents.

**Table 6:** Mean values and standard deviation of model performances on the male dataset.

k	Classification accuracy	RMSE	MAE	Precision	recall	AUC
3	98.5900 ± 0.7600	0.0500 ± 0.0200	0.0100 ± 0.00	0.9900 ± 0.0300	0.9700 ± 0.0400	1.00 ± 0.00
5	98.5900 ± 0.7600	0.0500 ± 0.0200	0.0100 ± 0.00	0.9900 ± 0.0300	0.9700 ± 0.0400	1.00 ± 0.00
10	98.5900 ± 0.7600	0.0500 ± 0.0200	0.0100 ± 0.00	0.9900 ± 0.0300	0.9700 ± 0.0400	1.00 ± 0.00
15	98.5900 ± 0.7600	0.0500 ± 0.0200	0.0100 ± 0.00	0.9900 ± 0.0300	0.9700 ± 0.0400	1.00 ± 0.00

### Cognitive knowledge generation

While mutations are expected, there is need to initiate robust surveillance mechanism for continuous monitoring of public health implications and rapid response to new strains of COVID-19. To intelligently predict the viral sub-strains for both genders, novel cognitive maps that preserves chains of similar isolates were generated from the SOM component planes using the Python programming language. The extracted clusters are necessary for supervised labeling of the classification targets. By disassembling the SOM correlation hunting matrix space, we attribute these associations to disparate classes of discovered viral sub-strains. The outcome are cognitive maps with 7 clusters simulating the discovered SOM patterns and countries/isolates linked to these patterns for male and female patients (Supplementary Table S3). Each sub-strain cluster holds similar isolates that belong to a related pattern bounded by certain degree of association or correlation range, established by the SOM, and captures all isolates discovered within this range. We also captured from the SOM component planes any progression in patterns showing sub-strain(s) development leading to well separated cluster image(s). The cognitive knowledge would assist early contact tracing of cases in emerging disease situations as well as establish how the reference genome has evolved over time. This additional knowledge also permits further characterization of the viral sub-strains, as our results allow unique SARS-CoV-2 base pairs sequence identification (which do not appear in other viral sub-strains) and could be useful as baselines for designing new primers that permit further insights and examination by experts.

### ANN performance evaluation

From the cluster patterns established by SOM, the datasets were labeled for further processing (see Male_SOM and Female_SOM worksheets of SupplData8.xlsx). To create a common scale and ensure that all input features have equal treatment in the model, the datasets were normalized using the minmax normalization technique [Eq. (1)]. The minimum and maximum components are obtained from the means of the nucleotide transitions or mutations features.

1 $$\frac{{x}_{i}-\mathrm{m}\mathrm{i}\mathrm{n}({\stackrel{-}{X}}_{j})}{\mathrm{max}\left({\stackrel{-}{X}}_{j}\right)-\mathrm{m}\mathrm{i}\mathrm{n}({\stackrel{-}{X}}_{j})}$$

where, ${x}_{i}$ is a nucleotide transition or mutation feature, ${\mathrm{m}}{\mathrm{i}}{\mathrm{n}}({\bar{X}}_{j})$ and ${\mathrm{m}}{\mathrm{a}}{\mathrm{x}}({\bar{X}}_{j})$ are the minimum and maximum means obtained from means of the respective nucleotide transitions or mutations feature dataset. The obtained scaling prevents zero values, hence, yielding an even spread of the datasets. Next, using the $\mathrm{k}$-means algorithm, via Silhouette criterion, 7 cluster groups were assigned to the records. These groups or clusters provided information for relabeling the cluster column of both datasets and constructing the output classification targets for supervised learning. The normalized datasets, newly formed clusters and classification targets for the male and female datasets are found in the Male_Normalized and Female_Normalized worksheets of SupplData8.xlsx.

The performance of the NN model was evaluated on the normalized, labelled datasets, using the following metrics: Classification Accuracy, Root Mean Squared Error (RMSE), Mean Absolute Error, Precision, Recall and Area Under the Curve (AUC). The metric specific result from each dataset compared using paired t-test, depict no statistically significant difference between the male and female features (p > 0.05) at the 0.05 level of significance. Results obtained on Tables 6 and 7 confirm the suitability of ANNs in predicting COVID-19 sub-strains for male and female patients, respectively. Furthermore, perfect accuracies with an AUC of 1 were obtained for k = 3, 5, 10 and 15 folds.

**Table 7:** Mean values and standard deviation of model performances on the female dataset.

k	Classification accuracy (%)	RMSE	MAE	Precision	Recall	AUC
3	98.5900 ± 0.7600	0.0500 ± 0.0100	0.00 ± 0.00	0.9900 ± 0.0100	1.00 ± 0.01	1.00 ± 0.00
5	98.6100 ± 0.7000	0.0500 ± 0.0100	0.0100 ± 0.00	0.9900 ± 0.0300	1.00 ± 0.01	1.00 ± 0.00
10	98.6100 ± 0.7000	0.0500 ± 0.0100	0.00 ± 0.00	0.9900 ± 0.0100	1.00 ± 0.01	1.00 ± 0.00
15	98.6100 ± 0.7000	0.0500 ± 0.0100	0.00 ± 0.00	0.9900 ± 0.0100	1.00 ± 0.01	1.00 ± 0.00

**Figure 9:**
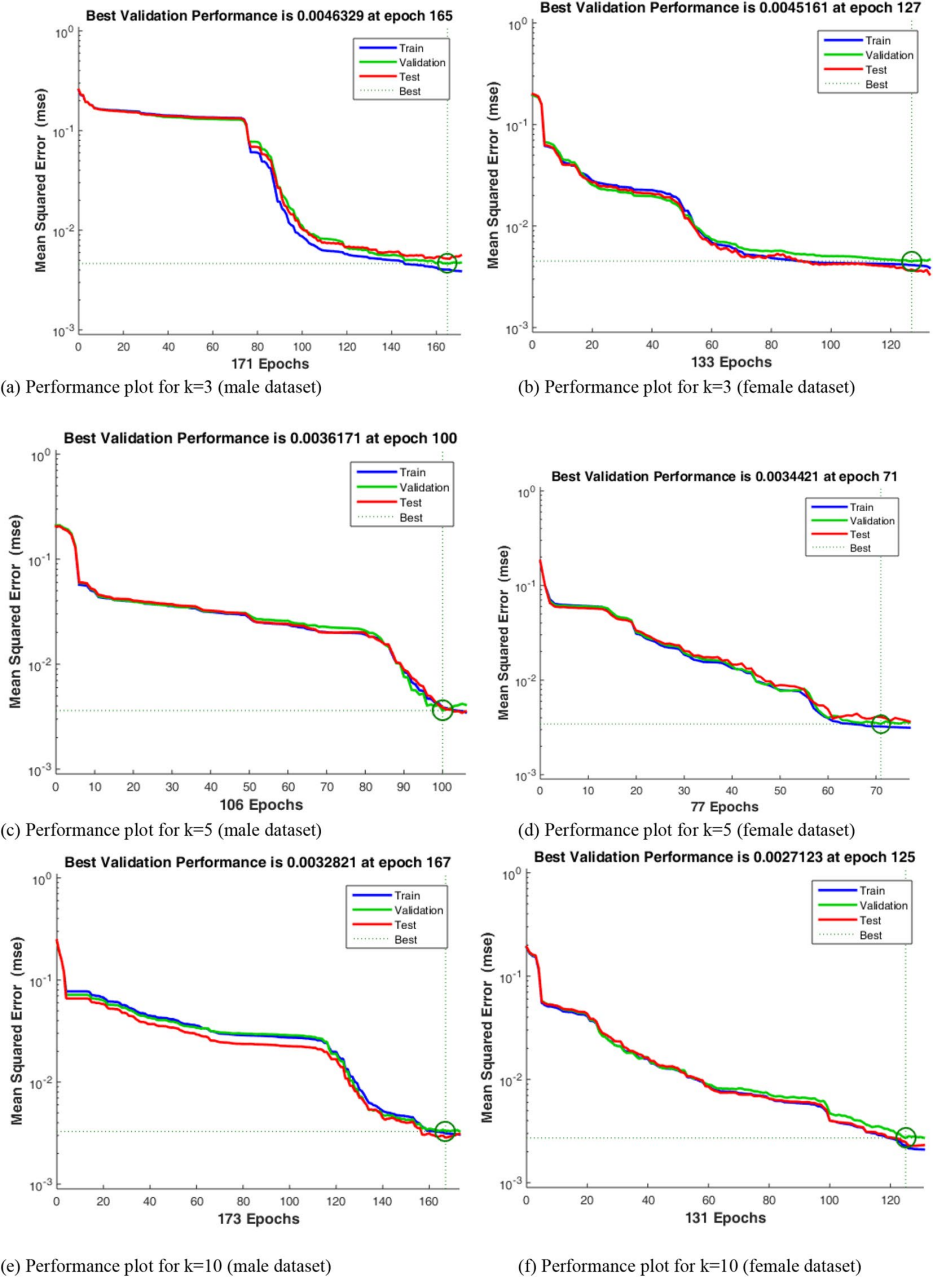

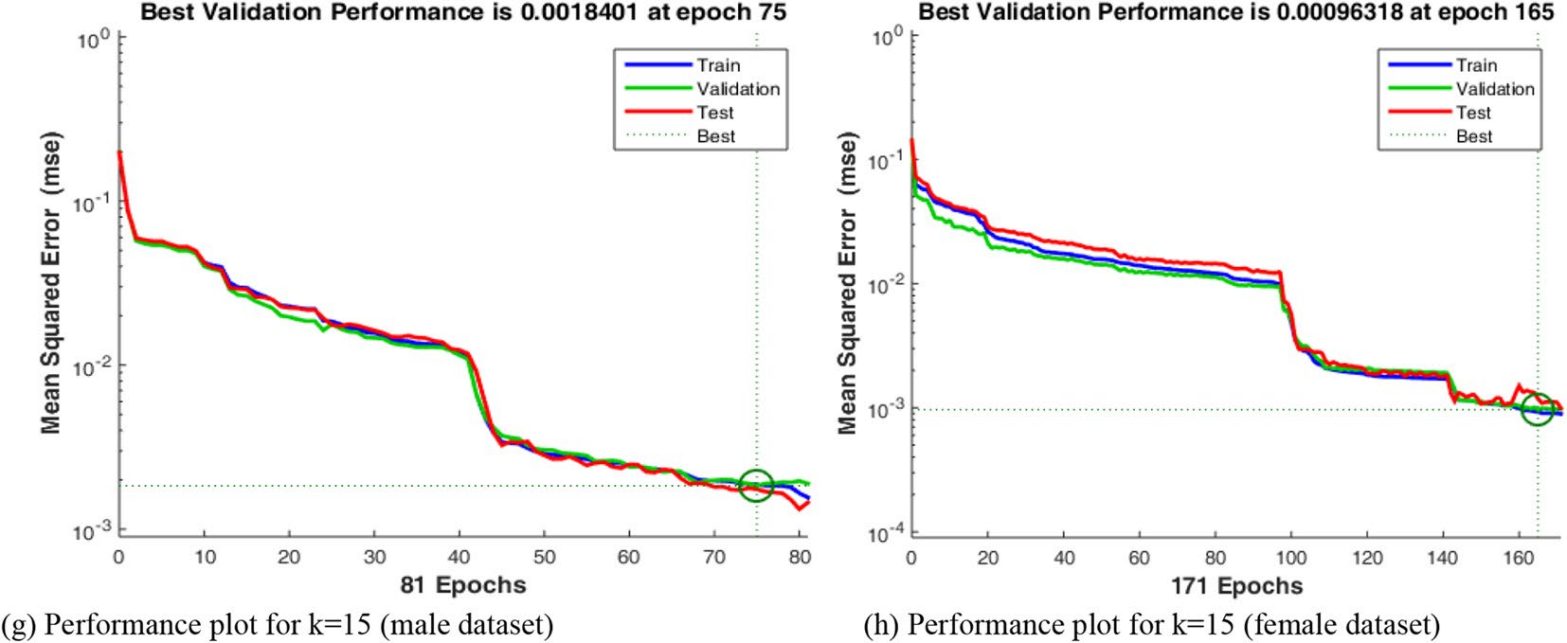
Neural network performance plots.

Using the mean squared error (MSE) function, the NN performance plots yielded best validation performance, with RMSE values of the different k-folds, for male and female datasets, derived as follows k = 3: M = 0.06807 (Fig. 9a), F = 0.0672 (Fig. 9b); k = 5: M = 0.0601 (Fig. 9c), F = 0.0587 (Fig. 9d); k = 10: M = 0.0573 (Fig. 9e), F = 0.0521 (Fig. 9f); k = 15: M = 0.0430 (Fig. 9g), F = 0.0310 (Fig. 9h). These results indicate improved classification errors as the number of validation folds increase.

**Figure 10:**
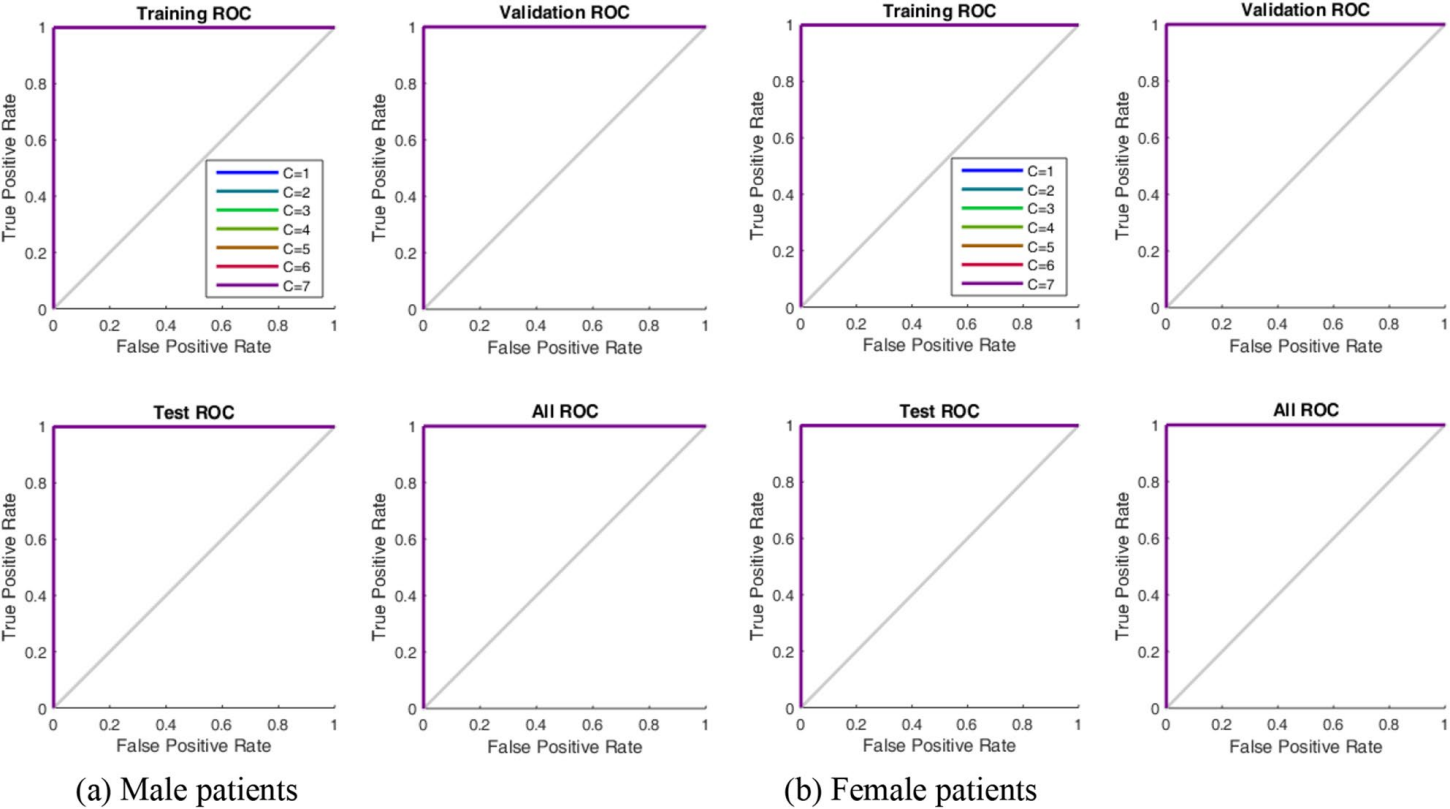
Receiver operation characteristics for k = 3, 5, 10, 15.

A receiver operation characteristics curve (ROC) windows showing the training, validation, test, and all ROC, with k = 3, 5, 10 and 15, for male and female patients are given in Fig. 10a and b, respectively. The deployed model is helpful for classifying new datasets and for building expert support systems for efficient SARS-CoV-2 sub-strains discrimination.

On Table 8, a summary of important performance metrics extracted from the literature for ANN with or without cross validation method, is presented to enable a comparison of our approach with state-of-the-art. We observe that the proposed approach performed better with very high classification accuracy, precision, and recall rates, indicating good generalization and correct prediction.

**Table 8:** Summary of performance metrics from previous works.

Reference	k-fold method	Classification accuracy (%)	RMSE	Precision	Recall	F1-Score %	AUC %
^40^	–	72.3300	–	0.7241	0.7167	0.72030	–
^42^	–	From Asia (67.0000) otherwise (86.0000)	–	–	–	–	–
^37^	Tenfold	76.9000	–	–	–	–	–
^21^	Tenfold	93.5000	–	–	–	–	–
^35^	Tenfold	90.0000	–	–	–	–	0.9200

## Conclusion

AI-based Big Data analytics are offering promising applications through the processing of large and complex datasets. In clinical diagnostics, for instance, image processing and computer vision are revolutionizing image-based diagnosis. In the field of genetics, large-scale genomic research is poised to improve care through genotype definitions of other organisms. The increased availability of multiscale, multimodal, longitudinal patient datasets has provided exclusive opportunities for individualized medicine by permitting the visualization of different patient dimensions. Although this is widely believed to enhance the performance of predictive algorithms for near-clinical practice, these data are highly unstructured and require further refinements to enable structured access and intelligent features combination.

The future of individualized medicine has however imposed limitations, challenges, and biases, as machine learning models are typically sensitive to selection biases (i.e., under- or over-represented specific patient subgroups in the training cohort, including under-explored ethical considerations), and have contributed to stiffening successful deployment of AI in medical applications, particularly those utilizing human genetics and genome datasets. Although addressing underrepresented data in training datasets can resolve bias, while model retraining can assist in improving performance; confusable symptoms relative to the disease have posed a major bottleneck for future applications. This work has created a foundation for future studies on emerging infectious diseases by investigating the variation and functions of SARS-CoV-2 genomes for possible discovery of patterns exhibited by human isolates. A novel taxonomy was created to permit intelligent features mining. The case of symptomatic and asymptomatic patients also presents inconsistencies and is inconclusive in this paper. This aspect of infectious disease demands further research efforts on prompt detection of asymptomatic cases. A major limitation of this research is that some SOM pattern clusters were still confused and demands a defuzzification of these clusters using robust neuro-fuzzification tools.

## Methods

### Data source and genome sequences selection

Publicly available datasets of coronavirus cases around the globe deposited between December 2019 and January 15, 2021 were excavated from GISAID (https://gisaid.org—a database of SARS-CoV-2 partial and complete genome compilations distributed by clinicians and researchers, the world over). Using the EpiCoV query interface of GISAID, complete genome sequences with patient status information (gender and age) were filtered. We observed that not all the excavated isolates met this criterion. Hence, out of about 70,000 entries, 8864 isolates (5130 male samples, and 3734 female samples) from different countries of the world contained at least the gender information, and were collected and processed, across 6 continents, Antarctica exempt (as no deposit of SARS-CoV-2 data was found as at the time of excavation). Age range of 1 month and 107 years were collected. Complete genome lengths of above 29,000 bp with < 1% undefined or ambiguous bases (‘N’s) or with high coverage unambiguous bases or nucleotides, were excavated from 88 different countries distributed across the following continents: Africa (Data S1: SupplData1.xlsx), Asia (Data S2: SupplData2.xlsx), Europe (Data S3: SupplData3.xlsx), North America (Data S4: SupplData4.xlsx), South America (Data S5: SupplData5.xlsx), and Oceania (Data S6: SupplData6.xslx).

**Figure 11:**
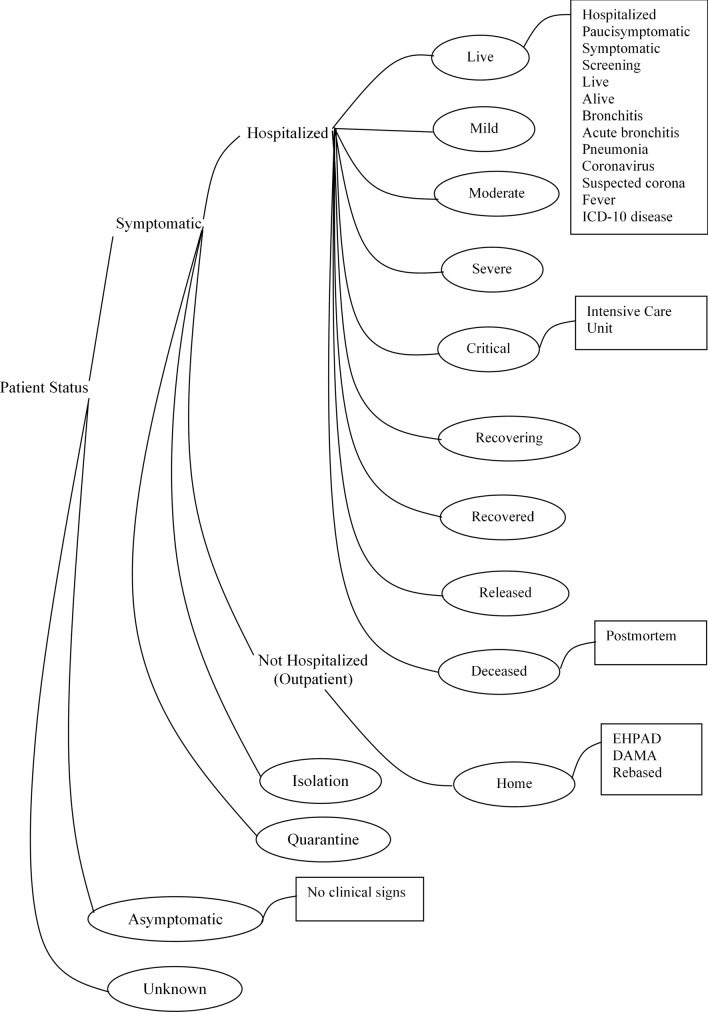
Reclassified GISAID COVID-19 patient status taxonomy.

Table 9 documents the continent, isolate distribution by country, isolate distribution by gender, and total isolates excavated. Metadata on the extracted genome sequences consisting of the following columns (Isolate Code: Country + isolate number, Country, Accession Number, Gender, Age, Status, Specimen source and Additional Information) were also documented (Data S7: SupplData7.xlsx). The Additional Information column holds both location and host information such as transmission history, treatment history, date sample was taken, etc. Fast-all (FASTA) files of the genome isolates can be located at GISAID using the Accession Number. Specimen sources include swabs (nasal, oral, throat, nasal and oral); fluids (bronchoalveolar lavage, saliva, sputum, stool) and unknown. We observed that the GSAID database was inconsistent in rendering the patient status, as numerous incoherent annotations introduced inherent redundancy. To assist efficient documentation and processing of data, a taxonomy re-classifying the patient status is given in Fig. 11. This taxonomy subsumes the incoherent annotations (annotations in square text boxes) into unique specifications (annotations in oval shapes), for intelligent data mining^48^.

**Table 9:** Distribution of excavated isolates.

Continent	Country	Male	Female	Total
Africa	Algeria (3), Cameroon (1), DRC (8), Egypt (35), Gambia (13), Ghana (15), Madagascar (3), Morocco (6), Mozambique (7), Nigeria (18), Rwanda (27), Senegal (135), South Africa (1507), Tunisia (26)	701	1103	1804
Europe	Andorra (1), Austria (18), Belgium (11), Bosnia and Herzegovina (4), Bulgaria (1), Croatia (15), Cyprus (8), Czech Republic (173), Denmark (3), Faroe Islands (14), Finland (2), France (131), Georgia (4), Germany (12), Greece (30), Hungary (80), Italy (561), Moldova (3), Norway (1), Poland (7), Portugal (2), Romania (52), Russia (125), Slovakia (4), Spain (256), Sweden (3), Switzerland (2), Ukraine (13)	802	743	1545
Asia	Bahrain (1), Bangladesh (29), Cambodia (1), China (319), India (1598), Indonesia (91), Iran (11), Iraq (2), Israel (38), Kazakhstan (24), Kuwait (3), Lebanon (18), Malaysia (89), Mongolia (6), Myanmar (1), Nepal (1), Oman (58), Pakistan (4), Philippines (12), Saudi Arabia (500), Singapore (540), South Korea (18), Sri Lanka (29), Taiwan (64), Thailand (2), Turkey (134), United Arab Emirates (111), Vietnam (74)	2618	1160	3778
South America	Argentina (2), Brazil (519), Chile (1), Colombia (186), Ecuador (28), Peru (2), Venezuela (3)	394	347	741
North America	Canada (27), Costa Rica (58), Dominican Republic (6), Guadeloupe (17), Mexico (110), Panama (253), Saint Martin (8), USA (499)	603	375	978
Oceania	Guam (2), New Zealand (2), Australia (14)	12	6	18
	Total: Number of countries excavated per continent: Africa (14), Europe (28), Asia (28), South America (7), North America (8), Oceana (3)	5130	3734	8864

The presence of ambiguous nucleotides may potentially mask the genomic signature encoded within nucleotide frequencies. Although sequencing errors in the form of ambiguous nucleotides (e.g., strings of letter “N”) were noticed in the datasets, the affected nucleotide positions were ignored during preprocessing, such that the nucleotide positions maintained their current position and did not shift. A total genome sequence size of ($8864\times 29000-8864\times 30165)\mathrm{b}\mathrm{p}\mathrm{s}=(\mathrm{257,056,000}-\mathrm{267,382,560})\mathrm{b}\mathrm{p}\mathrm{s}$ was excavated, processed, and stored in comma separated value (CSV) file.

Table 10 documents patient status statistics for symptomatic and asymptomatic cases. As observed, there are more hospitalized cases (7580) compared to non-hospitalized cases (391), with more male patients, hospitalized (M = 4318, F = 3262). Furthermore, more males died of COVID-19 than females (M = 541, F = 248). Asymptomatic cases however represent (37/5130; 0.72%) and (41/3734; 1.10%) of the total male and female isolates, respectively.

**Table 10:** Symptomatic and asymptomatic statistics.

Continent	Symptomatic																Not Hospitalized		Deceased		Asymptomatic	
	Hospitalized																					
	Live		Released		Recovering/recovered		Mild		Moderate		Severe		Critical		Quarantine/isolate		Home					
	M	F	M	F	M	F	M	F	M	F	M	F	M	F	M	F	M	F	M	F	M	F
Africa	599	1039	97	63	1	0	0	0	0	0	0	0	0	0	2	0	0	0	2	1	0	0
Asia	1737	728	623	327	29	16	37	25	0	0	0	0	5	1	5	2	0	0	182	61	0	0
Europe	441	436	34	31	35	43	122	109	32	21	35	17	4	6	1	0	25	19	37	26	32	33
North America	165	123	96	61	2	0	0	0	0	0	0	0	4	3	0	0	159	120	173	62	4	6
South America	100	109	68	66	27	29	0	0	0	0	0	0	7	1	0	0	33	33	147	98	1	2
Oceania	1	2	0	0	9	4	0	0	0	0	0	0	0	0	0	0	2	0	0	0	0	0
Total:	3043	2437	918	548	103	92	159	134	32	21	35	17	20	11	8	2	219	172	541	248	37	41
	Total hospitalized (M = 4318; F = 3262)																					

### Configuration of computing device

An HP laptop 15-bs1xx with up to 1 TB storage running on Windows 10 Pro Version 10.018326 Build 18,362 was used for processing the excavated genome sequences, algorithms/programs, and other ancillary data. The system has an installed memory (RAM) of 16 GB with the following processor configuration: 1.60 GHz, 1801 MHz, 4 Core(s) and 8 logical processors. Although our system performed satisfactorily and produced the desired results, higher system configurations would improve the computational speedup.

### Hierarchical agglomerative clustering (HAC)

The dataset is configured with observations (nucleotides) represented in rows, while columns are variables (genome sequences ordered by countries). The number of columns corresponds to selected countries while the sequences have varying lengths. The data table is further converted into *as.matrix* format where all values of raster layers objects have columns for each layer and rows for each cells with numeric (continuous) values. In order to make the variables comparable through the elimination of arbitrary variable units, they are transformed (standardized) such that they have mean of zero and standard deviation of unity^49^, using Eq. (2).

2 $$x\left(s\right)={x}_{i}-\frac{mean\left(x\right)}{sd\left(x\right)},$$

where $sd(x)$ represents the standard deviation of the feature values.

The procedure for implementing the HAC are as follows: Compute all the pairwise similarities (distances) between observations in the dataset and represent the result as a matrix. The resultant matrix is square and symmetric with diagonal members defined as unity–the measure of similarity between an element and itself. The matrix elements are computed by iterating over each element and calculating its (dis)similarity to every other element. Suppose $A$ is a similarity matrix of size $N\times N$, and $B$, a set of $N$ elements. ${A}_{ij}$ is the similarity between elements ${B}_{i}$ and ${B}_{j}$ using a specified criterion (Euclidean distance, squared Euclidean distance, manhattan distance, maximum distance, Mahalanobis distance, cosine similarity). The selected criterion however depends on the nature of the experimental datasets. This paper adopts the standardized Euclidian distance criterion, as this measure is widely used and has shown good performance in the modeling variances in biological sequences.

### HAC visualization

After computing the distance between every pair of observation point, the result is stored in a distance matrix. Then, (i) every point is put in its own cluster (i.e., the initial number of clusters corresponds to the number of variables); (ii) the closest pairs of points are merged based on the distances from the distance matrix as the number of clusters reduces by 1; (iii) the distance between the new cluster and the previous ones is recomputed and stored in a new distance matrix; (iv) steps (ii) and (iii) are repeated until all the clusters are merged into one single cluster.

**Figure 12:**
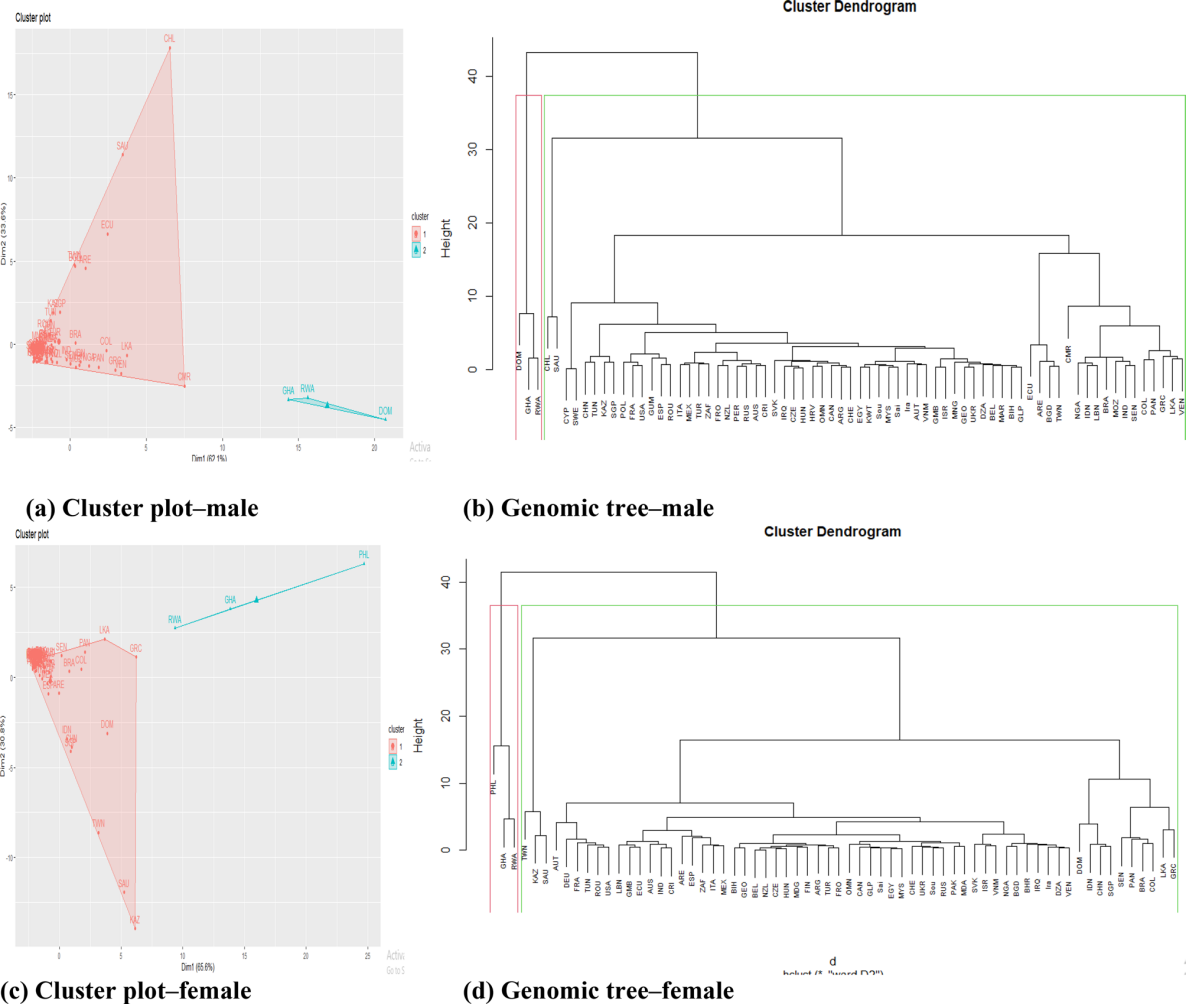
Cluster plots and genomic trees. Notice 2 distinct groups (or clusters) A and B separated between closely similar and dissimilar isolates, with the A group having heavy isolates concentration than the B group. For males (**b**), group A consists of 68 isolates with 7 sub-groups as follows: 1 (CHL, SAU); 2 (CHN, TUN, KAZ, SGP, POL, FRA, USA, GUM, ESP, ROU); 3 (ITA, MEX, TUR, ZAF, FRO, NZL, PER, RUS, AUS, CRI); 4 (SVK, IRQ, CZE, HUN, HRV, OMN, CAN, ARG, CHE, EGY, KWT, SOU, MYS, SAI, Iran, AUT, VNM, GMB, ISR, MNG, GEO, UKR, DZA, BEL, MAR, BIH, GLP); 5 (ECU, ARE, BGD, TWN); 6 (CMR, NGA, IDN, LBN, BRA, MOZ, IND, SEN, COL, PAN, GRC, LKA, VEN). Group B consists of 1 sub-group as follows: 1 (DOM, GHA, RWA). For Females (**d**), group A consists of 63 isolates with 6 sub-groups as follows: 1 (TWN, KAZ, SAU); 2 (AUT, DEU, FRA, TUN, ROU, USA); 3 (LBN, GMB, ECU, AUS, IND, CRI, ARE, ESP, ZAF, ITA, MEX); 4 (BIH, GEO, BEL, NZL, CZE, HUN, MDG, FIN, ARG, TUR, FRO, OMN, CAN, GLP, SAI, EGY, MYS, CHE, UKR, SOU, RUS, PAK, MDA, SVK, ISR, VNM, NGA, BGD, BHR, IRQ, Iran, DZA, VEN); 5 (DOM, IDN, CHN, SGP); 6 (SEN, PAN, BRA, COL, LKA, GRC).

The distance separating the clusters is specified via linkage methods^49^ which includes, complete, average, single, and ward. Complete linkage computes the similarities and uses the maximum distance between clusters for merging while calculating cluster distances and adopting minimum inter-cluster distance merging. The average linkage calculates the average distance between groups of genome sequence before merging; while the total within-cluster variance is minimized with ward’s method and the pair of clusters with minimum between-cluster distance are merged. We rely on all the four assessment techniques and adopt the distance measure with the highest agglomerative coefficient for cluster formation. The resultant cluster solution is finally visualized as a tree structure called a dendrogram (or phylogenomic) tree. As the tree is traversed upwards, observations that are similar to each other are combined into branches, which are themselves fused at a higher height. The height of the fusion provided on the vertical axis, indicates the (dis)similarity between two observations. The higher the height of the fusion, the less similar the observations are. Figure 12 show cluster plots and genomic plots generated using the ward minimum variance criterion.

### Optimal natural clusters selection

While there are natural structural entities in some datasets that provide information on the number of clusters or classes, others including the dataset containing genome sequences are structured without boundaries. Cluster validation (an unsupervised methodology aimed at unravelling the actual count of clusters that best describes a dataset without any priori class knowledge) is therefore essential. In this paper, three widely used criteria to validate the number of clusters in the genome sequence dataset of these widely used criteria namely, silhouette, elbow^50^, and gap-statistics are discussed. The three criteria aim at minimizing the total intra-cluster variation (total within-cluster sum of square) as given in Eq. (3).

3 $$minimize\left(\sum _{i=1}^{k}w({c}_{k})\right)$$

where ${c}_{k}$ is the kth cluster, and, ${w(c}_{k})$ is the within-cluster variation. The total within-cluster sum of squares (wss) measures the compactness of the clustering solution. The following steps are applied to achieve the optimal clusters: (i) Compute using clustering algorithm (e.g., k-means clustering) for different values of $k$; by varying $k$ for a range of cluster values. (ii) For each $k$, calculate wss. (iii) plot the curve of wss according to the number of clusters $k$. (iv) the location of a bend (knee) in the plot is generally considered as an indicator of the appropriate number of clusters. Silhouette criterion is used to validate the clustering solution using pair-wise difference between the within-cluster distances, and by maximizing the value of this index to arrive at the optimal cluster number^51^. Elbow criterion plots the variance resulting from plotting the explained variation as a function of the number of clusters and picking the elbow of the curve as the number of clusters to use. Gap-statistics compares the total intra-cluster variation for different values of $k$ with their expected values under null reference distribution of the data. The reference dataset is generated using Monte Carlo simulations of the sampling process.

In this paper, the k-means algorithm^52^ is implemented using the R script consisting of R functions for the silhouette, elbow, and gap-statistics. The clustering solution can be visualized using the *fviz_cluster* function in R, to group the extracted genome sequences and finally represent the groupings in a tree format using dendrogram. As a preprocessing step to study the phylogeny of the genome isolates, the HCA or AGNES plots as shown in Fig. 12a and c, reveal for the male and female groups, respectively, two natural clusters A and B, suggesting more countries with viral strains of close lineage (group A), while few mild divergent strains (group B) with specific mutations are geographically different. Hence, for the male isolates, the phylogenomic tree (Fig. 12b) grouped 68 isolates/countries under cluster A, while the remaining 3 isolates belong to cluster B. For the female isolates (Fig. 12d), 63 isolates belonged cluster A while the remaining 3 isolates were grouped under cluster B.

### Genome features extraction

#### Dinucleotide transition frequency

The SARS-CoV-2 reference genome^53^ (Severe acute respiratory syndrome coronavirus-2 isolate Wuhan-Hu-1, complete genome) obtained from the NCBI: www.ncbi.nlm.nih.gov) contains 4 conventional DNA nucleotide bases, $\mathrm{A}, \mathrm{C}, \mathrm{G}, \mathrm{T}$. Hence, there are ${4}^{2}=16$ unique dinucleotide pairs that can be constructed from these bases, namely:

4 $$\omega =\{\mathrm{A}\mathrm{A},\mathrm{A}\mathrm{C}, \mathrm{A}\mathrm{G},\mathrm{A}\mathrm{T}, \mathrm{C}\mathrm{A}, \mathrm{C}\mathrm{C},\mathrm{C}\mathrm{G},\mathrm{C}\mathrm{T},\mathrm{G}\mathrm{A}, \mathrm{G}\mathrm{C},\mathrm{G}\mathrm{G},\mathrm{G}\mathrm{T}, \mathrm{T}\mathrm{A}, \mathrm{T}\mathrm{C}, \mathrm{T}\mathrm{G},\mathrm{T}\mathrm{T}\}$$

If we denote the frequency of the $\mathrm{i}\mathrm{t}\mathrm{h}$ dinucleotide as ${d}_{i}$, then, a genomic sequence with 16-dimensional feature vector in the form of Eq. (5) are possible,

5 $${f}_{\omega }=\{{\mathrm{d}}_{\mathrm{A}\mathrm{A}},{\mathrm{d}}_{\mathrm{A}\mathrm{C}},{\mathrm{d}}_{\mathrm{A}\mathrm{G}},\dots ,{\mathrm{d}}_{\mathrm{T}\mathrm{T}}\}$$

The frequencies of the dinucleotide transitions are obtained by accumulating each dinucleotide along the extracted genome sequences. We ignore ambiguous nucleotides absent in the reference genome. Suppose we have $n$ total genome length. By allowing a single sliding iteration window there exists $n-1$ bubble counts. Hence, the dinucleotide frequencies of ${d}_{i}$ can be obtained by counting all nucleotides that correspond to $i.$

#### Nucleotide mutation frequency

Several techniques for biological sequence alignment (multiple or pairwise) have flourished the literature^54^ and are continually being refined, but most of these techniques suffer from the lack of accuracy and partial interpretations. A direct pairwise alignment of each nucleotide with the reference genome was achieved by computing the recurrence of mutated nucleotides down the sequence line. For this study, the sequence of established SARS-CoV-2 reference genome (NC_045512; 29903 bp) sequenced in December 2019 was used. Suppose $n$ represents the total length of a genome; By permitting a single sliding iteration window, a mutation may be any of the following pair:

6 $$\mathrm{m}=\{\mathrm{A}\mathrm{C}, \mathrm{A}\mathrm{G},\mathrm{A}\mathrm{T}, \mathrm{C}\mathrm{A}, \mathrm{C}\mathrm{G},\mathrm{C}\mathrm{T},\mathrm{G}\mathrm{A}, \mathrm{G}\mathrm{C},\mathrm{G}\mathrm{T}, \mathrm{T}\mathrm{A}, \mathrm{T}\mathrm{C}, \mathrm{T}\mathrm{G}\}$$

If we denote the frequency of the $\mathrm{i}\mathrm{t}\mathrm{h}$ nucleotide pair as ${p}_{i}$, then, genomic sequence pairs with 12-dimensional feature vector in the form of Eq. (7) are possible,

7 $${\mathrm{f}}_{\mathrm{m}}=\{{\mathrm{p}}_{\mathrm{A}\mathrm{C}},{\mathrm{p}}_{\mathrm{A}\mathrm{G}},{\mathrm{p}}_{\mathrm{A}\mathrm{T}},\dots ,{\mathrm{p}}_{\mathrm{T}\mathrm{G}}\}$$

#### Unsupervised genome clustering

**Figure 13:**
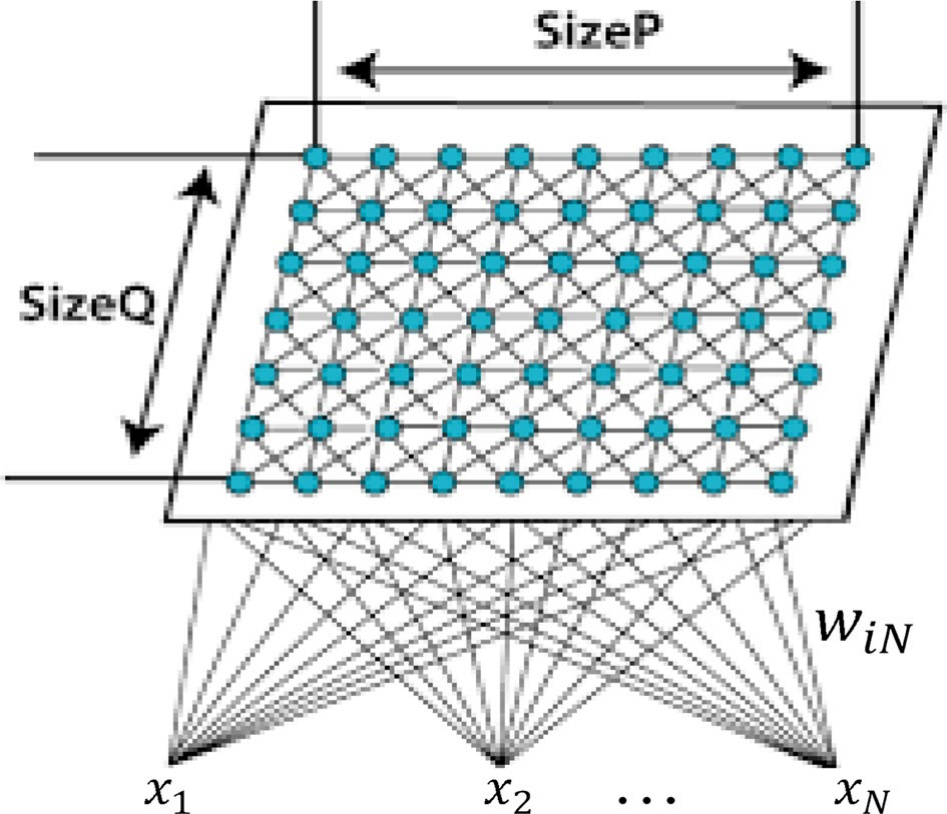
SOM showing the map topology and interactions between nodes. Each neuron is assigned a vector of weights ($w={w}_{i1},{w}_{i2},\ldots{w}_{iN}$) with dimension similar to the input vector $i(i=1,2,\dots,L$); where $L$ is the total number of neurons in the network. The input nodes have $p$ features, and the output nodes, $q$ prototypes, with each prototype connected to all features. The weight vector of the connections consumes the prototype of each neuron and has same dimension as the input vector. SOMs differ from other artificial neural networks as they apply competitive learning, against error correction learning such as backpropagation, and the fact that they preserve the topological properties of the input space using a neighborhood function.

Several mathematical techniques have been deployed for identifying underlying patterns in complex data. These techniques, which cluster data points differently in multidimensional space are important to discover fundamental patterns of gene expression inherent in data. The clustering technique adopted in this paper is the SOM and has been used extensively in the field of bioinformatics, for visual inspection of biological processes, genes pattern expressions–as maps of (input) component planes analysis. SOM is a neural-network that projects data into a low-dimensional space^55^, by accepting a set of input data and then mapping the data onto neurons of a 2D grid (see Fig. 13). The SOM algorithm locates a winning neuron, its adjusting weights, and neighboring neurons. Using an unsupervised, competitive learning process, SOMs produce a low-dimensional, discretized representation of the input space of training samples, known as the feature map. During training, weights of the winning neuron and neurons in a predefined neighborhood are adjusted towards the input vector using Eq. (8),

8 $${w}_{id}^{t+1}={w}_{id}^{t}+rf\left(i,q\right)\left({x}_{d}-{w}_{id}^{t}\right);1\le d\le D.$$

where $r$ is the learning rate and $f(i,q)$ is the neighborhood function, with value 1 at the winning neuron $q$; and decreases as the distance between $i$ and $q$ increases. At the end, the principal features of the input data are retained, hence, making SOM a dimension reduction technique. The batch unsupervised weight/bias algorithm of MATLAB (*trainbu*) with mean squared error (MSE) performance evaluation, was adopted to drive the proposed SOM. This algorithm trains a network with weight and bias learning rules using batch updates. The training was carried out in two phases: a rough training with large (initial) neighborhood radius and large (initial) learning rate, followed by a finetuned training phase with smaller radius and learning rate. The rough training phase can span any number of iterations depending on the capacity of the processing device. In this paper, we kept the number of iterations at 200 with initial and final neighborhood radius of 5 and 2, respectively, in addition to a learning rate in the range of 0.5 and 0.1. The fine training phase also had a maximum of 200 epochs, and a fixed learning rate of 0.2. Selection of best centroids of the genome feature within each cluster was based on the Euclidean distance criterion. The algorithm configures output vectors into a topological presentation of the original multi-dimensional data, producing a SOM in which individuals with similar features are mapped to the same map unit or nearby units, thereby creating smooth transition of related genome sequences to unrelated genome sequences over the entire map.

#### Genome sequence transformation and low similarity profile selection

Each genome sequence is mapped into an equivalent genomic signal (a discrete numeric sequence) using the following individual nucleotide encoding (i.e., A = 1; C = 2; G = 3; T = 4). Nucleotide pairs above 29,000 bp is maintained in this paper as base input vector, indicating approximate (maximum) length of DNA sequences of the raw SARS-CoV-2 genome. Next, repeated sequences are removed using a Microsoft Excel macro that deletes duplicate columns. A Microsoft Excel macro implementing this process is found on Supplementary Table S2. A similarity threshold of 0.90 is then imposed to further trim closely similar genomes, resulting in genomes with low similarity profiles or highly variable sequences distributed per continent and outlined in a component planes window, containing 88 isolates/countries shared according to gender as contained in SupplData8.xlsx (male = 71 countries; female = 66 countries), as follows (Africa: M = 371, F = 477; Asia: M = 514, F = 510; Europe: M = 311, F = 283; North America: M = 294, F = 199; South America: M = 185, F = 153; Oceania: M = 9, F = 6). The similarity threshold may be increased or reduced to grow or shrink the size of the component planes window. In this paper, a maximum window size of 250 component planes is allowed, to enable proper viewing of the pattern clusters (see Figs. 4, 5, 6, 7, 8 of the “Results and discussion” section).

#### Pattern correlates generation

A vector representation of pairwise Euclidean distance computation among the vectors in the form of a distance matrix (also called the component plane) is achieved using MATLAB 2017b. Component planes help detect similar patterns in identical positions indicating correlations between the respective components. Local correlations may also occur if two parameter planes are similar in some regions. Both linear and non-linear correlations including local or partial correlations between variables are possible. We achieve the correlation hunting^56^ automatically, by decoupling the SOM correlations, to explore patterns among the pairwise genome samples for distinct identification of transmission pathways or routes. The extracted correlation matrices are pairwise relations of the viral sub-strains’ transmissions.

#### Cognitive knowledge extraction

Knowledge mining has served huge benefits for quick learning from big data. We apply Natural Language Processing of the genome datasets to extract knowledge of similar strains of the virus. A simple iteration technique is imposed on the SOM isolates ($$i=\mathrm{1,2},3,\dots ,n)$$, where $$n$$ is the maximum number of isolates, as follows: For each isolate pattern, compile similar patterns with the rest of the isolates (i.e., $$i+1,i+2,\dots,n)$$. Concatenate compiled isolate(s) into a list ($${j}_{1},{j}_{2}$$,…,$${j}_{m}$$) where $$j$$ is an element of the list. Dump the compiled list into $$CogMap({k}_{i}\in {j}_{1},{j}_{2}$$,…,$${j}_{m})$$. As the distance matrix is extremely high-dimensional, suitable representative sequences of the isolate clusters are decoupled into a cognitive map for labeling of the classification targets.

#### Neural network design

**Figure 14:**
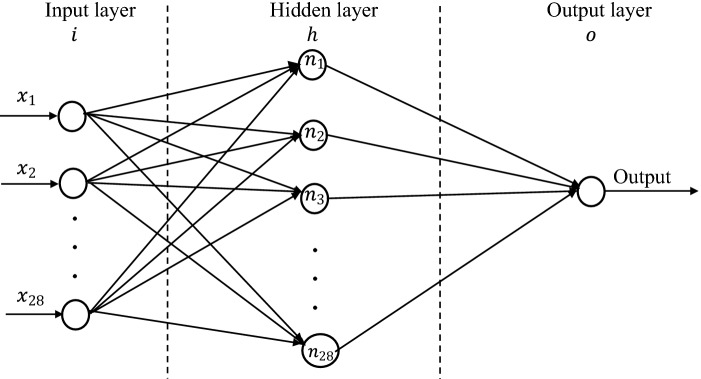
ANN architecture. A 3-layered network, with one output layer and one hidden layer. The input layer consumes the knowledge-enriched genome datasets comprising of extracted patterns of SOM learning of the respective genome isolates and additional knowledge sieved from analysis of the genome sequences (i.e., number of natural clusters discovered from the genomic tree, discovered SOM sub-strain clusters, and link sequences derived from cognitive maps of the various isolates).

Although five core Artificial Neural Networks (ANN) areas have been explored, namely: Multi-Layer Perceptron, Radial Basis Network, Recurrent Neural Networks, Generative Adversarial Networks, and Convolutional Neural Networks; this paper adopts the Multi-Layer Perceptron model (MLP)—a class of feedforward ANNs, with at least three layers of nodes: an input layer, a hidden layer, and an output layer (Fig. 14). Except for the input nodes, each node is a neuron that uses a nonlinear activation function. MLP utilizes a supervised learning technique called backpropagation for training. Our output classes or classification targets (C1-C7) are derived from pattern clusters discovered from learning the SOM. A $\mathrm{k}$-fold cross-validation method is adopted to divide the data into $\mathrm{k}$ parts. At each iteration $\mathrm{i}$, the $\mathrm{i}\mathrm{t}\mathrm{h}$ fold is used for testing, while the other folds are used for training. In this paper, the number of groups is split (into $\mathrm{k}$ parts) such that each data sample spans 3, 5, 10 and 15 yielding 60, 100, 200 and 300 calls, respectively, on the training and testing mode of each dataset. The $\mathrm{k}$-fold cross validation method is known to estimate the robustness of the model on new data and is used to drive the validation phase of the NN. As the model is fit on training data, a more realistic estimate of how well the model prediction will work on new cases is obtained. In the current experimental setup, twenty (20) runs of stratified k-fold cross validation^57^ is performed on the male and female datasets using a Neural Network (NN) model developed in the MATLAB2017b.

## Supplementary Information

Supplementary Information 1.

Supplementary Information 2.

Supplementary Information 3.

Supplementary Information 4.

Supplementary Information 5.

Supplementary Information 6.

Supplementary Information 7.

Supplementary Information 8.

Supplementary Information 9.

Supplementary Information 10.
