# Supplementary material for: A hybrid computational framework for intelligent inter-continent SARS-CoV-2 sub-strains characterization and prediction
Source: Sci Rep. 2021 Jul 15;11:14558. doi: 10.1038/s41598-021-93757-w (PMC8282786; doi:10.1038/s41598-021-93757-w)
Supplement: Supplementary file 1 — Supplementary Information 1. [file 41598_2021_93757_MOESM1_ESM.docx]

**Supplementary Table S1. Steps implementing the workflow in Fig. 1.**

| 1. #Genome nucleotide fragments processing 2. create a list of FASTA files (fasta_list) to process 3. *for* file_name in fasta_list: 4. store a line of genome sequence 5. *for* line in file_name: 6. strip line into a list of nucleotide fragments (nucleotide_fragments) 7. *for* line in nucleotide_fragments: 8. write nucleotide code into complete genome file (complete_genome) 9. #Direct nucleotide alignment and genome features extraction 10. open complete_genome for read 11. store a line of nucleotide code from complete_genome 12. #Extract dinucleotide transitions 13. *for* line in complete_genome: 14. compare previous and current nucleotide codes 15. accumulate dinucleotides frequency within acceptable transitions (AA, AC…) 16. #Extract dinucleotide mutations 17. *for* line in complete_genome: 18. align nucleotide pair with reference genome 19. accumulate nucleotide changes within acceptable mutations (AC, TA…) 20. #AGNES/hierarchical clustering: generate phylogenomic tree and cluster plots 21. treat observations (nucleotides) as cluster points and compute AGNES distance coefficients between clusters 22. compute scores between genome isolates clusters 23. build and visualize genomic tree 24. discover and validate optimal natural clusters (k) using any k-means based N approaches (N>2) (elbow, silhouettes, gap-statistics etc.). 25. partition the tree into k clusters 26. #Genome expression patterns discovery 27. perform SOM clustering on complete_genome 28. obtain SOM component planes of learned genome expression patterns 29. obtain pairwise correlation coefficients 30. label target (output) classes using genome expression clusters for mutant sub-strains and viral expression patterns, to form enriched genome datasets. 31. generate cognitive maps with embedded links of genome isolates. 32. learn and classify genome (isolate) patterns characterized by (generated) cognitive knowledge, using artificial neural network (ANN) with k-fold validation method. 33. predict SARS-CoV-2 sub-strain. |
| --- |

**Supplementary Table S2. Excel macro code implementing the delete duplicate columns**

| 1. Sub DeleteDuplicateColumns() 2. Dim rngData As Range 3. Dim arr1, arr2 4. Dim i As Integer, j As Integer, n As Integer 5. On Error Resume Next 6. Set rngData = ActiveSheet.UsedRange 7. If rngData Is Nothing Then Exit Sub 8. n = rngData.Columns.Count 9. For i = n To 2 Step -1 10. For j = i - 1 To 1 Step -1 11. If WorksheetFunction.CountA(rngData.Columns(i)) <> 0 And _ 12. WorksheetFunction.CountA(rngData.Columns(j)) <> 0 Then 13. arr1 = rngData.Columns(i) 14. arr2 = rngData.Columns(j) 15. If AreEqualArr(arr1, arr2) Then 16. With rngData.Columns(j) 17. 'mark column to be deleted 18. .Copy 19. If MsgBox("Delete marked column?", vbYesNo) _ 20. = vbYes Then 21. rngData.Columns(j).Delete 22. Else 23. 'remove mark 24. Application.CutCopyMode = False 25. End If 26. End With 27. End If 28. End If 29. Next j 30. Next i 31. End Sub 32. Function AreEqualArr(arr1, arr2) As Boolean 33. Dim i As Long, n As Long 34. AreEqualArr = False 35. For n = LBound(arr1) To UBound(arr1) 36. If arr1(n, 1) <> arr2(n, 1) Then 37. Exit Function 38. End If 39. Next n 40. AreEqualArr = True 41. End Function |
| --- |

**Supplementary Table S3. Novel cognitive map of identified cluster patterns and linked countries/isolates**

| Cluster | Discovered genome pattern | Associated (linked) isolate chain |
| --- | --- | --- |
| 1-Reference genome | Africa:  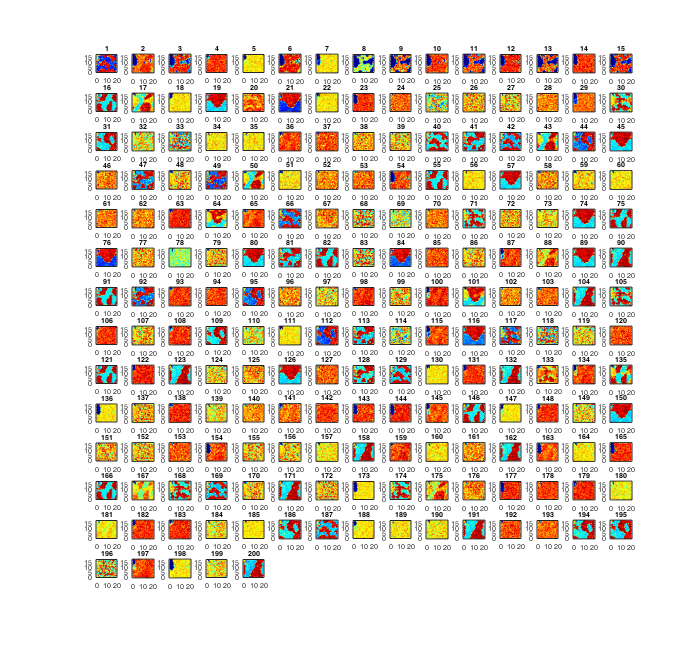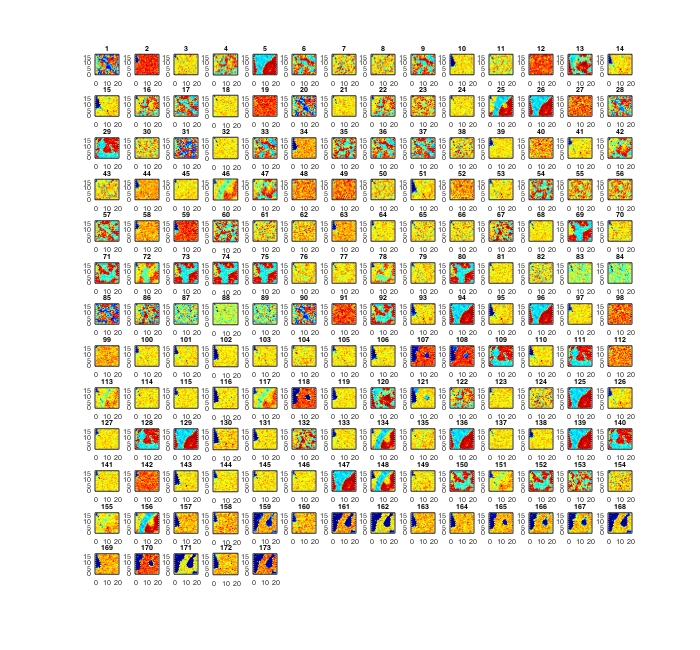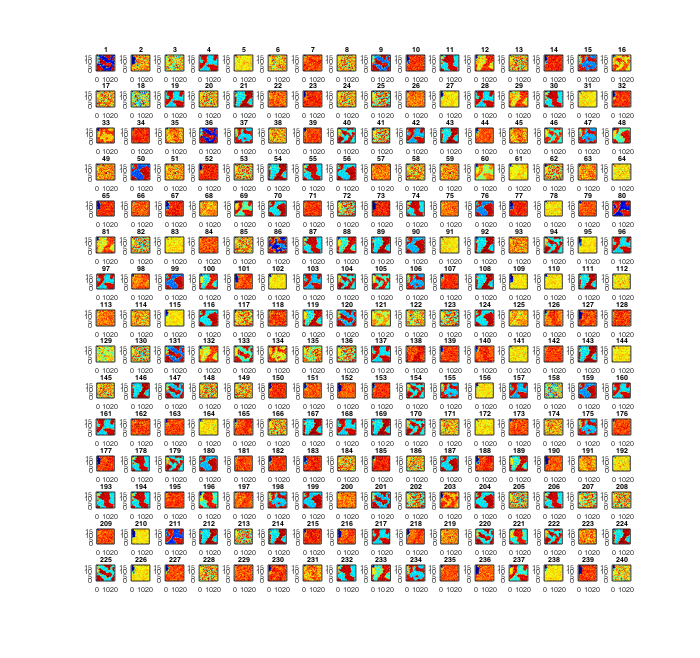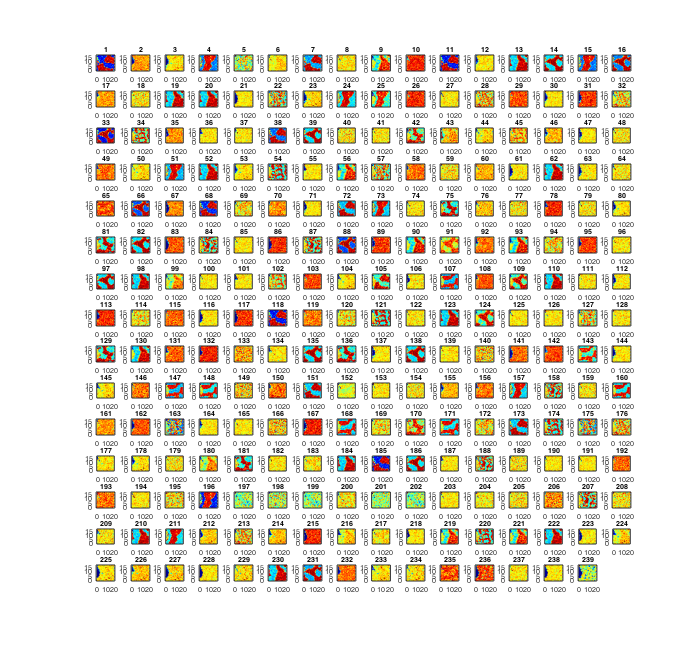  Asia:  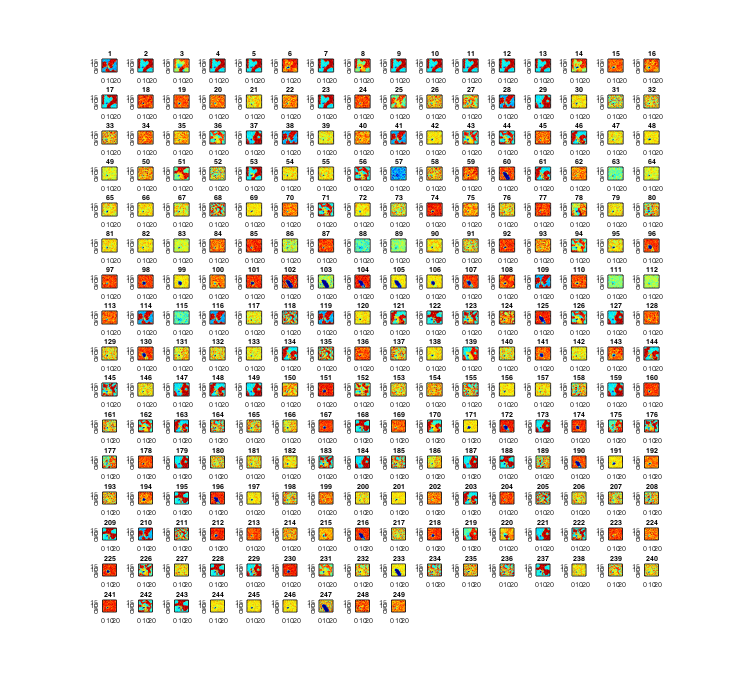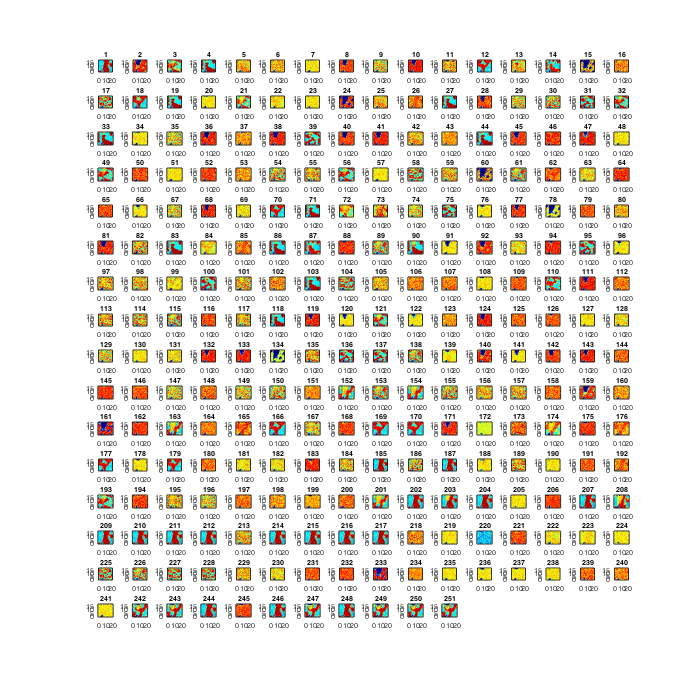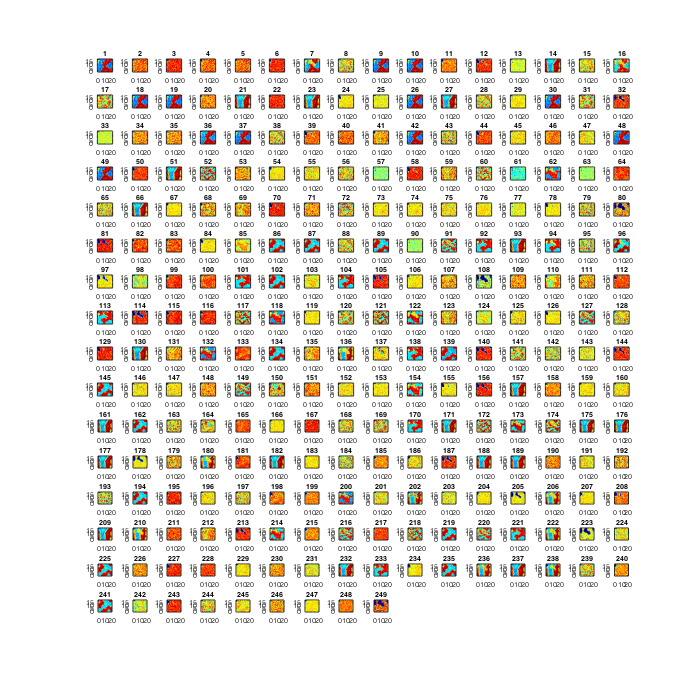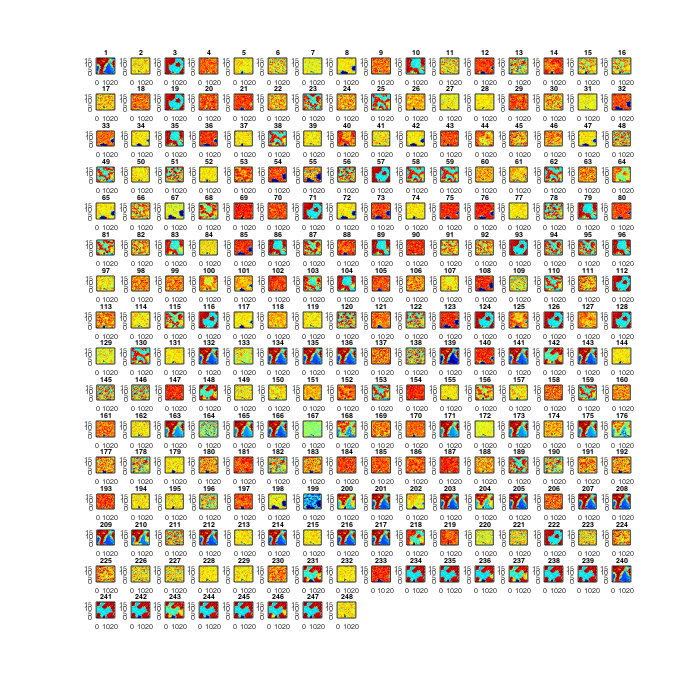  Europe:  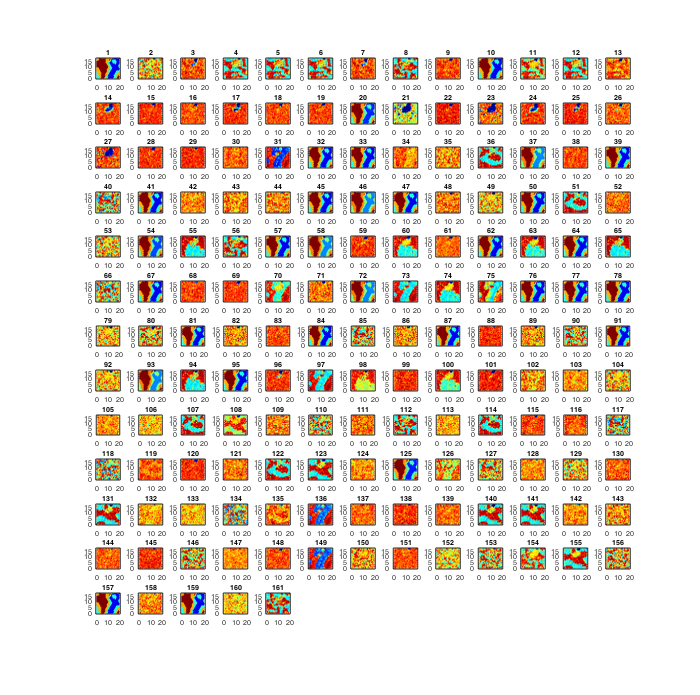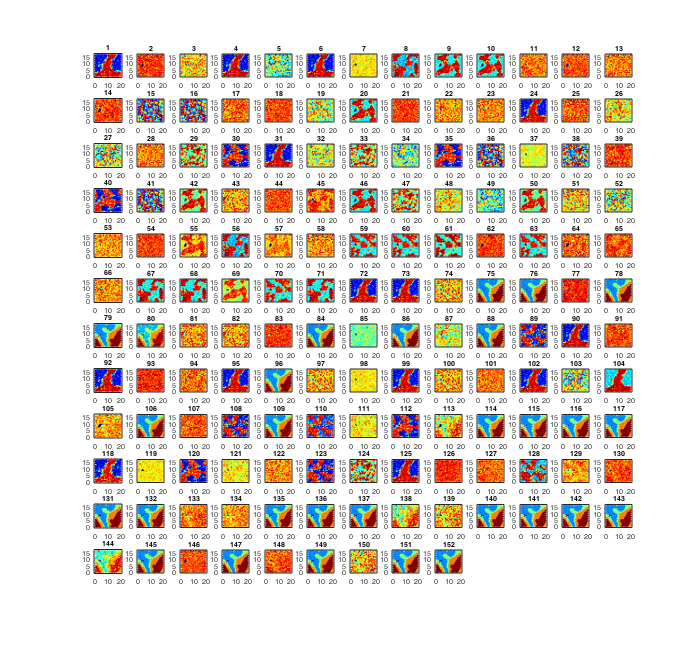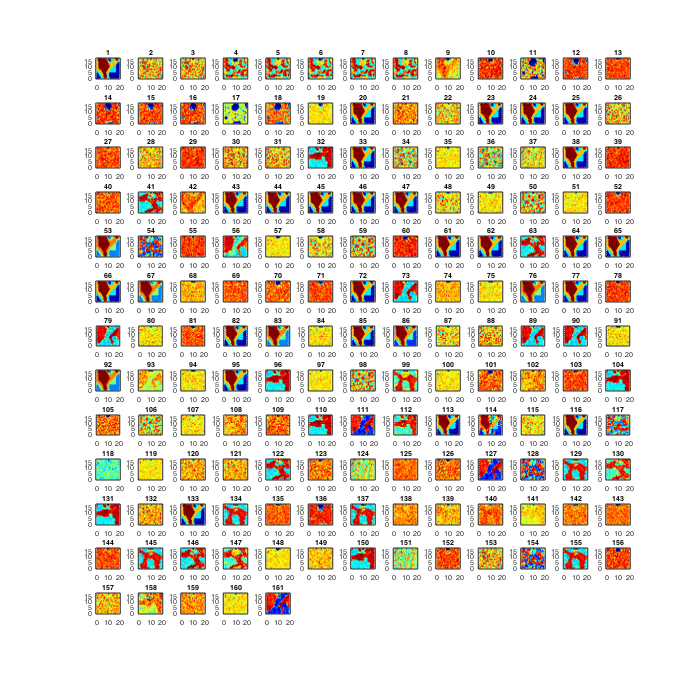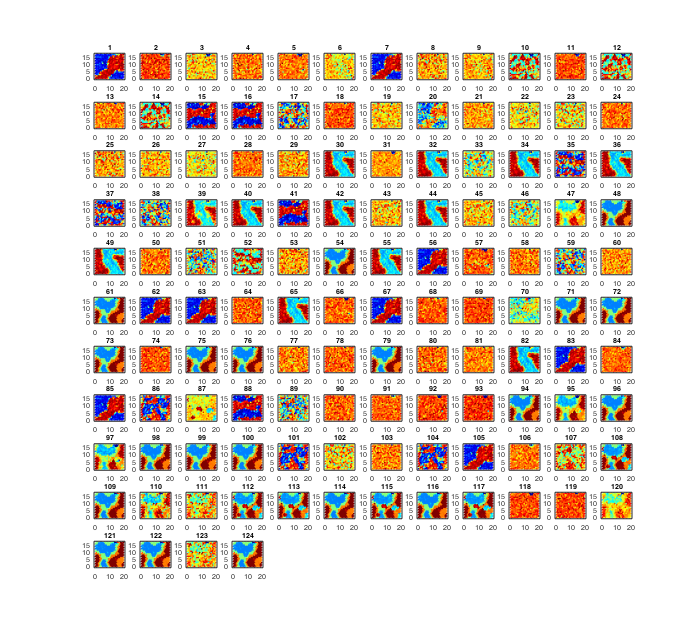  North America:  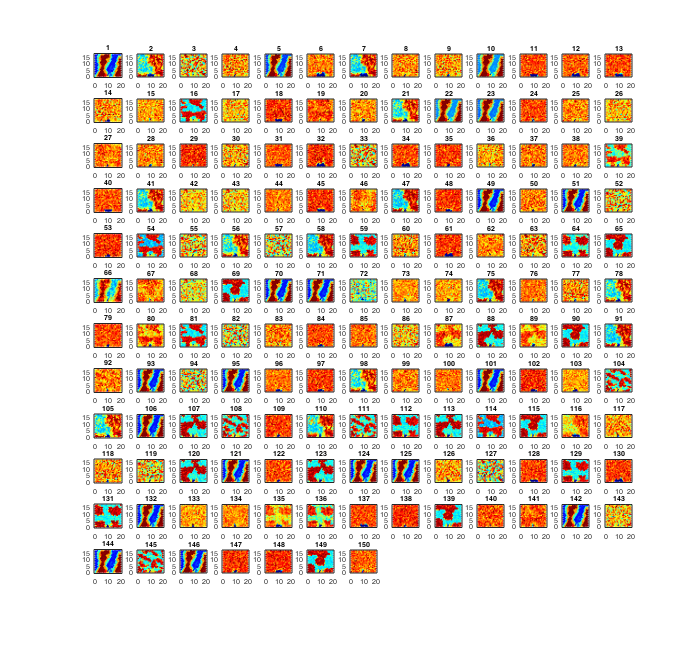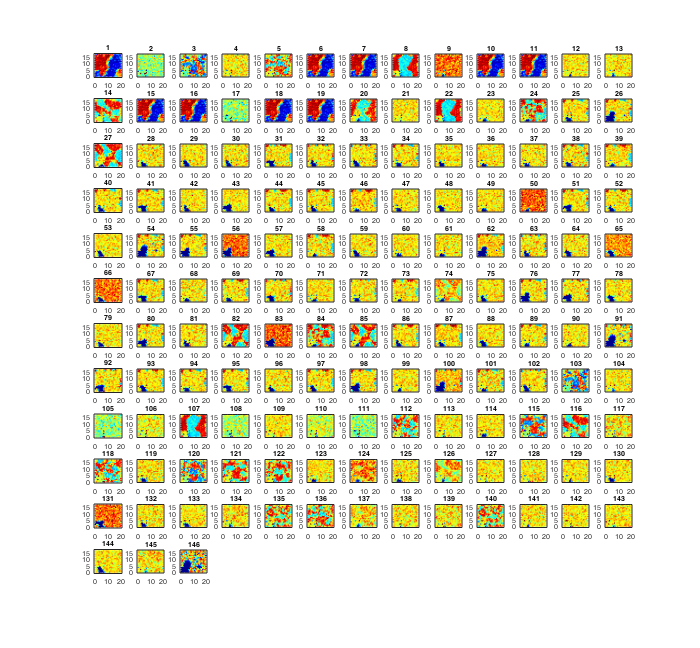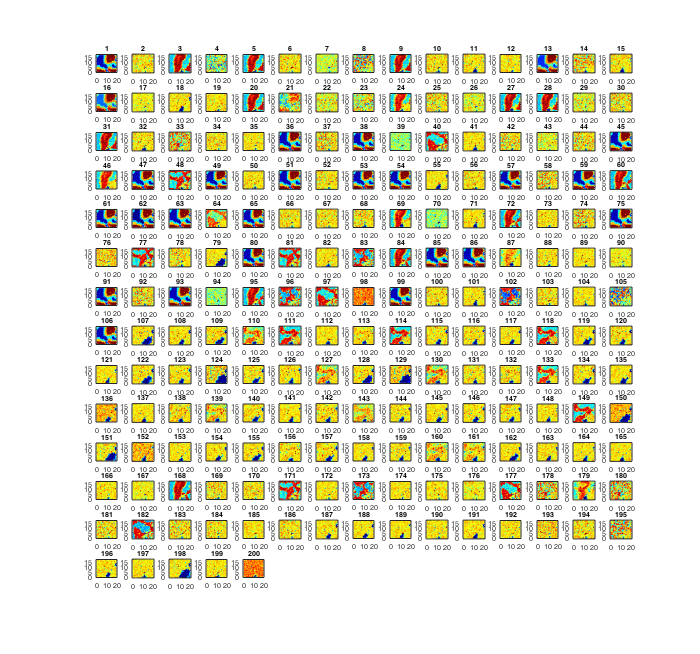  South America:  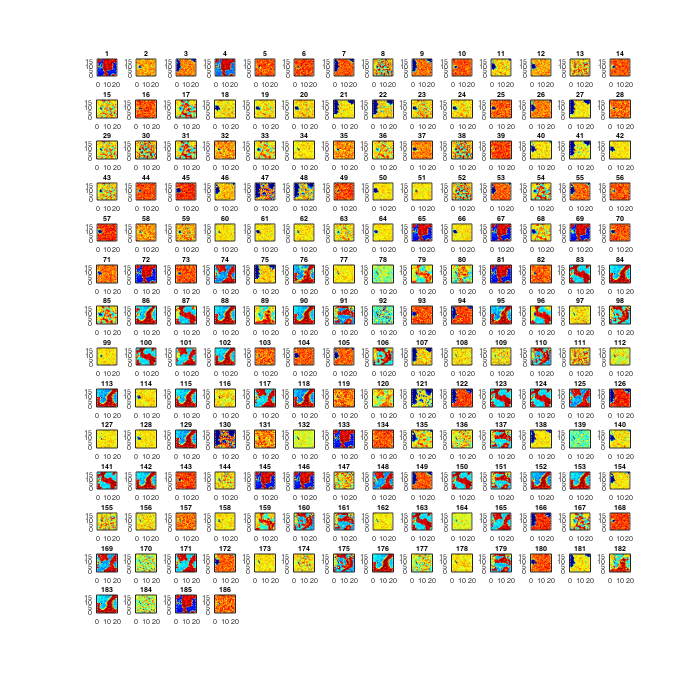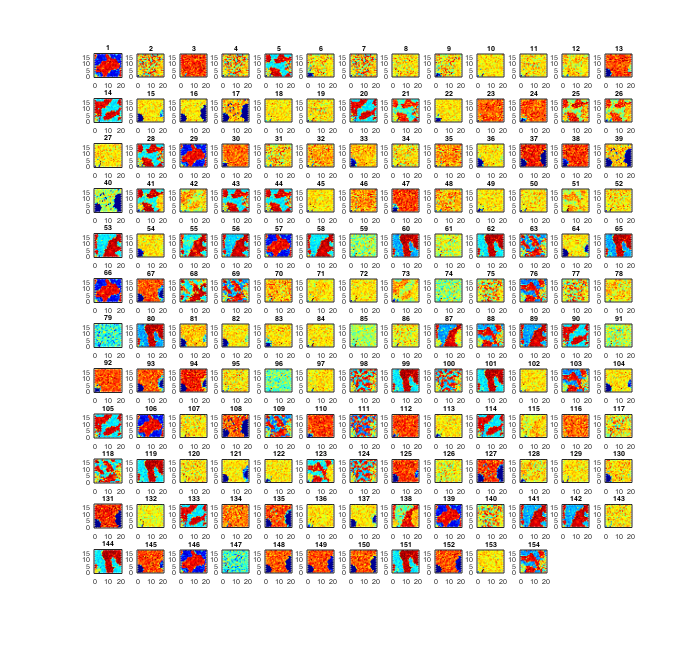  Oceania:   | Male:  ZAF_28, ZAF_29, ZAF_31, ZAF_38, ZAF_41, ZAF_72, ZAF_120, ZAF_293, ZAF_333, ZAF_400, ZAF_419, ZAF_430, TUN_5, TUN_13, CHN_15, CHN_29, CHN_51, LKA_23, BGD_10, BGD_13, BGD_19, IND_229, TWN_3, TWN_9, SAU_15, SAU_71, SAU_106, SAU_123, SAU_126, SAU_196, SAU_200, SAU_207, SAU_250, SAU_305, SAU_322, SAU_335, SAU_349, ARE_55, ARE_58, ARE_61, POL_2, GRC_7, ROU_4, ROU_10, ROU_13, ROU_15, ESP_2, ESP_3, ESP_4, ESP_7, ESP_12, ESP_20, ESP_21, ESP_30, ESP_34, ESP_41, ESP_55, ESP_68, ESP_69, ESP_70, ESP_81, ESP_88, ESP_97, ESP_101, ESP_108, ITA_38, ITA_129, ITA_135, ITA_153, ITA_158, ITA_200, ITA_225, RUS_38, FRA_37, FRA_39, FRA_43, FRA_51, FRA_55, FRA_57, HUN_36, UKR_7, MEX_4, MEX_10, MEX_25, MEX_28, USA_10, USA_38, USA_44, USA_46, USA_111, USA_116, USA_127, USA_140, USA_167, USA_175, USA_176, USA_200, USA_238, USA_251, USA_257, USA_278, USA_282, USA_288, USA_289, USA_298, USA_299, USA_310, USA_311, CHL_1, ECU_4, ECU_8, ECU_17, ECU_20, BRA_9, BRA_113, BRA_142, BRA_144, BRA_256, GUM_1, GUM_2, NZL_1  Female:  ZAF_7, ZAF_46, ZAF_239, ZAF_262, ZAF_303, ZAF_419, ZAF_468, ZAF_475, ZAF_530, ZAF_546, ZAF_615, ZAF_691, ZAF_700, ZAF_829, ZAF_921, ZAF_969, ZAF_1002, CHN_1, CHN_6, CHN_9, CHN_19, CHN_22, CHN_27, CHN_40, CHN_47, CHN_69, CHN_70, CHN_86, CHN_110, CHN_114, KAZ_2, KAZ_7, KAZ_8, KAZ_10, IDN_6, IDN_9, IDN_11, TWN_6, TWN_7, TWN_19, TWN_22, TWN_26, TWN_27, SAU_1, SAU_3, SAU_16, SAU_17, SAU_22, SAU_24, SAU_25, SAU_28, SAU_29, SAU_39, SAU_83, SAU_85, SAU_88, ARE_16, DEU_1, DEU_4, DEU_5, DEU_9, ROU_8, ROU_17, ROU_25, ROU_29, ROU_31, ROU_33, ROU_34, ESP_11, ESP_26, ESP_28, ESP_34, ESP_36, ESP_37, ESP_38, ESP_53, ESP_61, ESP_62, ESP_77, ESP_79, ESP_83, ESP_84, ESP_102, ESP_115, ITA_43, ITA_44, ITA_46, ITA_87, ITA_188, FRA_1, FRA_10, FRA_11, FRA_18, FRA_46, FRA_50, AUT_8, MEX_14, MEX_17, USA_2, USA_5, USA_14, USA_17, USA_20, USA_23, USA_27, USA_28, USA_32, USA_34, USA_43, USA_46, USA_49, USA_57, USA_95, USA_112, USA_132, USA_133, USA_150, USA_169, USA_181, COL_40, BRA_9, BRA_24, BRA_102, BRA_216, BRA_243, NZL_1 |
| 2-G1 inter-continent sub-strain(s) | 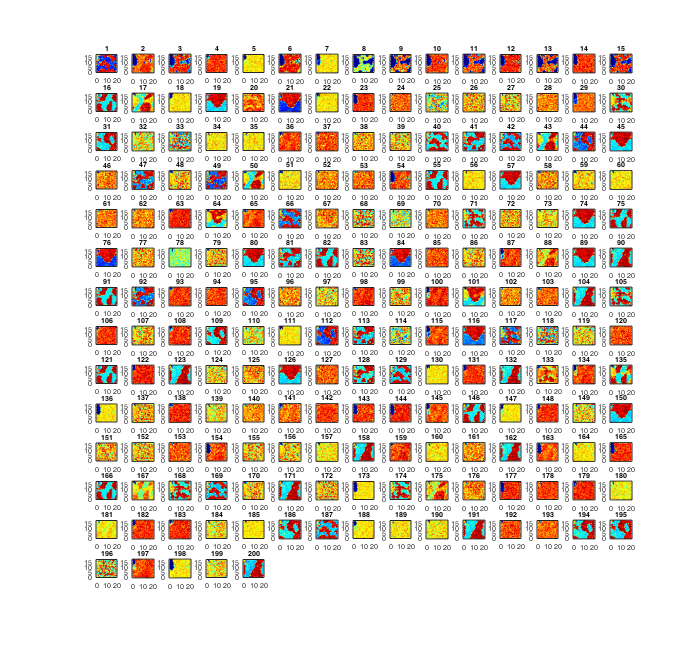 | Male:  GHA_2, GHA_4, GHA_8, GHA_12, ZAF_5, ZAF_9, ZAF_10, ZAF_15, ZAF_16, ZAF_24, ZAF_25, ZAF_36, ZAF_44, ZAF_45, ZAF_46, ZAF_56, ZAF_63, ZAF_64, ZAF_65, ZAF_70, ZAF_76, ZAF_82, ZAF_111, ZAF_114, ZAF_121, ZAF_123, ZAF_125, ZAF_128, ZAF_130, ZAF_133, ZAF_136, ZAF_144, ZAF_149, ZAF_167, ZAF_175, ZAF_178, ZAF_192, ZAF_199, ZAF_204, ZAF_218, ZAF_223, ZAF_231, ZAF_232, ZAF_235, ZAF_236, ZAF_239, ZAF_245, ZAF_255, ZAF_256, ZAF_266, ZAF_286, ZAF_310, ZAF_311, ZAF_312, ZAF_319, ZAF_323, ZAF_340, ZAF_341, ZAF_359, ZAF_366, ZAF_380, ZAF_396, ZAF_417, ZAF_438, ZAF_445, ZAF_451, ZAF_464, ZAF_465, ZAF_476, ZAF_488, ZAF_490, ZAF_501, NGA_2, NGA_3, SEN_12, SGP_104, SGP_211, SGP_239, SGP_276, SGP_410, IRQ_1, CHN_3, CHN_6, CHN_8, CHN_24, CHN_38, CHN_70, CHN_117, CHN_124, CHN_127, CHN_184, KWT_3, MYS_1, MYS_3, MYS_21, MYS_23, MYS_30, MYS_37, MYS_41, LKA_3, LKA_5, LKA_10, LKA_12, LKA_13, LKA_14, LKA_16, LKA_21, LKA_22, BGD_1, BGD_9, IND_8, IND_15, IND_20, IND_52, IND_58, IND_81, IND_101, IND_117, IND_121, IND_129, IND_136, IND_145, IND_189, IND_191, IND_193, IND_195, IND_205, IND_209, IND_214, IND_232, IND_236, IND_252, IND_257, IND_265, IND_270, IND_271, IND_279, IND_324, IND_357, IND_361, IND_362, IND_379, IND_394, IND_420, IND_482, IND_494, IND_515, IND_521, IND_527, IND_534, IND_542, IND_543, IND_544, IND_552, IND_560, IND_595, IND_611, IND_614, IND_624, IND_655, IND_664, IND_678, IND_690, IND_708, IND_717, IND_733, IND_823, IND_830, IND_834, IND_865, IND_879, IND_908, IND_914, IND_932, IND_976, IND_978, IND_1007, IND_1021, IND_1024, IND_1036, IND_1039, South Korea_1, South Korea_7, IDN_5, IDN_48, IDN_50, IDN_52, IDN_57, TUR_1, TUR_12, TUR_17, TUR_32, TUR_47, TUR_79, Iran_3, TWN_23, VNM_1, VNM_4, VNM_22, VNM_29, VNM_30, VNM_34, SAU_121, SAU_360, SAU_385, OMN_24, LBN_2, LBN_4, LBN_11, LBN_12, ARE_57, FRO_1, FRO_7, BEL_5, POL_6, GRC_1, GRC_2, GRC_3, GRC_4, GRC_5, GRC_6, GRC_9, GRC_10, GRC_11, GRC_12, GRC_13, GRC_15, GRC_16, GRC_17, ROU_1, ROU_6, ROU_8, ROU_12, ROU_16, ROU_17, ESP_1, ESP_5, ESP_10, ESP_24, ESP_26, ESP_48, ESP_49, ESP_52, ESP_75, ESP_77, ESP_87, ESP_89, ESP_90, ESP_113, ESP_136, ESP_142, GEO_1, GEO_3, ITA_4, ITA_6, ITA_15, ITA_17, ITA_26, ITA_28, ITA_29, ITA_37, ITA_46, ITA_50, ITA_56, ITA_61, ITA_63, ITA_72, ITA_73, ITA_77, ITA_91, ITA_92, ITA_94, ITA_95, ITA_101, ITA_102, ITA_116, ITA_130, ITA_147, ITA_171, ITA_172, ITA_173, ITA_176, ITA_181, ITA_182, ITA_186, ITA_189, ITA_195, ITA_201, ITA_213, ITA_249, ITA_269, ITA_270, ITA_273, ITA_294, ITA_295, ITA_300, ITA_301, RUS_9, RUS_19, RUS_22, RUS_24, FRA_5, FRA_11, FRA_13, FRA_14, FRA_38, FRA_40, FRA_42, FRA_54, FRA_59, FRA_61, UKR_3, SWE_1, AUT_9, BIH_3, CZE_3, CZE_5, CZE_50, CZE_64, MEX_3, MEX_5, MEX_8, MEX_9, MEX_11, MEX_12, MEX_13, MEX_16, MEX_17, MEX_20, MEX_21, MEX_23, MEX_31, MEX_32, MEX_34, MEX_36, MEX_39, MEX_42, MEX_43, MEX_46, MEX_47, MEX_48, MEX_51, MEX_52, MEX_54, MEX_59, MEX_61, MEX_62, USA_3, USA_9, USA_12, USA_26, USA_27, USA_28, USA_33, USA_40, USA_49, USA_50, USA_66, USA_68, USA_72, USA_81, USA_85, USA_89, USA_106, USA_118, USA_121, USA_123, USA_124, USA_129, USA_133, USA_144, USA_161, USA_163, USA_169, USA_177, USA_187, USA_198, USA_203, USA_205, USA_227, USA_229, USA_234, USA_237, USA_260, USA_261, USA_266, USA_286, PAN_49, PAN_59, PAN_79, PAN_81, PAN_117, CRI_24, VEN_1, VEN_2, ARG_1, COL_1, COL_2, COL_4, COL_5, COL_7, COL_10, COL_12, COL_28, COL_29, COL_31, COL_36, COL_44, COL_48, COL_51, COL_60, COL_62, COL_73, COL_77, COL_91, COL_94, COL_96, ECU_18, ECU_19, PER_1, BRA_10, BRA_29, BRA_32, BRA_53, BRA_55, BRA_56, BRA_81, BRA_89, BRA_102, BRA_108, BRA_114, BRA_137, BRA_149, BRA_176, BRA_209, BRA_211, BRA_220, BRA_237, BRA_257  Female:  ZAF_5, ZAF_9, ZAF_13, ZAF_25, ZAF_26, ZAF_31, ZAF_38, ZAF_39, ZAF_43, ZAF_45, ZAF_61, ZAF_64, ZAF_71, ZAF_77, ZAF_86, ZAF_98, ZAF_102, ZAF_107, ZAF_108, ZAF_109, ZAF_113, ZAF_120, ZAF_126, ZAF_128, ZAF_130, ZAF_144, ZAF_176, ZAF_186, ZAF_203, ZAF_223, ZAF_237, ZAF_254, ZAF_255, ZAF_256, ZAF_271, ZAF_274, ZAF_278, ZAF_292, ZAF_307, ZAF_308, ZAF_311, ZAF_312, ZAF_330, ZAF_331, ZAF_335, ZAF_353, ZAF_359, ZAF_360, ZAF_375, ZAF_376, ZAF_377, ZAF_379, ZAF_390, ZAF_391, ZAF_398, ZAF_403, ZAF_412, ZAF_426, ZAF_445, ZAF_455, ZAF_457, ZAF_459, ZAF_467, ZAF_471, ZAF_479, ZAF_482, ZAF_489, ZAF_491, ZAF_502, ZAF_508, ZAF_518, ZAF_523, ZAF_536, ZAF_568, ZAF_586, ZAF_595, ZAF_601, ZAF_607, ZAF_646, ZAF_682, ZAF_688, ZAF_694, ZAF_713, ZAF_733, ZAF_741, ZAF_753, ZAF_761, ZAF_769, ZAF_786, ZAF_798, ZAF_815, ZAF_820, ZAF_828, ZAF_830, ZAF_874, ZAF_879, ZAF_903, ZAF_906, ZAF_912, ZAF_917, ZAF_928, ZAF_936, ZAF_937, ZAF_950, EGY_11, EGY_12, NGA_2, SEN_9, SEN_46, SEN_49, SEN_52, SGP_1, SGP_2, SGP_3, SGP_25, IRQ_1, CHN_12, CHN_14, CHN_30, CHN_34, CHN_52, CHN_54, CHN_64, CHN_75, CHN_80, CHN_101, CHN_115, CHN_123, MYS_6, MYS_17, MYS_28, MYS_30, LKA_2, LKA_3, LKA_4, IND_11, IND_12, IND_17, IND_26, IND_29, IND_31, IND_32, IND_49, IND_57, IND_70, IND_80, IND_85, IND_95, IND_103, IND_106, IND_124, IND_131, IND_135, IND_144, IND_154, IND_157, IND_158, IND_173, IND_177, IND_180, IND_182, IND_186, IND_200, IND_201, IND_205, IND_212, IND_233, IND_240, IND_251, IND_254, IND_255, IND_267, IND_279, IND_289, IND_299, IND_305, IND_311, IND_313, IND_336, IND_339, IND_341, IND_350, IND_355, IND_359, IND_361, IND_363, IND_364, IND_379, IND_385, IND_395, IND_403, IND_422, IND_423, IND_428, IND_433, IND_434, IND_439, IND_462, IND_471, IND_475, IND_491, IND_493, IND_500, IND_503, IND_509, IND_516, IND_534, IND_540, IDN_2, IDN_8, TUR_15, TUR_35, TUR_50, Iran_1, Iran_2, TWN_14, TWN_18, VNM_6, VNM_10, VNM_11, VNM_13, VNM_14, VNM_26, VNM_32, VNM_33, ISR_13, SAU_23, PAK_2, LBN_3, LBN_6, GRC_2, GRC_4, GRC_5, GRC_6, GRC_7, GRC_9, GRC_12, DEU_2, ROU_1, ROU_3, ROU_20, ROU_22, ESP_9, ESP_15, ESP_25, ESP_45, ESP_46, ESP_51, ESP_63, ESP_70, ITA_9, ITA_11, ITA_17, ITA_29, ITA_32, ITA_68, ITA_71, ITA_72, ITA_96, ITA_97, ITA_102, ITA_108, ITA_114, ITA_115, ITA_116, ITA_137, ITA_145, ITA_161, ITA_167, ITA_170, ITA_174, ITA_189, ITA_199, ITA_206, ITA_231, ITA_235, ITA_242, ITA_244, ITA_245, ITA_252, RUS_1, RUS_3, RUS_32, RUS_41, RUS_73, RUS_78, FRA_2, FRA_4, FRA_7, FRA_12, FRA_14, FRA_19, FRA_21, FRA_28, FRA_38, FRA_39, FRA_41, FRA_43, FRA_47, SVK_3, MDA_1, MDA_2, MDA_3, UKR_4, FIN_2, CZE_52, CZE_58, USA_159, PAN_51, PAN_77, PAN_79, DOM_5, ARG_1, COL_14, COL_32, COL_33, COL_41, COL_47, COL_48, COL_52, COL_57, COL_75, COL_77, BRA_25, BRA_75, BRA_77, BRA_78, BRA_107, BRA_115, BRA_120, BRA_142, BRA_175, BRA_182, BRA_190, BRA_196, BRA_200, BRA_242, BRA_246, BRA_247, BRA_254, BRA_258 |
| 3-G2 inter-continent sub-strain(s) | 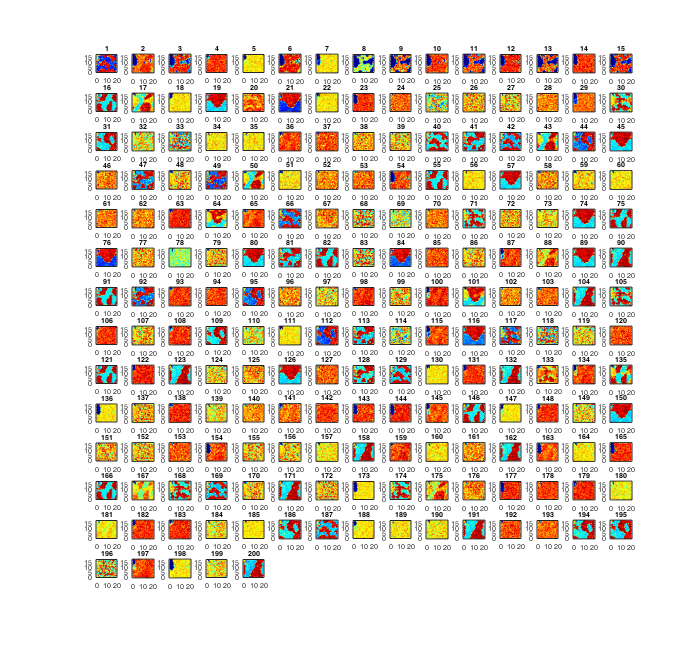 | Male:  GHA_3, GHA_5, ZAF_3, ZAF_8, ZAF_21, ZAF_23, ZAF_43, ZAF_51, ZAF_57, ZAF_60, ZAF_85, ZAF_96, ZAF_98, ZAF_112, ZAF_126, ZAF_161, ZAF_198, ZAF_213, ZAF_244, ZAF_263, ZAF_269, ZAF_284, ZAF_301, ZAF_313, ZAF_316, ZAF_328, ZAF_329, ZAF_334, ZAF_336, ZAF_337, ZAF_362, ZAF_363, ZAF_367, ZAF_377, ZAF_387, ZAF_393, ZAF_402, ZAF_408, ZAF_433, ZAF_444, ZAF_446, ZAF_457, ZAF_467, ZAF_470, ZAF_479, GMB_2, GMB_6, GMB_7, EGY_1, EGY_3, EGY_13, EGY_16, EGY_19, EGY_21, MOZ_1, MOZ_3, NGA_1, NGA_4, NGA_6, NGA_7, NGA_8, NGA_9, NGA_11, NGA_12, SEN_7, SEN_16, SEN_17, SEN_18, SEN_25, SEN_27, SEN_30, SEN_32, SEN_35, SEN_36, SEN_39, SEN_40, SEN_44, SEN_47, SEN_50, SEN_52, SEN_58, SEN_61, SEN_62, SEN_63, SEN_65, SEN_71, CHN_5, CHN_19, CHN_35, CHN_52, CHN_74, CHN_79, CHN_90, CHN_104, CHN_108, CHN_150, CHN_154, CHN_157, CHN_168, CHN_183, KWT_1, MYS_8, MYS_14, MYS_17, MYS_34, LKA_11, LKA_17, LKA_18, BGD_14, IND_1, IND_17, IND_22, IND_25, IND_27, IND_42, IND_56, IND_67, IND_93, IND_94, IND_124, IND_162, IND_173, IND_190, IND_199, IND_206, IND_208, IND_274, IND_300, IND_315, IND_346, IND_352, IND_354, IND_377, IND_442, IND_453, IND_458, IND_508, IND_545, IND_558, IND_572, IND_598, IND_616, IND_625, IND_651, IND_716, IND_724, IND_746, IND_767, IND_816, IND_890, IND_892, IND_933, IND_946, IND_960, IND_962, IND_1003, IND_1010, TUR_61, Iran_1, Iran_2, TWN_10, TWN_18, SAU_115, SAU_378, MNG_1, MNG_2, MNG_3, OMN_22, LBN_5, LBN_7, LBN_8, LBN_9, ARE_21, ESP_6, ESP_72, ESP_98, ITA_1, ITA_10, ITA_142, ITA_149, ITA_160, FRA_49, FRA_72, CYP_1, UKR_2, HRV_6, MEX_19, MEX_33, MEX_41, MEX_56, MEX_57, USA_244, USA_276, USA_290, USA_294, USA_314, USA_318, PAN_2, PAN_3, PAN_7, PAN_8, PAN_9, PAN_10, PAN_14, PAN_16, PAN_17, PAN_19, PAN_23, PAN_24, PAN_25, PAN_29, PAN_31, PAN_32, PAN_33, PAN_34, PAN_36, PAN_38, PAN_39, PAN_45, PAN_46, PAN_47, PAN_51, PAN_53, PAN_55, PAN_57, PAN_58, PAN_62, PAN_64, PAN_65, PAN_67, PAN_68, PAN_70, PAN_71, PAN_73, PAN_86, PAN_87, PAN_91, PAN_93, PAN_94, PAN_96, PAN_99, PAN_102, PAN_105, PAN_107, PAN_108, PAN_112, PAN_113, PAN_114, PAN_124, PAN_125, PAN_128, PAN_130, PAN_131, PAN_133, PAN_134, PAN_136, PAN_137, PAN_140, PAN_141, PAN_144, PAN_145, PAN_146, PAN_147, PAN_148, Saint Martin_3, GLP_4, GLP_7, CRI_1, CRI_2, CRI_7, CRI_9, CRI_15, CRI_17, CRI_19, CRI_20, CRI_22, CRI_23, CRI_25, CRI_26, CRI_27, CRI_30, CRI_32, CRI_33, CRI_35, CRI_36, CRI_37, CRI_38, CRI_39, COL_14, COL_15, COL_18, COL_22, COL_24, COL_25, COL_27, COL_30, COL_43, COL_52, COL_53, COL_55, COL_65, COL_74, COL_75, COL_106, COL_107, COL_108, ECU_2, ECU_6, BRA_3, BRA_5, BRA_40, BRA_46, BRA_59, BRA_64, BRA_65, BRA_69, BRA_73, BRA_75, BRA_103, BRA_104, BRA_123, BRA_126, BRA_139, BRA_170, BRA_174, BRA_179, BRA_191, BRA_225, BRA_226, BRA_235, BRA_241  Female:  ZAF_3, ZAF_33, ZAF_37, ZAF_95, ZAF_99, ZAF_129, ZAF_143, ZAF_154, ZAF_166, ZAF_187, ZAF_208, ZAF_213, ZAF_218, ZAF_225, ZAF_285, ZAF_294, ZAF_318, ZAF_333, ZAF_352, ZAF_395, ZAF_449, ZAF_476, ZAF_511, ZAF_524, ZAF_531, ZAF_549, ZAF_571, ZAF_577, ZAF_580, ZAF_590, ZAF_599, ZAF_608, ZAF_613, ZAF_621, ZAF_622, ZAF_642, ZAF_645, ZAF_648, ZAF_656, ZAF_659, ZAF_664, ZAF_667, ZAF_673, ZAF_675, ZAF_698, ZAF_705, ZAF_710, ZAF_714, ZAF_721, ZAF_740, ZAF_770, ZAF_779, ZAF_780, ZAF_789, ZAF_795, ZAF_805, ZAF_810, ZAF_826, ZAF_844, ZAF_854, ZAF_857, ZAF_870, ZAF_885, ZAF_896, ZAF_900, ZAF_908, ZAF_910, ZAF_923, ZAF_925, ZAF_944, ZAF_947, ZAF_978, ZAF_980, ZAF_984, ZAF_993, ZAF_995, GMB_2, EGY_7, EGY_9, EGY_10, EGY_14, TUN_13, MDG_1, NGA_1, NGA_4, SEN_2, SEN_5, SEN_13, SEN_15, SEN_24, SEN_28, SEN_29, SEN_30, SEN_31, SEN_33, SEN_47, SEN_48, SEN_53, RWA_1, CHN_38, CHN_45, CHN_98, CHN_106, CHN_128, MYS_1, MYS_20, MYS_32, MYS_33, MYS_35, MYS_38, MYS_45, MYS_46, LKA_5, IND_9, IND_18, IND_35, IND_45, IND_62, IND_78, IND_79, IND_86, IND_88, IND_90, IND_96, IND_97, IND_104, IND_121, IND_129, IND_132, IND_145, IND_155, IND_164, IND_167, IND_170, IND_209, IND_217, IND_250, IND_253, IND_257, IND_272, IND_278, IND_332, IND_338, IND_345, IND_351, IND_354, IND_356, IND_358, IND_370, IND_377, IND_409, IND_414, IND_425, IND_429, IND_435, IND_456, IND_488, IND_505, IND_517, IND_529, IND_531, South Korea_4, IDN_18, IDN_25, TUR_2, TUR_37, TUR_45, TWN_21, VNM_4, ISR_2, ISR_8, PHL_1, SAU_13, SAU_79, SAU_84, OMN_5, LBN_1, LBN_2, LBN_5, BHR_1, GRC_13, ROU_11, ESP_1, ESP_3, ESP_8, ESP_18, ESP_42, ESP_58, ESP_60, ESP_69, ESP_82, ESP_87, ESP_101, ESP_108, ESP_111, ITA_1, ITA_6, ITA_26, ITA_45, ITA_54, ITA_109, ITA_127, ITA_129, ITA_152, ITA_164, ITA_168, ITA_176, ITA_191, ITA_237, ITA_239, FRA_53, UKR_2, MEX_1, MEX_5, MEX_9, MEX_11, MEX_13, MEX_15, MEX_16, MEX_18, MEX_19, MEX_20, MEX_31, MEX_39, MEX_41, MEX_43, USA_1, USA_3, USA_9, USA_11, USA_13, USA_21, USA_26, USA_29, USA_31, USA_58, USA_66, USA_67, USA_80, USA_93, USA_94, USA_97, USA_107, USA_108, USA_124, USA_135, USA_137, USA_138, USA_144, USA_171, USA_173, USA_175, USA_178, PAN_1, PAN_2, PAN_3, PAN_6, PAN_7, PAN_9, PAN_10, PAN_15, PAN_19, PAN_21, PAN_22, PAN_24, PAN_27, PAN_29, PAN_30, PAN_31, PAN_34, PAN_35, PAN_40, PAN_42, PAN_44, PAN_46, PAN_54, PAN_56, PAN_59, PAN_61, PAN_62, PAN_64, PAN_67, PAN_69, PAN_71, PAN_73, PAN_78, PAN_80, PAN_81, PAN_83, PAN_84, PAN_85, PAN_86, PAN_88, PAN_92, PAN_98, PAN_101, PAN_102, Saint Martin_1, Saint Martin_2, GLP_1, GLP_2, GLP_5, GLP_7, GLP_9, GLP_10, CAN_10, CRI_2, CRI_3, CRI_4, CRI_5, CRI_6, CRI_7, CRI_9, CRI_10, CRI_11, CRI_12, CRI_13, CRI_14, CRI_17, DOM_1, DOM_2, DOM_4, COL_3, COL_5, COL_7, COL_8, COL_13, COL_19, COL_20, COL_24, COL_26, COL_30, COL_38, COL_44, COL_49, COL_55, COL_64, COL_72, ECU_2, ECU_4, BRA_3, BRA_5, BRA_19, BRA_33, BRA_35, BRA_39, BRA_50, BRA_54, BRA_55, BRA_58, BRA_61, BRA_65, BRA_79, BRA_82, BRA_91, BRA_99, BRA_103, BRA_130, BRA_140, BRA_151, BRA_155, BRA_159, BRA_178, BRA_185, BRA_186, BRA_188, BRA_202, BRA_211, BRA_233, BRA_259 |
| 4-G3 inter-country sub-strain(s) | 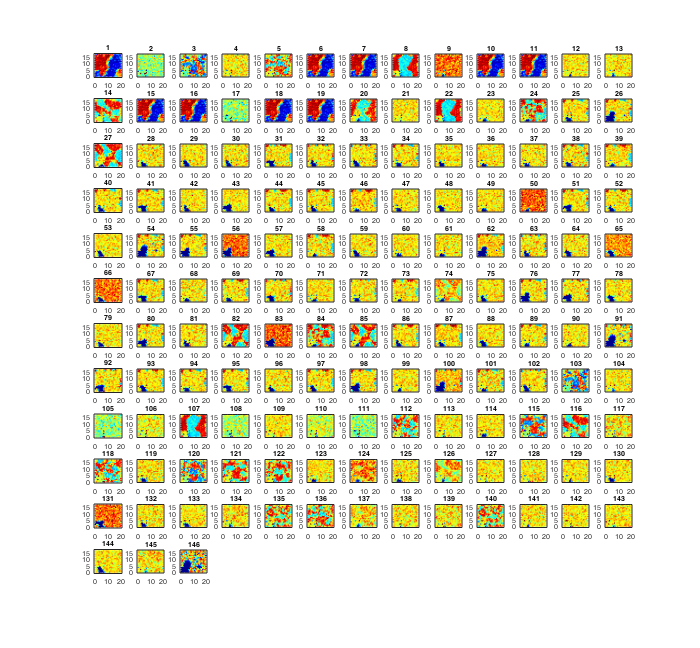 | Male:  ZAF_12, ZAF_78, ZAF_97, ZAF_151, ZAF_379, TUN_3, TUN_4, TUN_9, TUN_11, TUN_12, CHN_135, MYS_31, MYS_32, LKA_15, BGD_4, BGD_8, BGD_11, TUR_24, GRC_8, ITA_155, ITA_183, ITA_207, ITA_226, ITA_292, FRA_18, FRA_32, USA_48, USA_267, USA_301, Saint Martin_4, GLP_6, CAN_8, CAN_10, BRA_6, BRA_24, BRA_111, BRA_125, BRA_194, BRA_217, BRA_231, BRA_253  Female:  ZAF_20, ZAF_257, ZAF_526, ZAF_922, TUN_4, TUN_5, TUN_8, TUN_10, TUN_11, RWA_2, CHN_16, CHN_39, CHN_53, MYS_5, MYS_12, MYS_15, BGD_7, IND_10, IND_42, IND_161, IND_163, IND_197, IND_198, IND_214, IND_237, IND_275, KAZ_5, TWN_3, TWN_8, GRC_11, ROU_14, ITA_52, ITA_70, ITA_134, ITA_247, RUS_7, RUS_45, FRA_23, MEX_4, MEX_6, MEX_27, MEX_37, USA_7, USA_12, USA_77, USA_151, COL_65, BRA_11, BRA_15, BRA_43, BRA_62, BRA_74, BRA_80 |
| 5-G4 inter-continent sub-strain(s) | 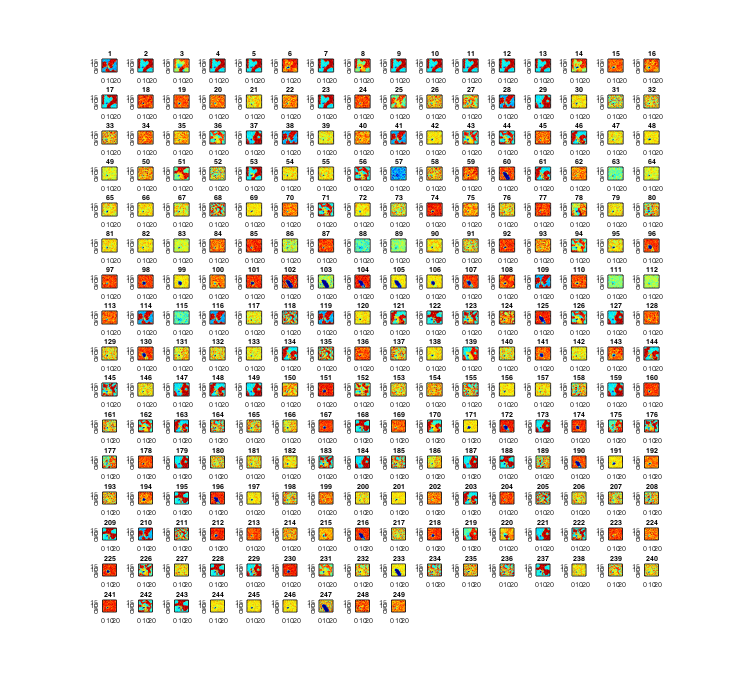 | Male:  CHN_115, SAU_381, ITA_235, ITA_289  Female:  PHL_6, ESP_14, ITA_141, ITA_225, ITA_233, RUS_14, RUS_16, RUS_17, FRA_52, UKR_1, UKR_5, BRA_51, BRA_244 |
| 6-G5 inter-continent sub-strain(s) | 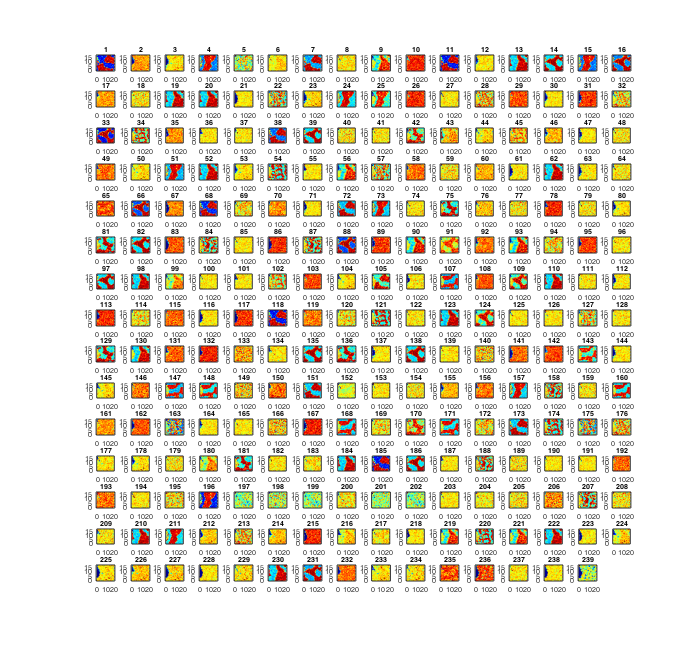 | Male:  ZAF_14, ZAF_19, ZAF_20, ZAF_26, ZAF_27, ZAF_77, ZAF_106, ZAF_173, ZAF_214, ZAF_246, ZAF_252, ZAF_253, ZAF_257, ZAF_259, ZAF_372, ZAF_391, ZAF_439, ZAF_440, ZAF_441, ZAF_482, ZAF_487, ZAF_493, DZA_1, SEN_42, SEN_77, CHN_13, CHN_14, CHN_20, CHN_22, CHN_23, CHN_99, CHN_116, CHN_182, MYS_9, MYS_35, BGD_17, IND_31, IND_40, IND_86, IND_91, IND_95, IND_103, IND_112, IND_115, IND_153, IND_154, IND_170, IND_184, IND_192, IND_204, IND_218, IND_219, IND_223, IND_224, IND_231, IND_304, IND_306, IND_312, IND_317, IND_356, IND_381, IND_395, IND_406, IND_425, IND_499, IND_502, IND_514, IND_536, IND_563, IND_564, IND_576, IND_579, IND_584, IND_593, IND_623, IND_641, IND_680, IND_711, IND_760, IND_763, IND_787, IND_801, IND_838, IND_846, IND_861, IND_867, IND_956, IND_984, IND_997, South Korea_3, South Korea_8, KAZ_1, IDN_9, IDN_25, IDN_26, IDN_35, TUR_7, Iran_5, TWN_4, TWN_12, VNM_6, VNM_15, SAU_273, OMN_3, OMN_5, OMN_17, OMN_19, CHE_1, ROU_14, ESP_11, ESP_37, ESP_73, ESP_83, ESP_95, ITA_5, ITA_18, ITA_23, ITA_48, ITA_62, ITA_98, ITA_118, ITA_120, ITA_121, ITA_216, ITA_227, ITA_281, UKR_5, MEX_2, MEX_44, USA_11, USA_14, USA_18, USA_80, USA_90, USA_114, USA_164, USA_181, USA_274, USA_277, PAN_1, CRI_10, CRI_28, CRI_29, CRI_34, DOM_1, COL_6, COL_8, BRA_67, BRA_118, BRA_119, BRA_173, BRA_210  Female:  ZAF_1, ZAF_4, ZAF_6, ZAF_12, ZAF_28, ZAF_30, ZAF_49, ZAF_54, ZAF_76, ZAF_79, ZAF_88, ZAF_96, ZAF_118, ZAF_138, ZAF_146, ZAF_224, ZAF_232, ZAF_242, ZAF_243, ZAF_260, ZAF_269, ZAF_298, ZAF_304, ZAF_306, ZAF_339, ZAF_351, ZAF_355, ZAF_378, ZAF_380, ZAF_421, ZAF_439, ZAF_443, ZAF_444, ZAF_452, ZAF_480, ZAF_519, ZAF_578, ZAF_594, ZAF_602, ZAF_604, ZAF_635, ZAF_641, ZAF_657, ZAF_661, ZAF_738, ZAF_747, ZAF_782, ZAF_843, ZAF_860, ZAF_902, ZAF_916, ZAF_933, ZAF_941, ZAF_948, ZAF_957, ZAF_962, ZAF_971, ZAF_975, ZAF_977, DZA_1, TUN_1, NGA_3, SEN_6, SEN_17, CHN_5, CHN_18, CHN_20, CHN_43, CHN_50, CHN_71, CHN_88, CHN_122, MYS_4, MYS_7, MYS_8, MYS_18, MYS_24, MYS_31, MYS_47, LKA_1, BGD_4, IND_2, IND_7, IND_33, IND_36, IND_37, IND_46, IND_48, IND_72, IND_74, IND_81, IND_82, IND_100, IND_101, IND_107, IND_108, IND_113, IND_146, IND_148, IND_156, IND_184, IND_187, IND_190, IND_228, IND_274, IND_284, IND_296, IND_301, IND_302, IND_314, IND_337, IND_366, IND_376, IND_387, IND_411, IND_436, IND_446, IND_476, IND_481, IND_483, IND_484, IND_520, IND_532, IND_538, IDN_4, IDN_19, IDN_20, VNM_2, VNM_7, VNM_22, ISR_12, SAU_33, CHE_1, FRO_1, GRC_3, DEU_10, ROU_10, ROU_13, ITA_2, ITA_23, MEX_7, MEX_28, MEX_42, USA_33, USA_179, CAN_3, CAN_7, CRI_15, VEN_1, COL_1, COL_4, COL_6, COL_21, COL_58, BRA_44, BRA_49, BRA_83, BRA_85, BRA_118, BRA_168, BRA_217 |
| 7-Intra-continent sub-strains | Africa:  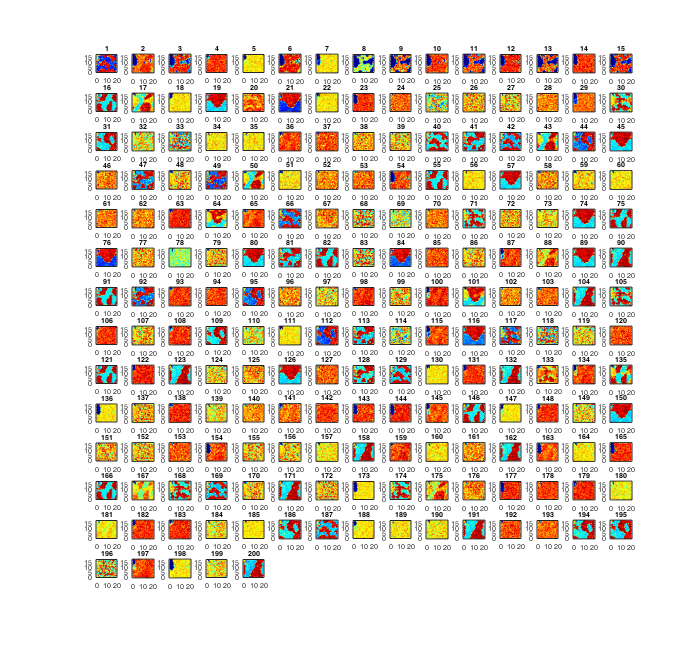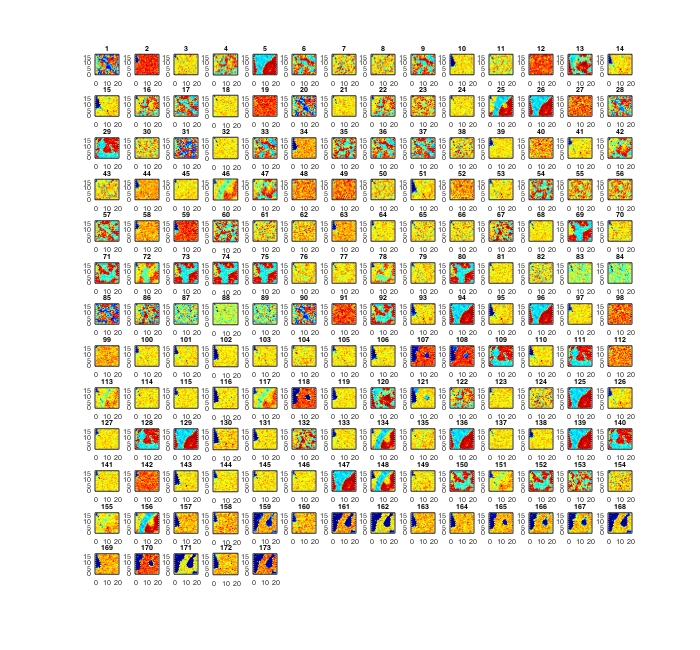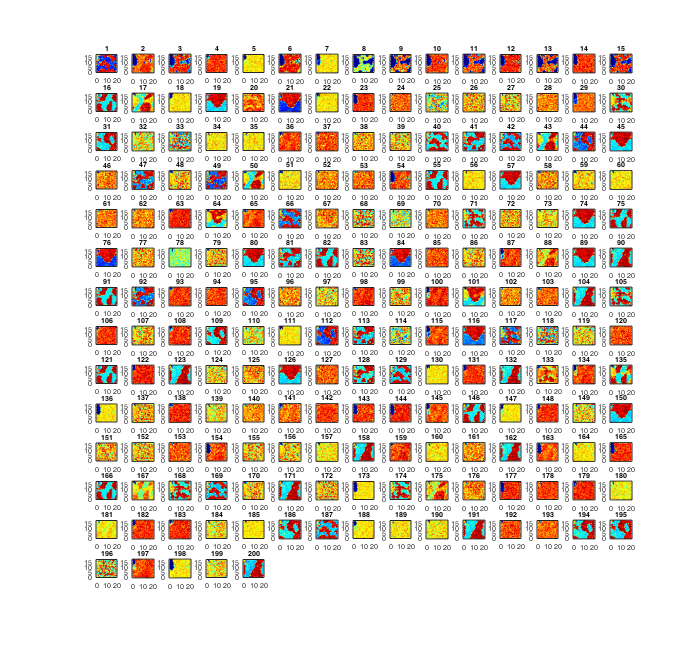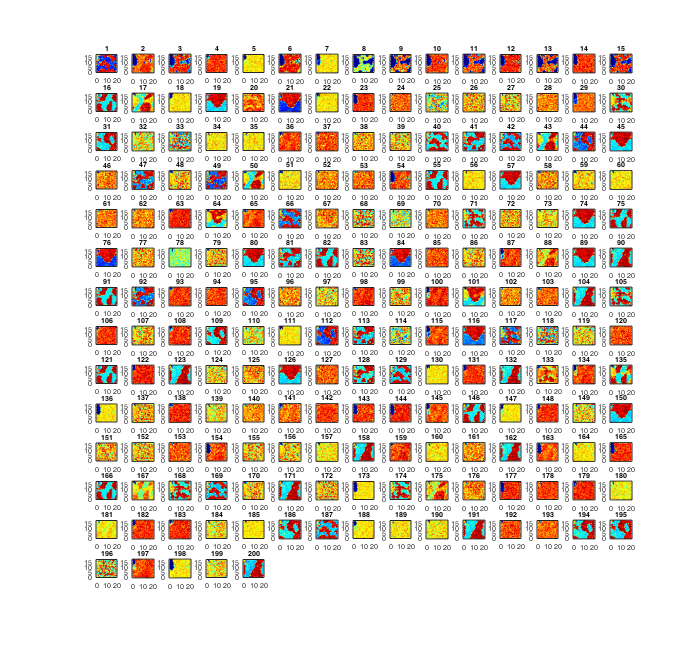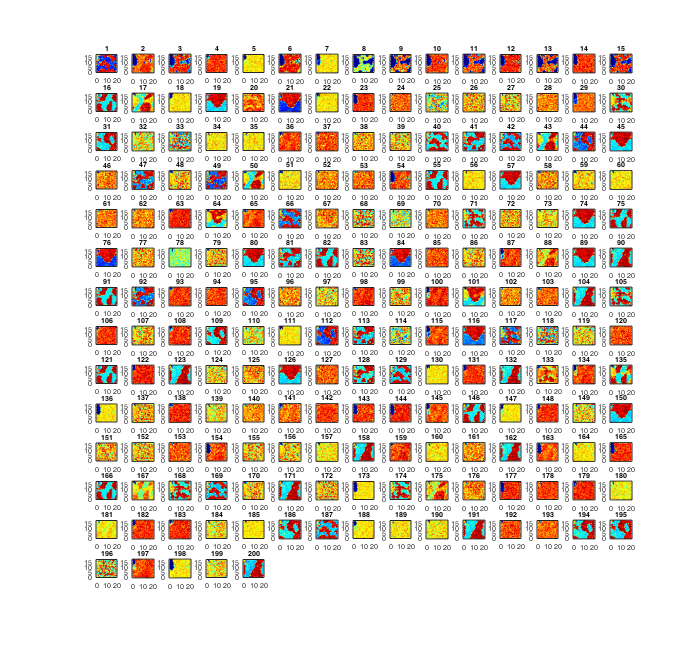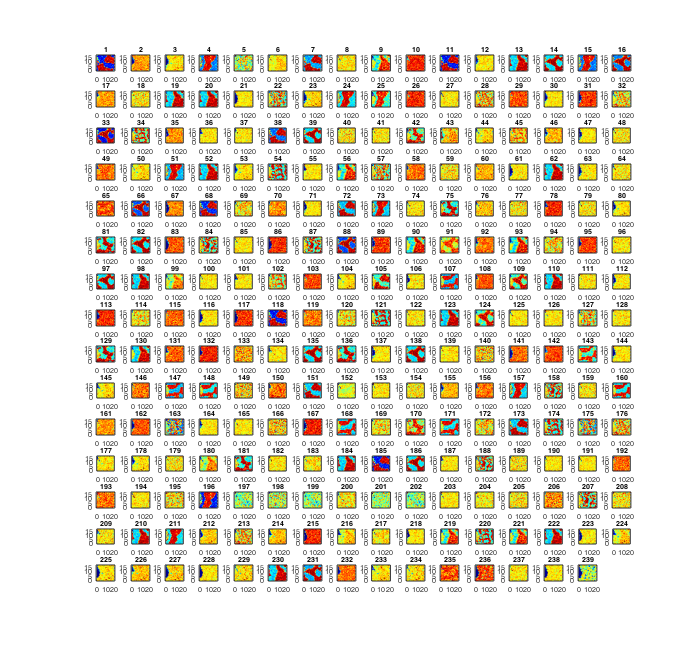  Asia:  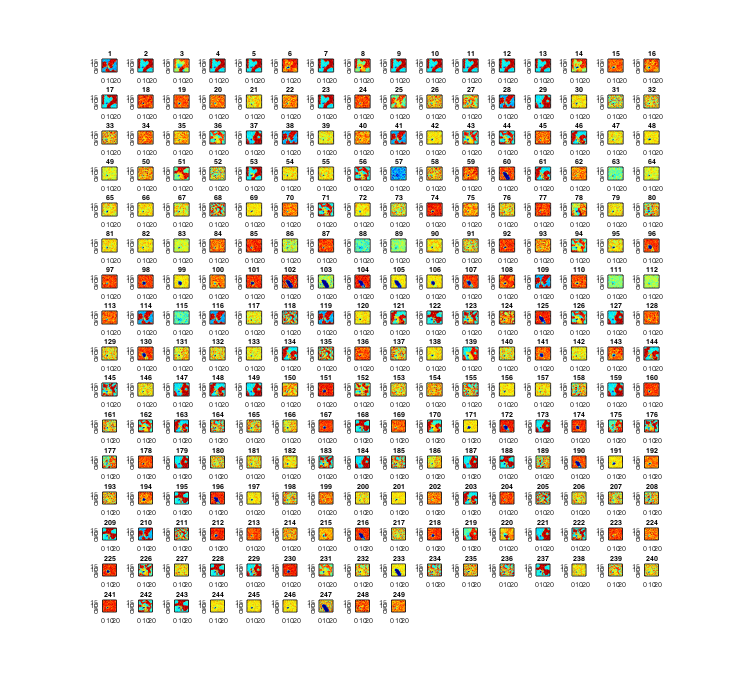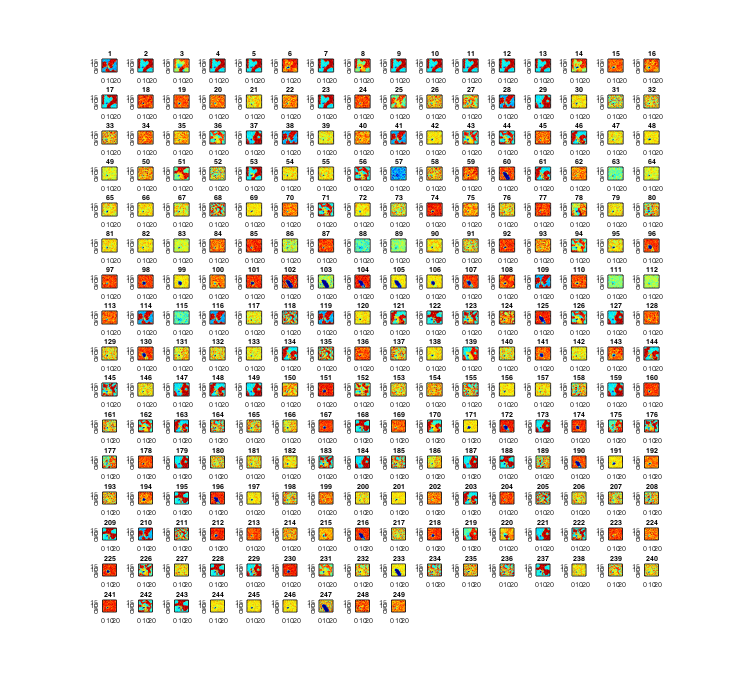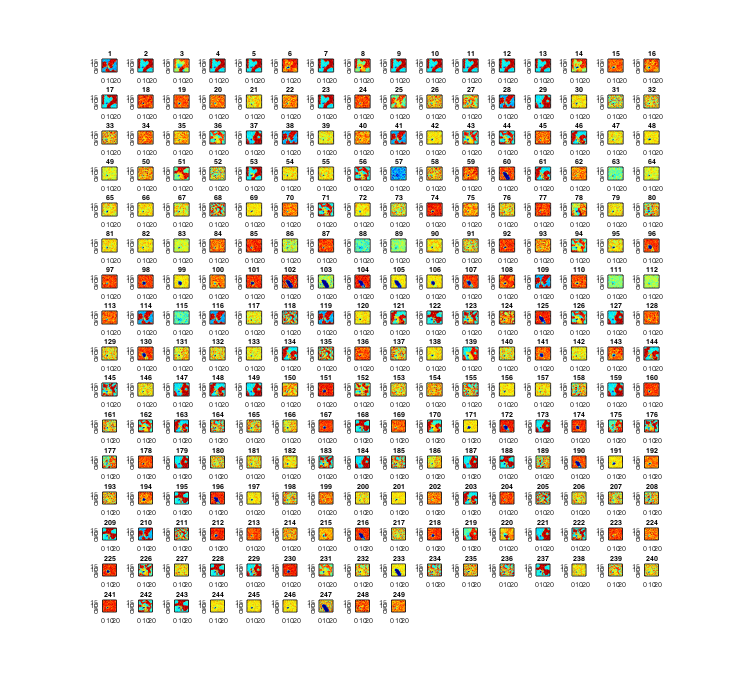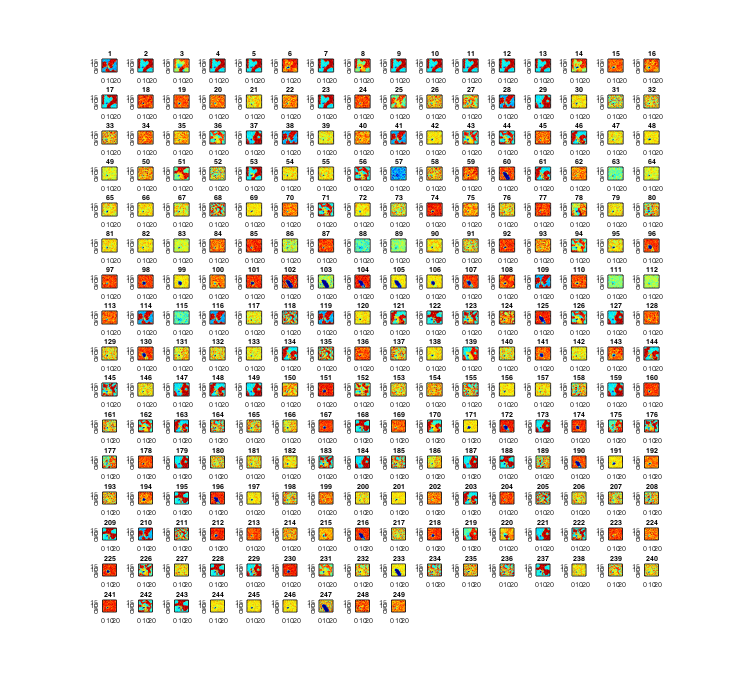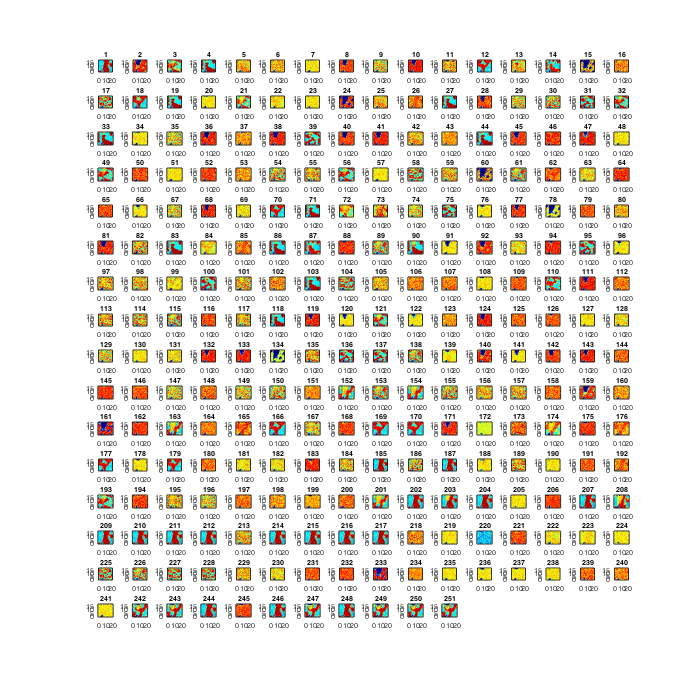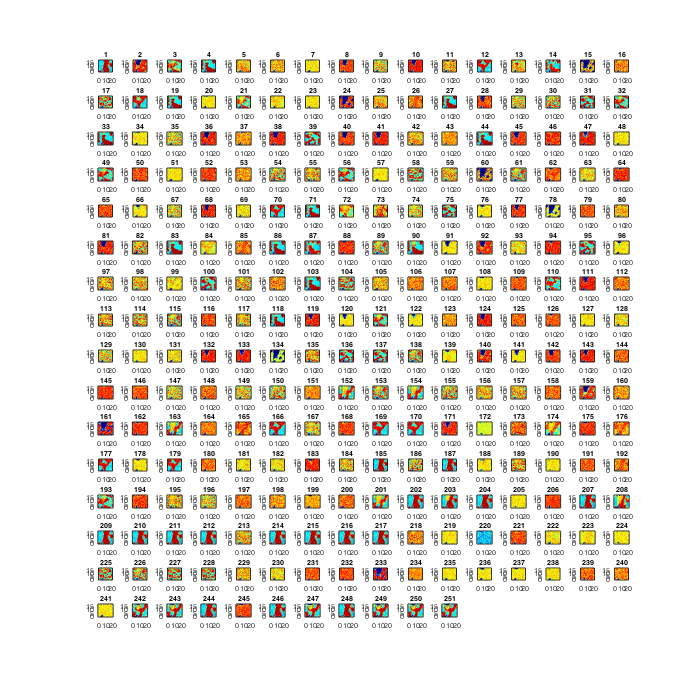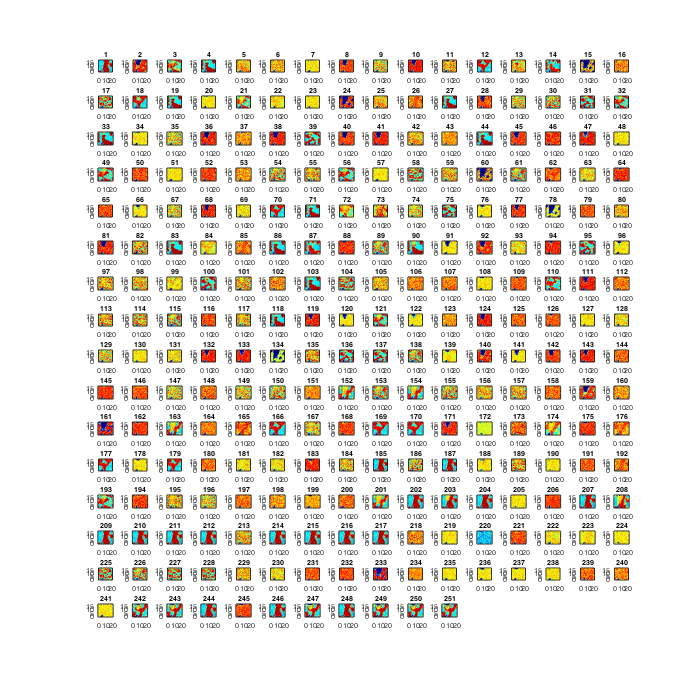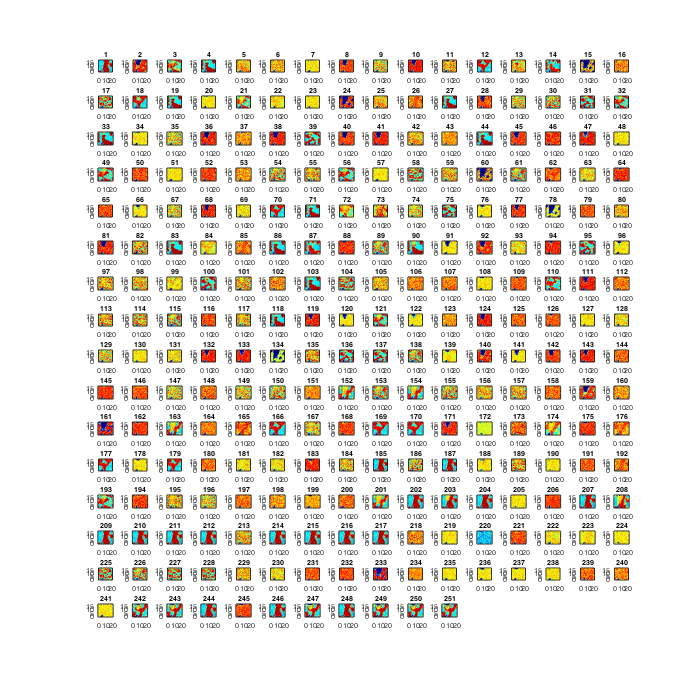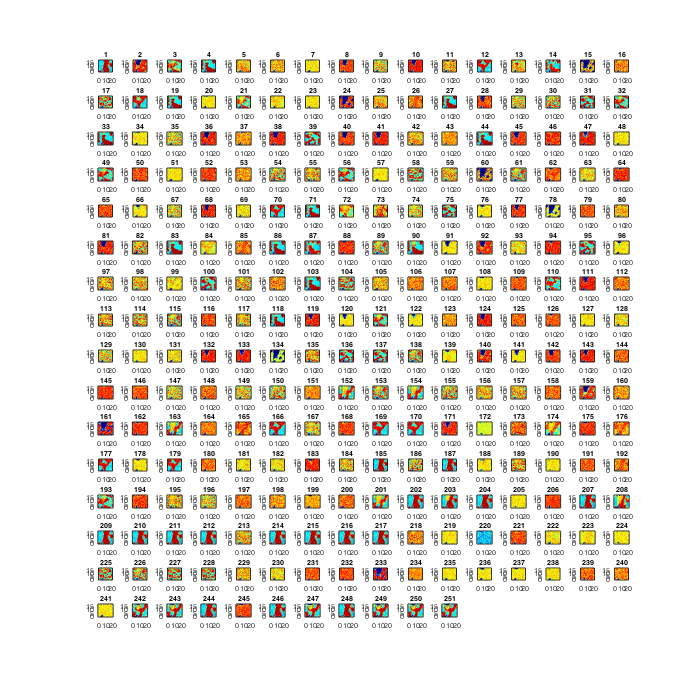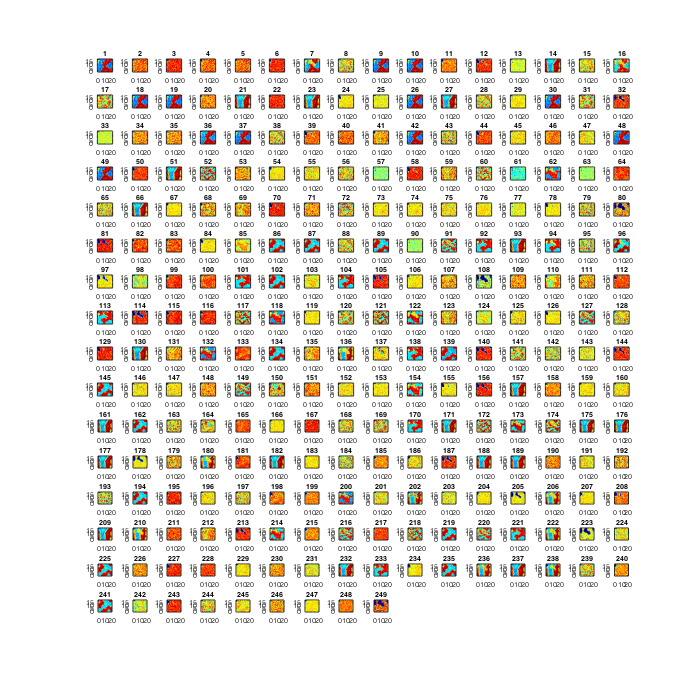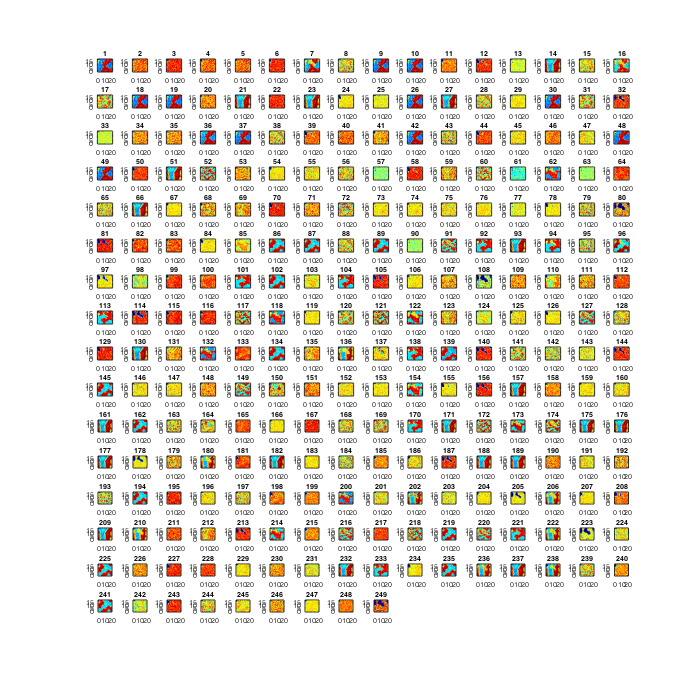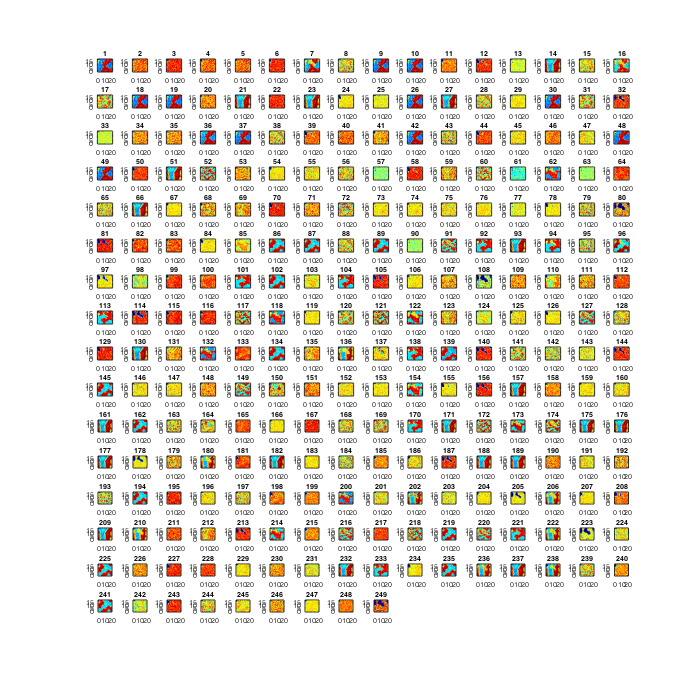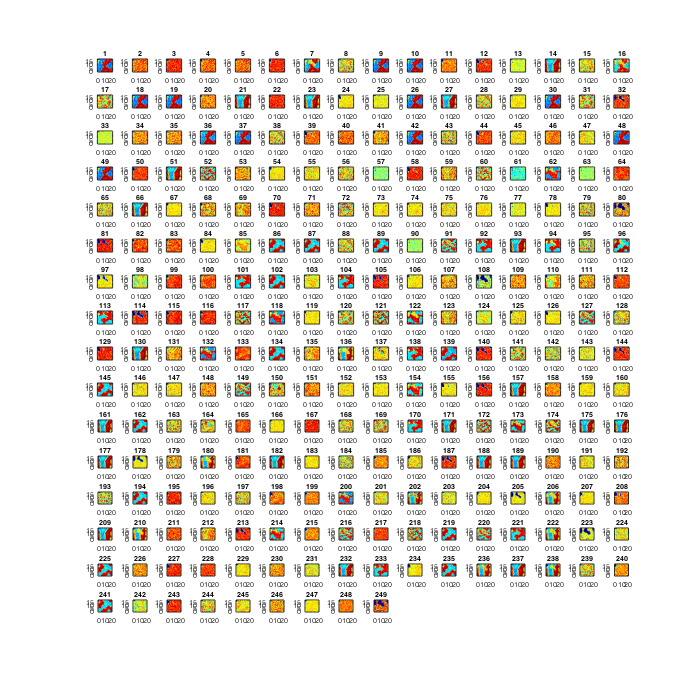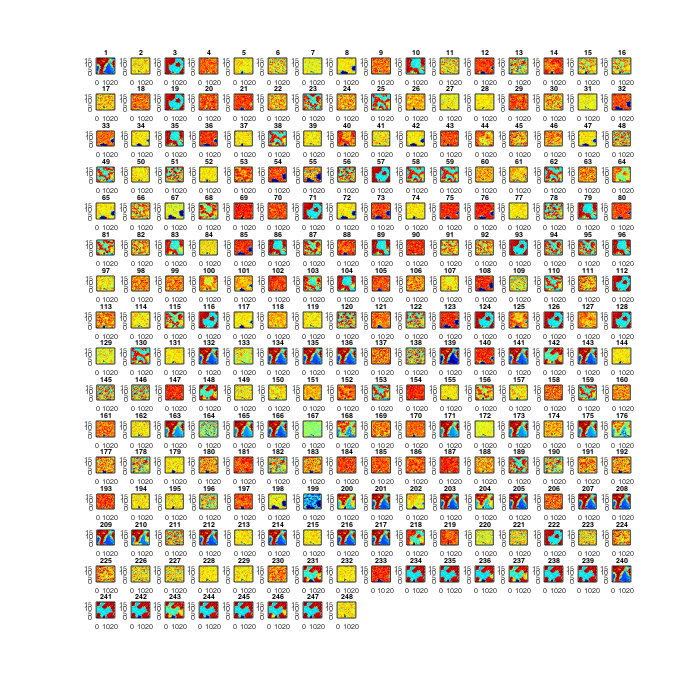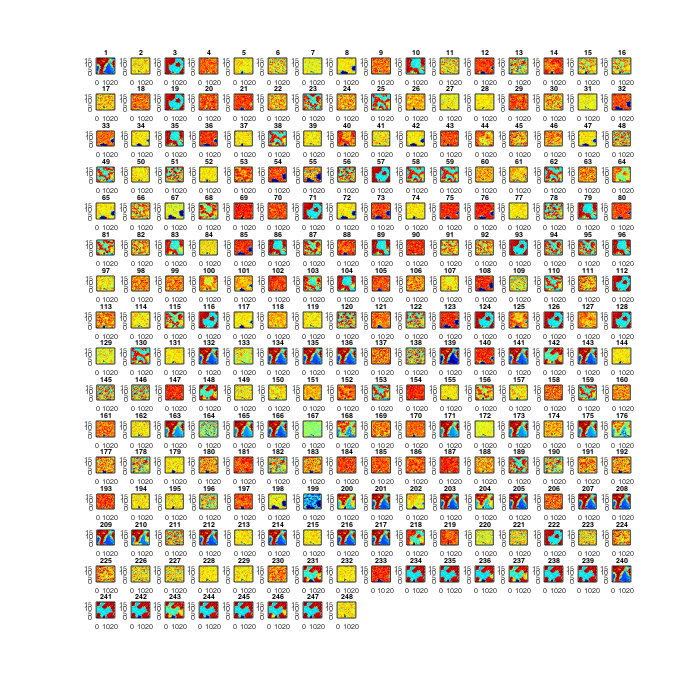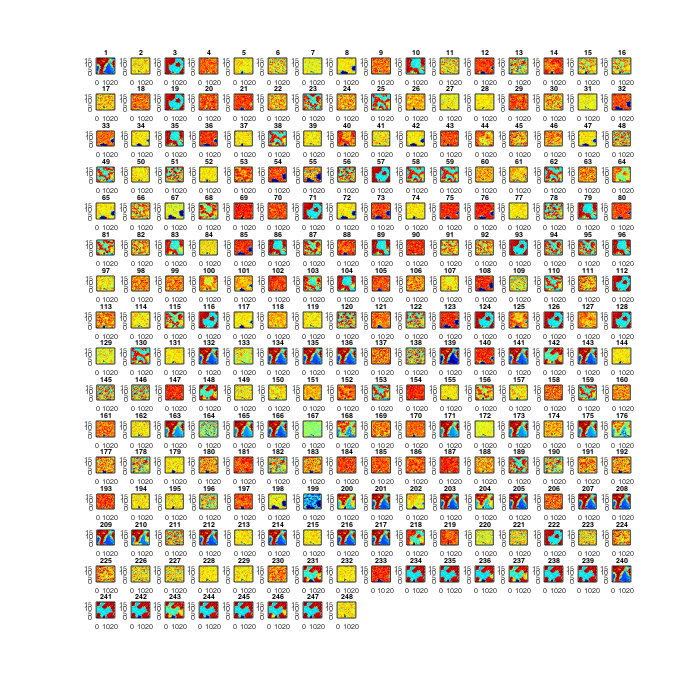  Europe:  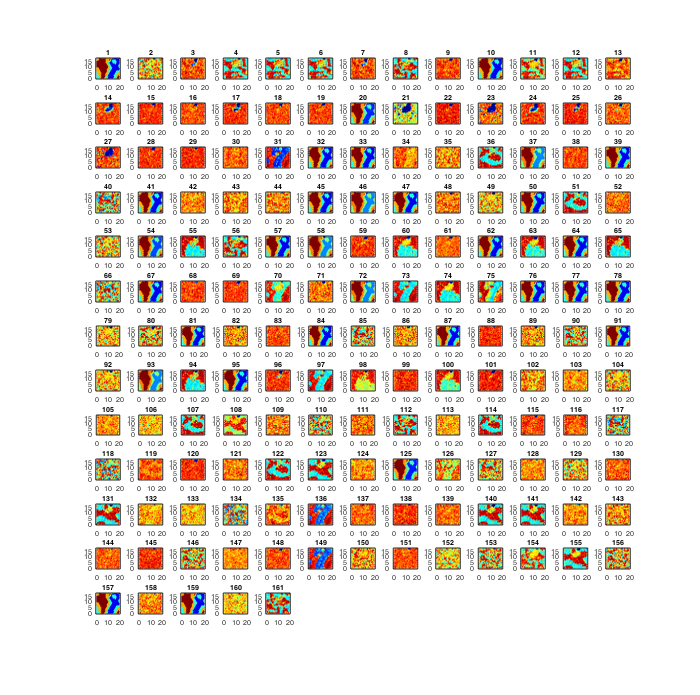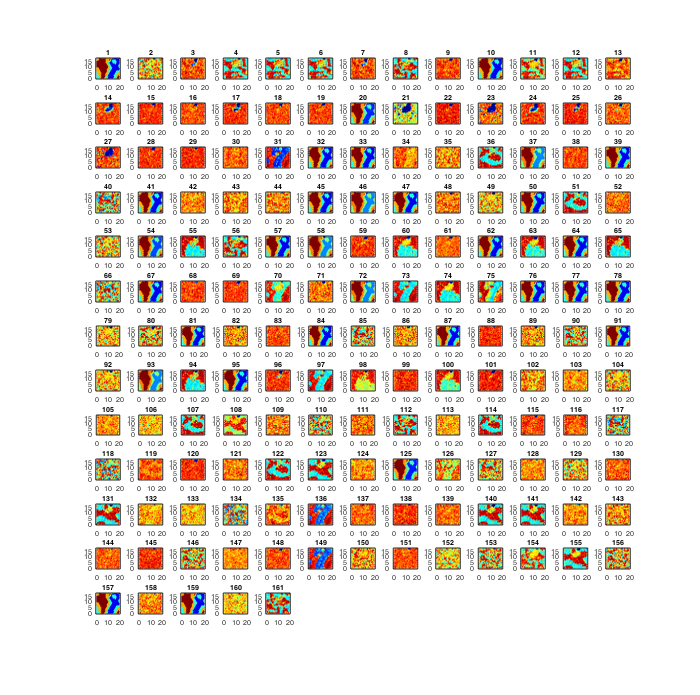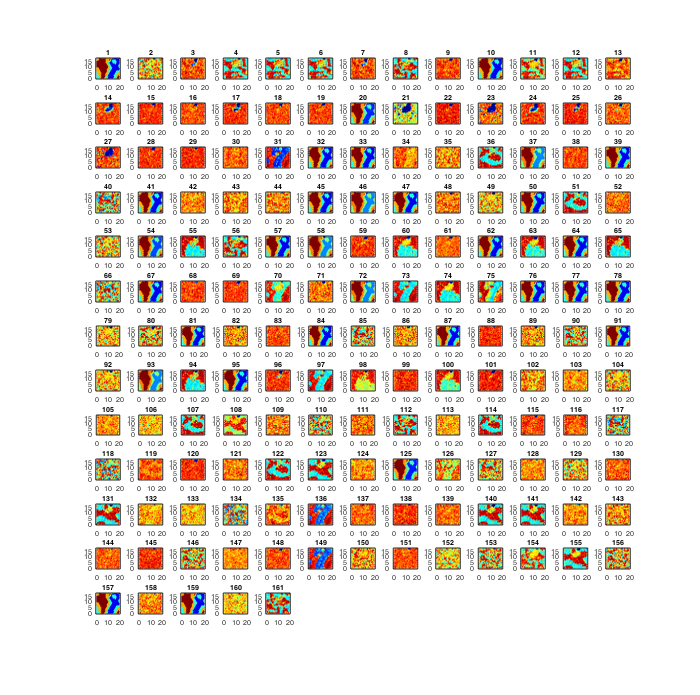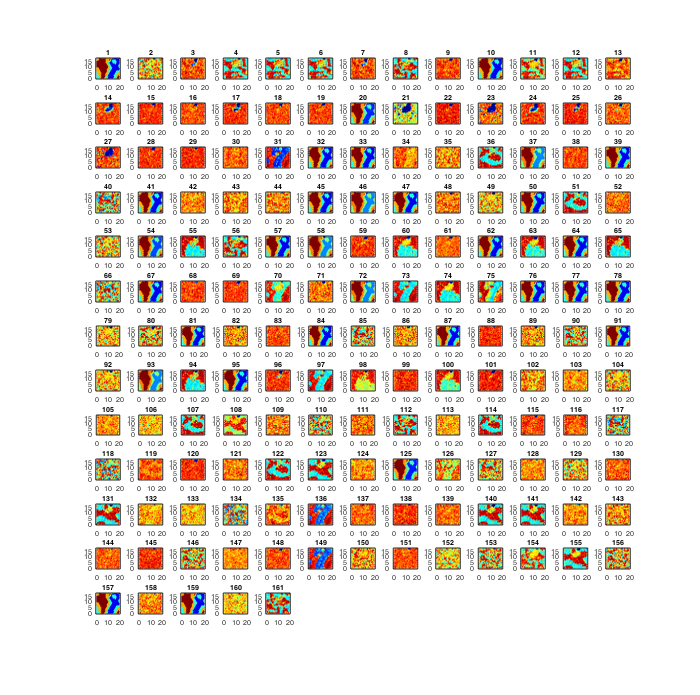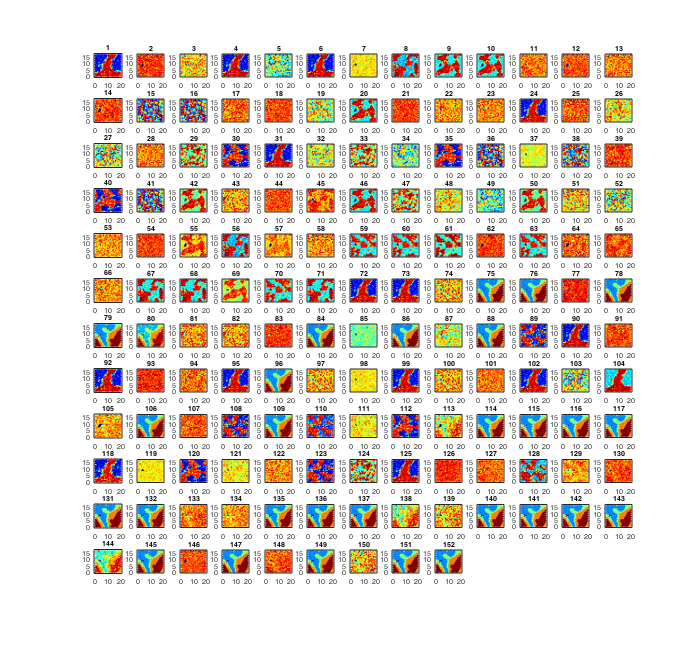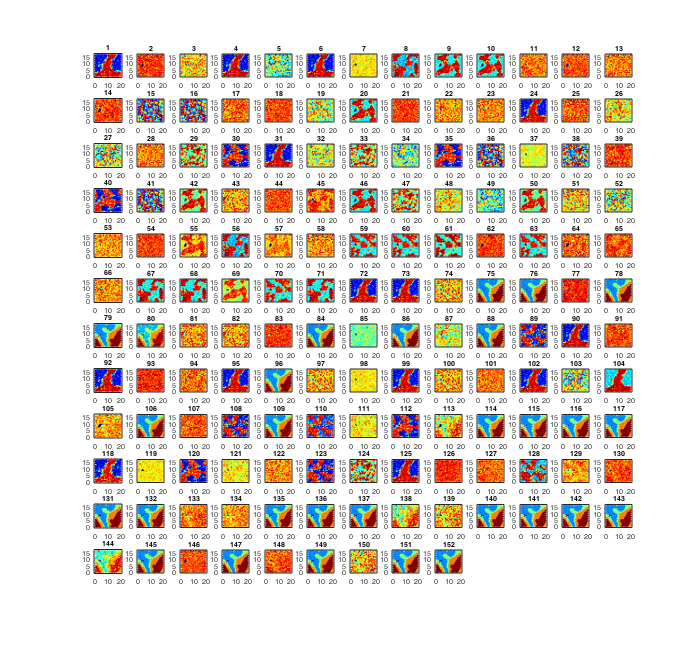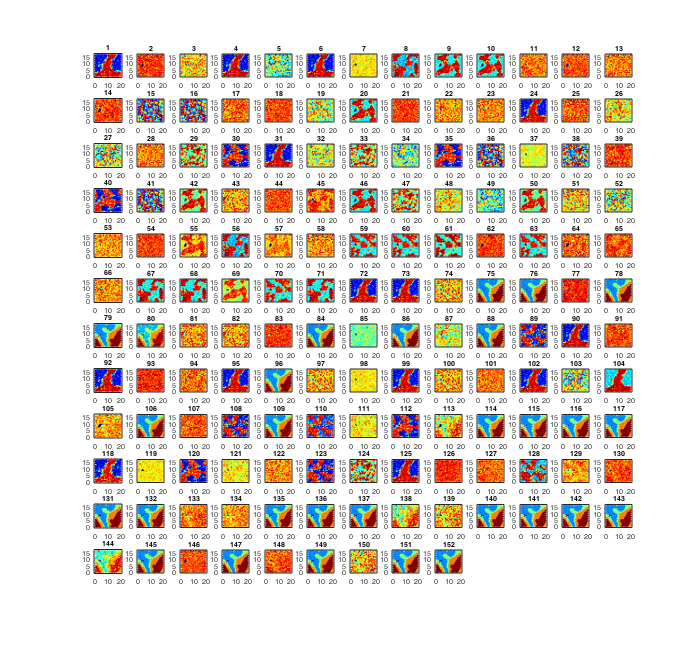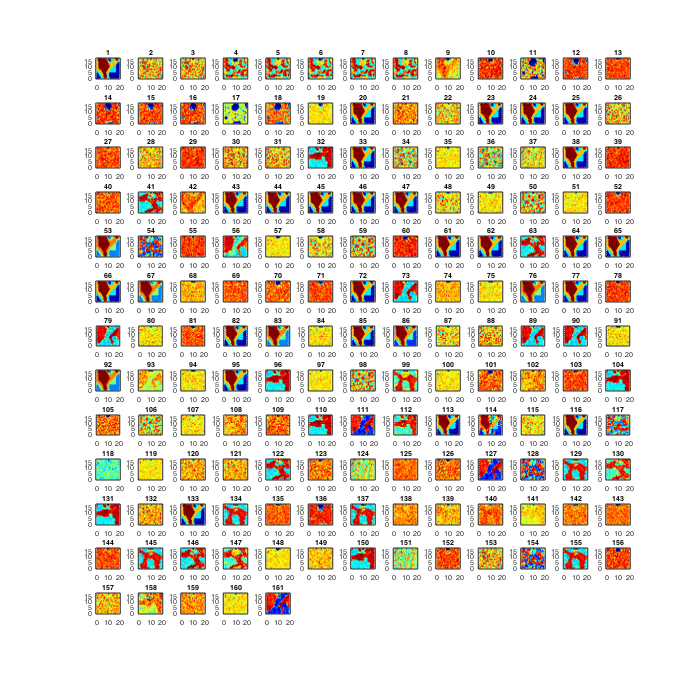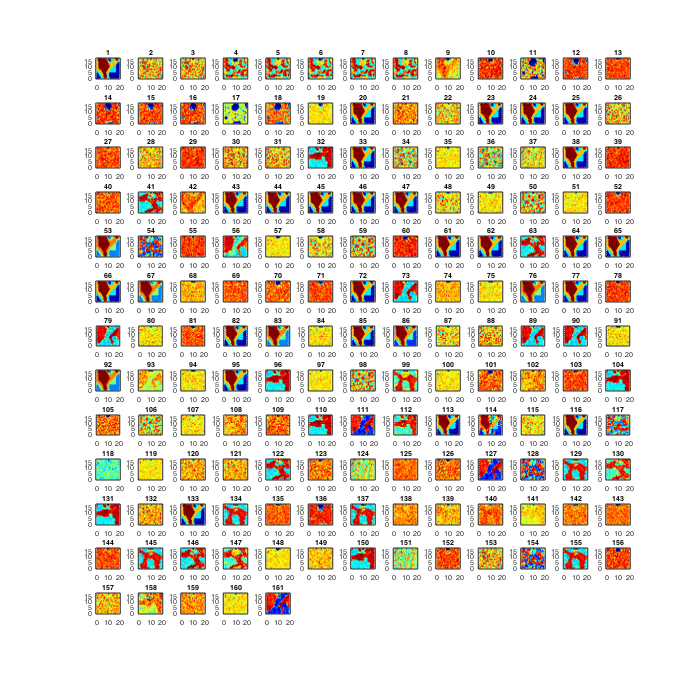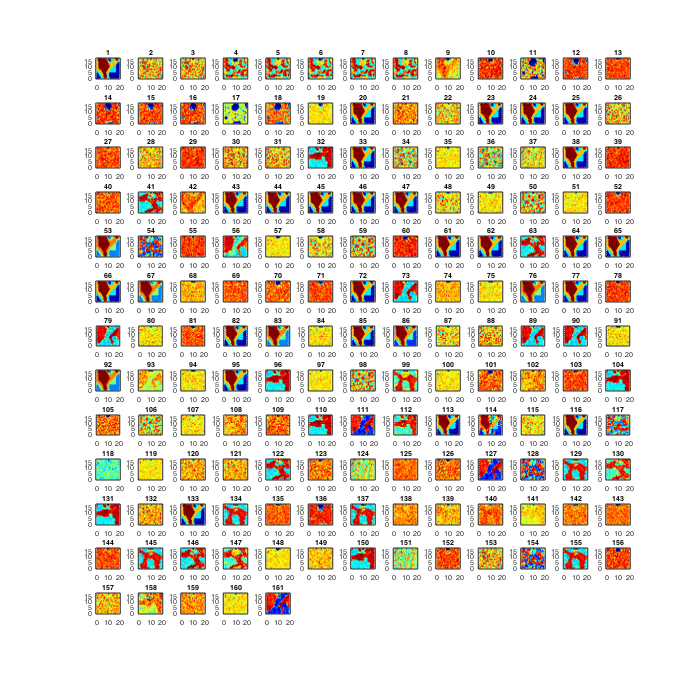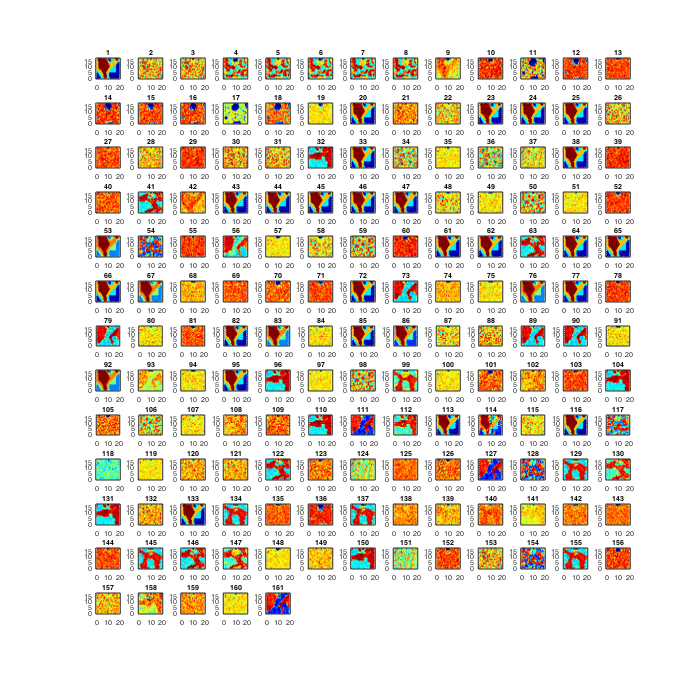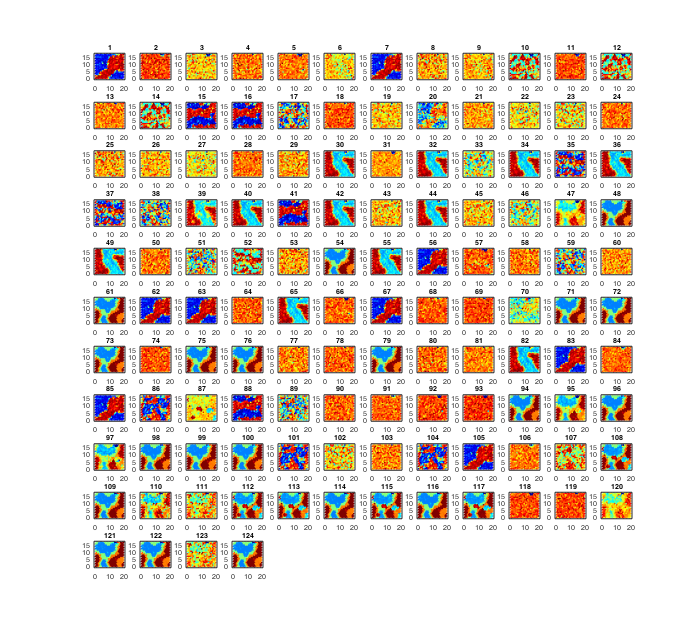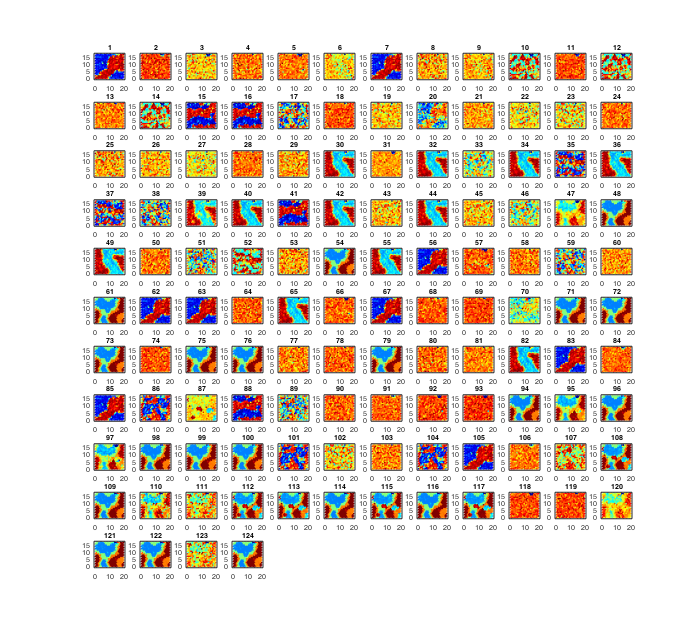  North America:  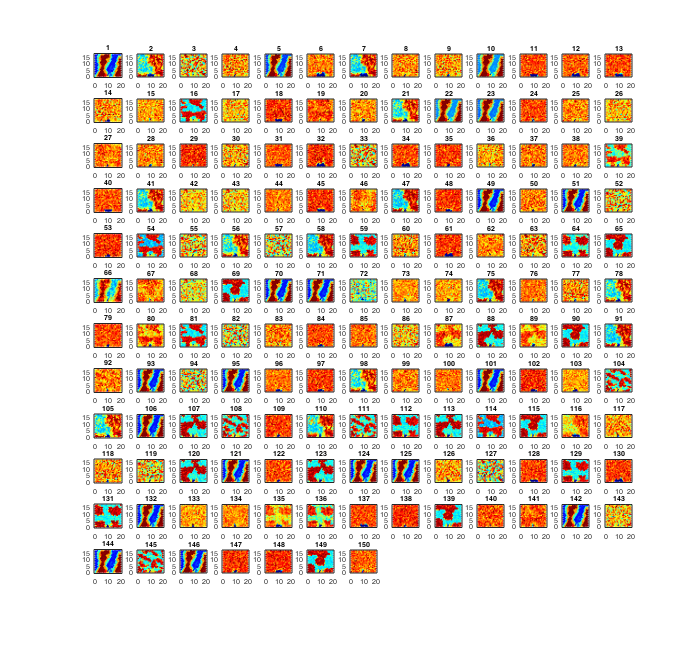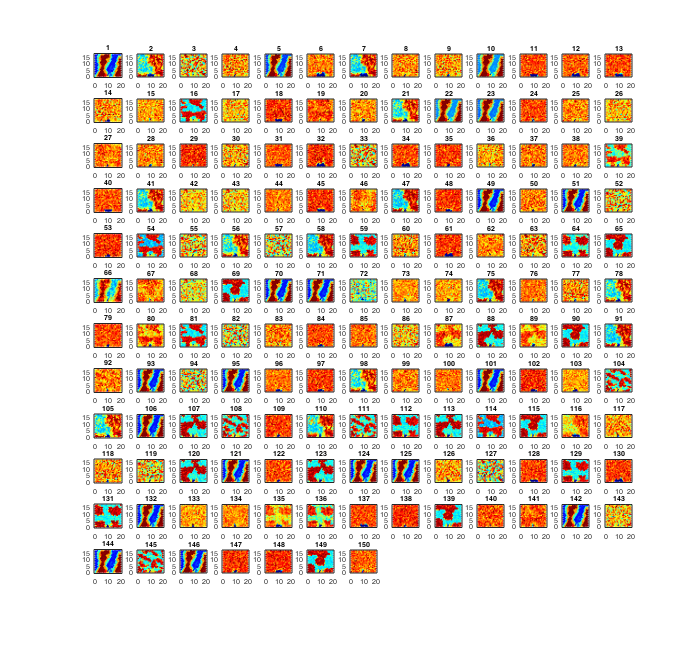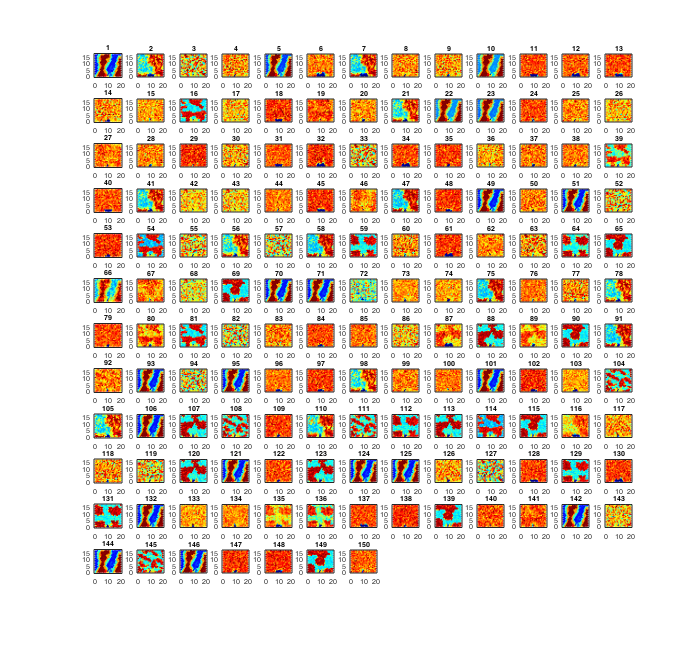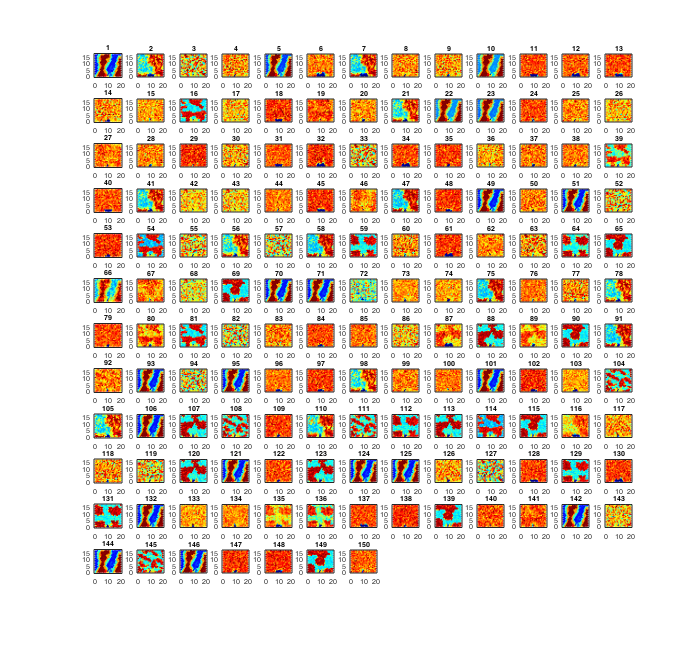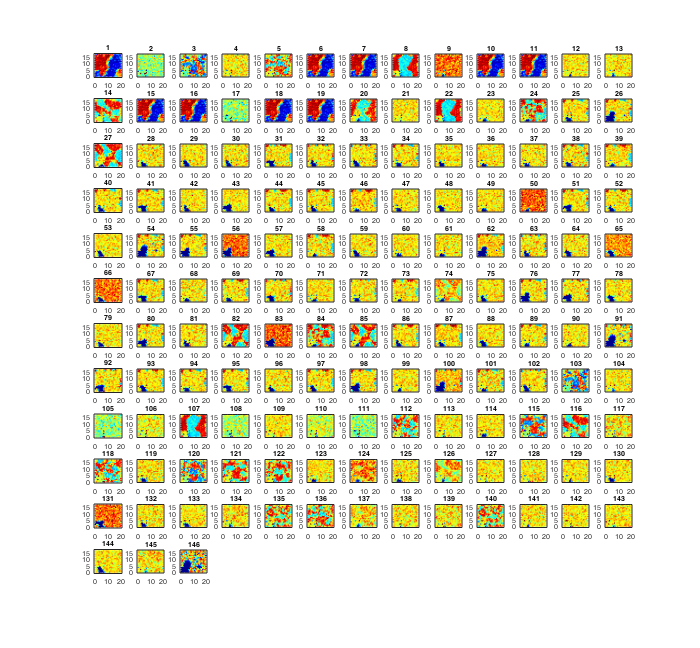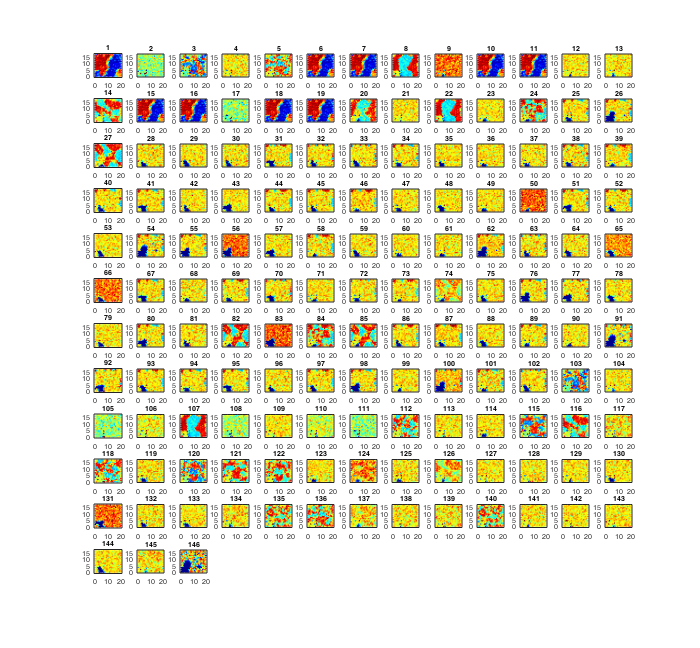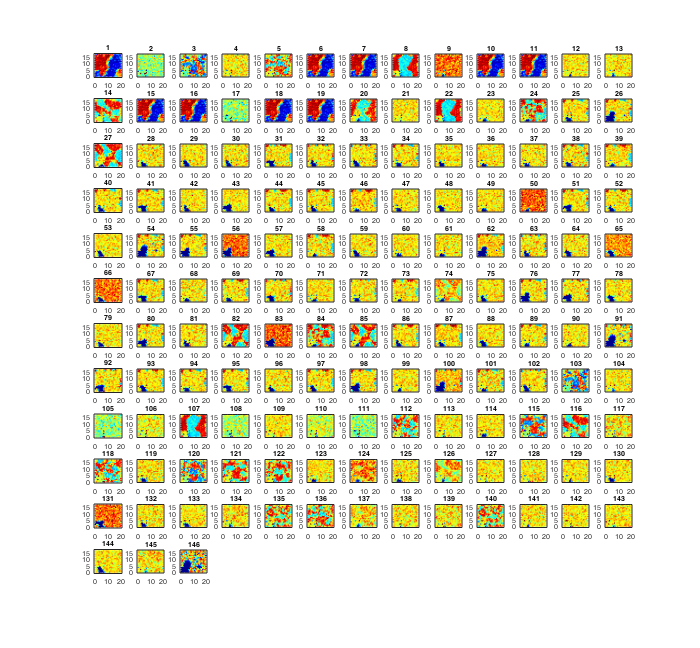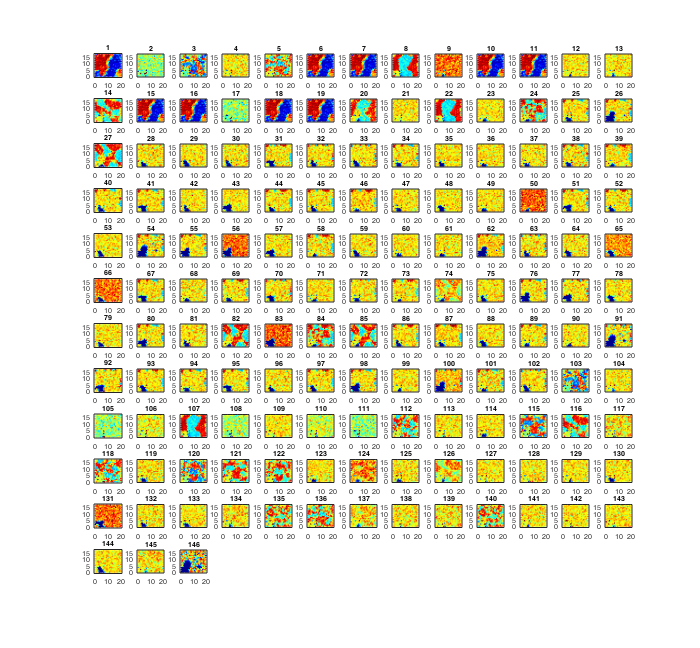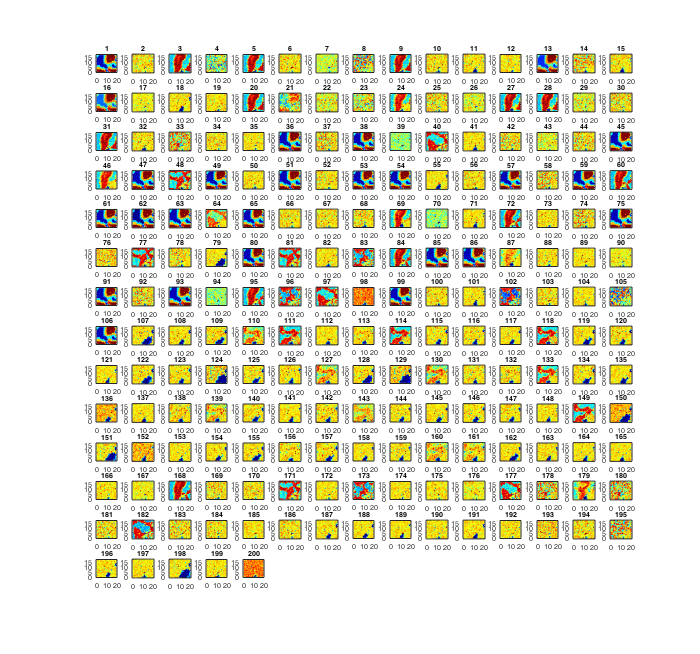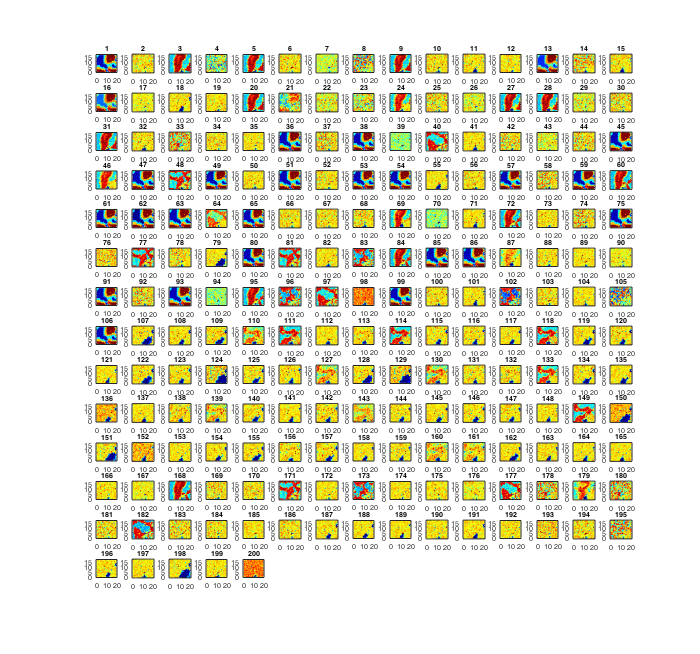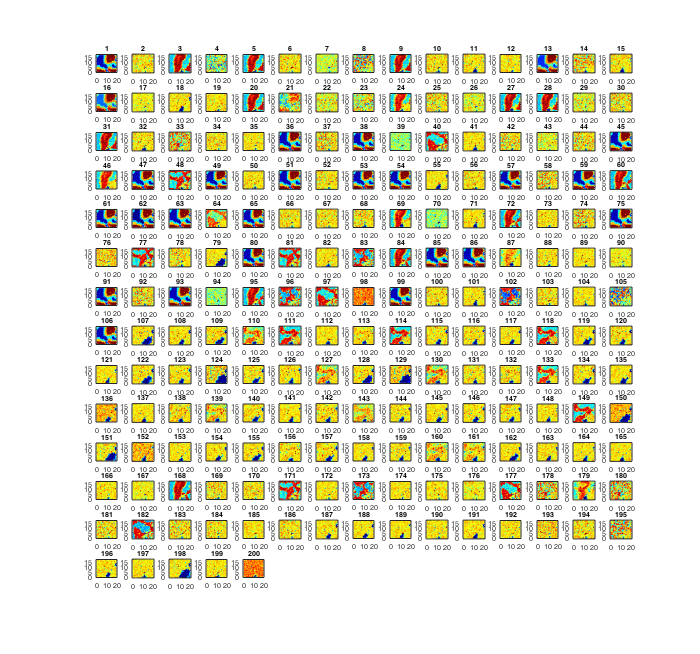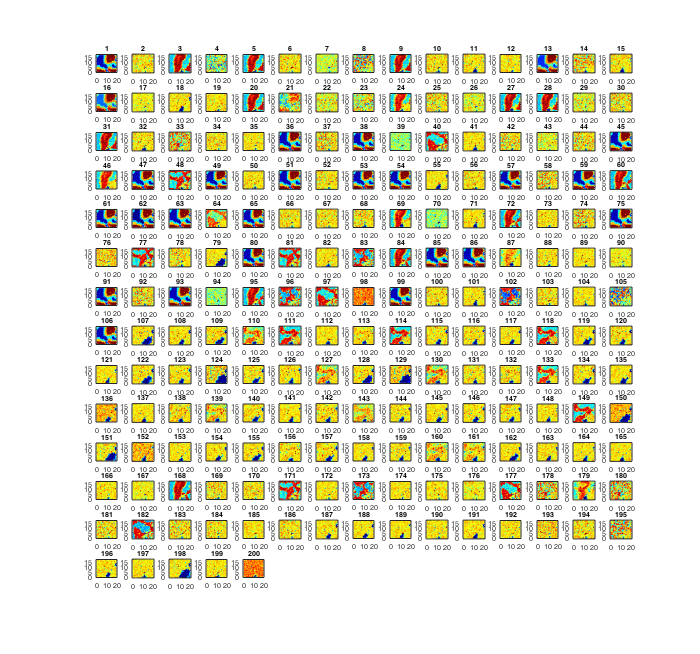  South America:  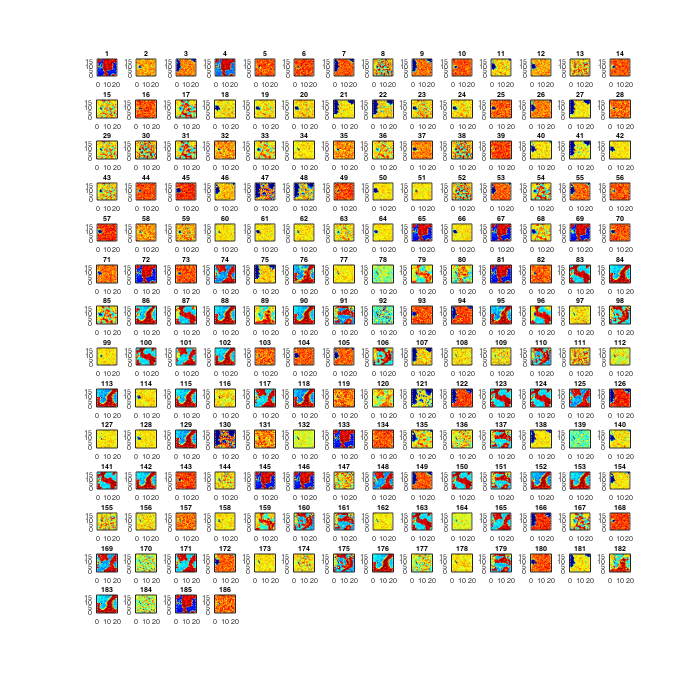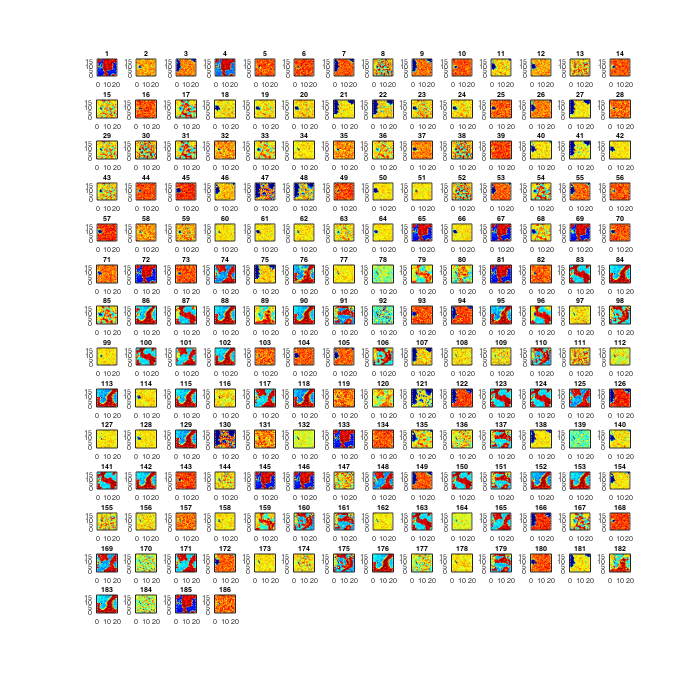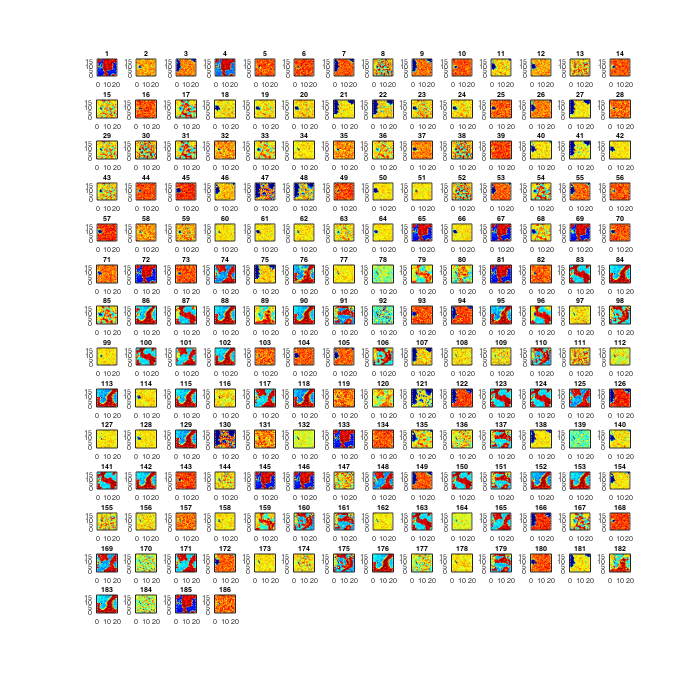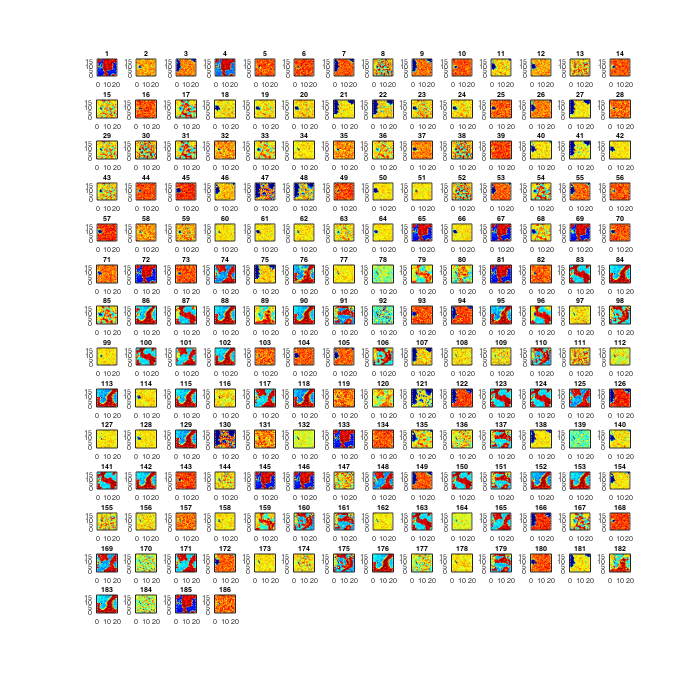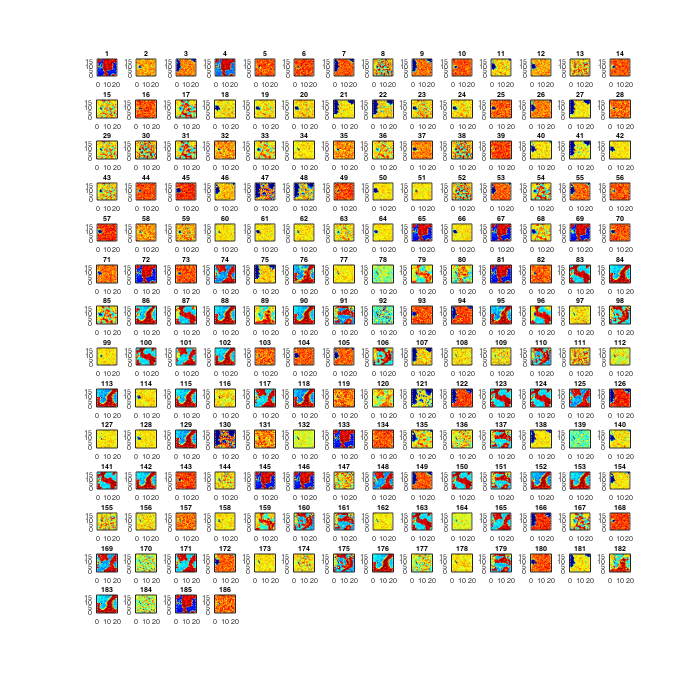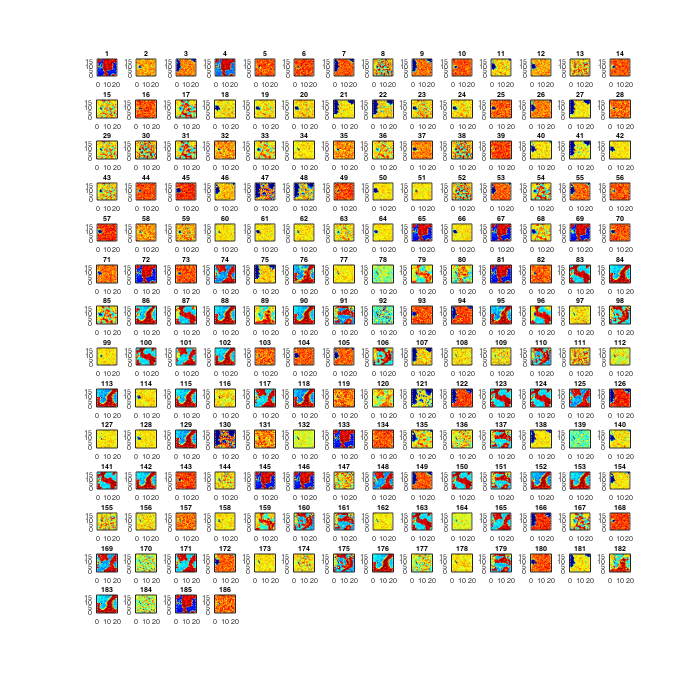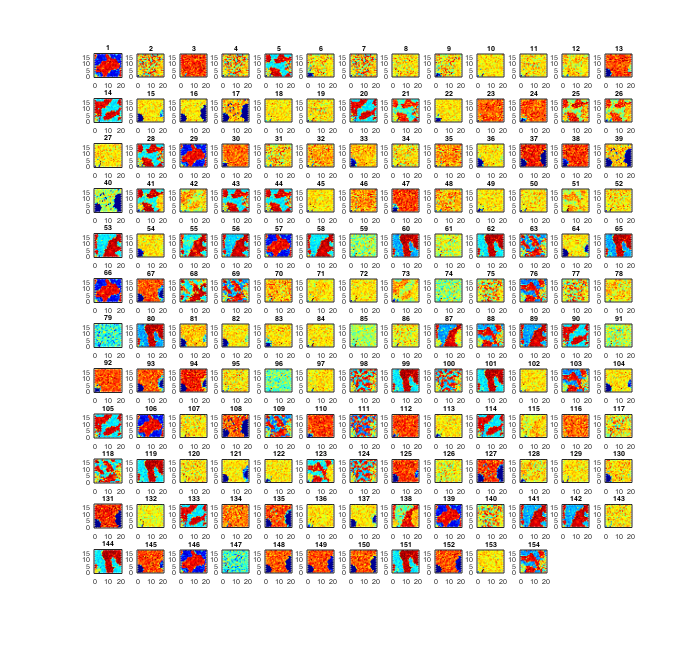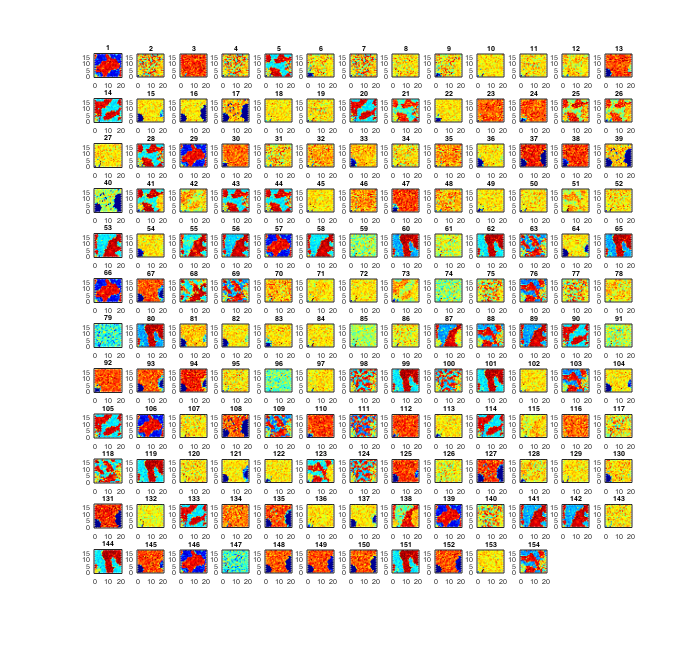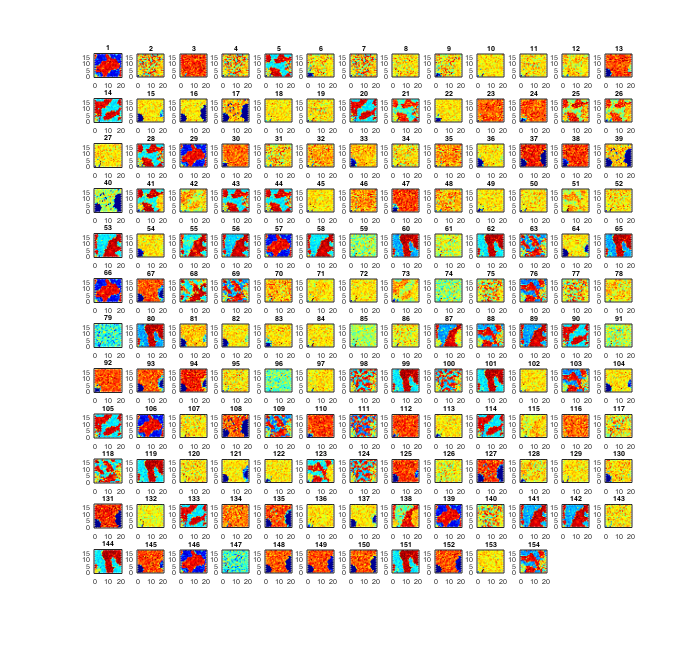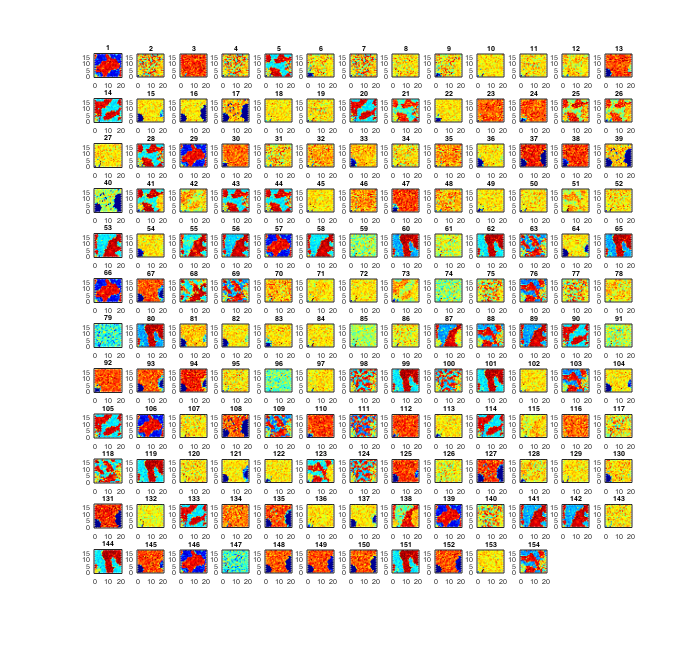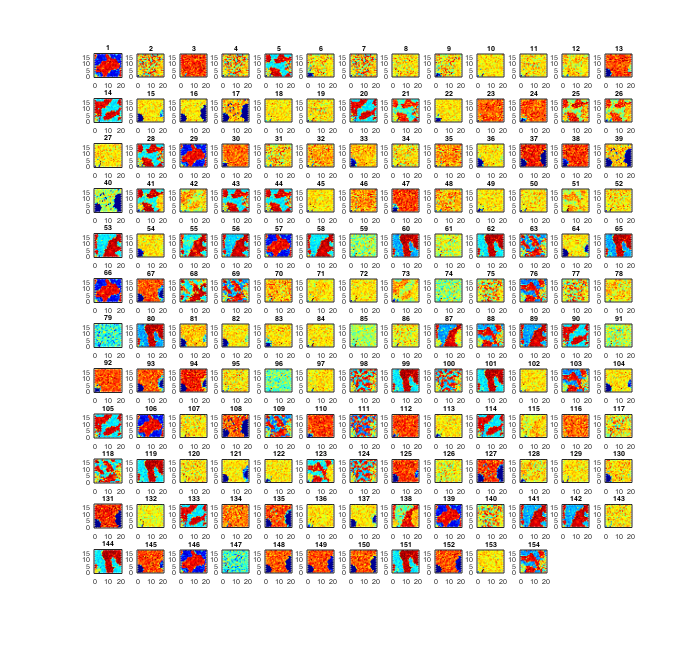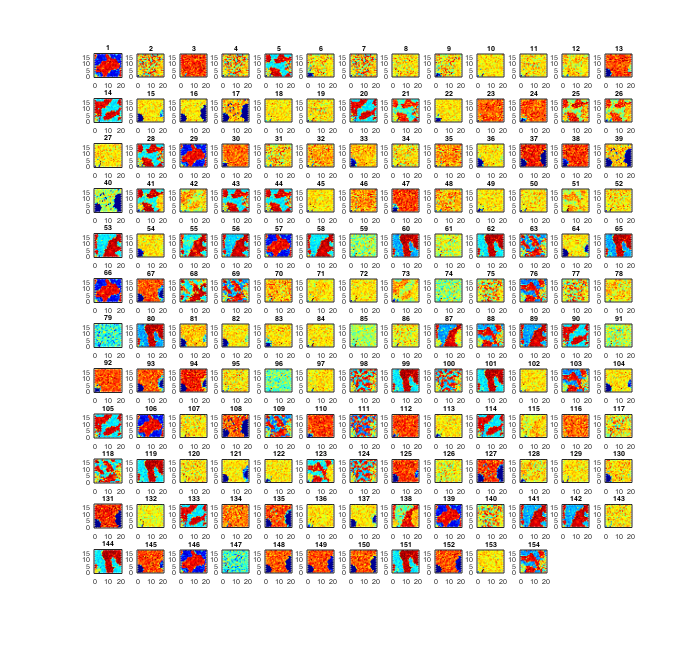  Oceania:   | Male:  CMR_1, GHA_1, GHA_6, GHA_7, GHA_9, GHA_10, GHA_11, GHA_13, ZAF_1, ZAF_2, ZAF_4, ZAF_7, ZAF_13, ZAF_17, ZAF_18, ZAF_32, ZAF_33, ZAF_35, ZAF_39, ZAF_42, ZAF_47, ZAF_55, ZAF_66, ZAF_83, ZAF_84, ZAF_89, ZAF_91, ZAF_94, ZAF_99, ZAF_100, ZAF_102, ZAF_108, ZAF_115, ZAF_116, ZAF_118, ZAF_119, ZAF_124, ZAF_129, ZAF_132, ZAF_137, ZAF_141, ZAF_148, ZAF_150, ZAF_162, ZAF_163, ZAF_166, ZAF_168, ZAF_170, ZAF_174, ZAF_177, ZAF_179, ZAF_182, ZAF_185, ZAF_186, ZAF_195, ZAF_196, ZAF_201, ZAF_203, ZAF_207, ZAF_219, ZAF_240, ZAF_251, ZAF_264, ZAF_270, ZAF_275, ZAF_277, ZAF_287, ZAF_288, ZAF_290, ZAF_295, ZAF_297, ZAF_298, ZAF_302, ZAF_303, ZAF_304, ZAF_330, ZAF_338, ZAF_345, ZAF_347, ZAF_350, ZAF_364, ZAF_368, ZAF_369, ZAF_373, ZAF_374, ZAF_375, ZAF_383, ZAF_385, ZAF_390, ZAF_403, ZAF_407, ZAF_409, ZAF_412, ZAF_426, ZAF_429, ZAF_436, ZAF_442, ZAF_449, ZAF_450, ZAF_461, ZAF_463, ZAF_485, ZAF_486, ZAF_495, ZAF_499, EGY_2, EGY_4, EGY_6, EGY_8, EGY_10, EGY_11, EGY_17, EGY_20, TUN_2, TUN_8, MAR_1, MAR_2, MOZ_2, MOZ_4, NGA_13, SEN_2, SEN_4, SEN_9, SEN_13, SEN_21, SEN_22, SEN_26, SEN_28, SEN_34, SEN_37, SEN_38, SEN_45, SEN_48, SEN_54, SEN_57, SEN_59, SEN_66, SEN_69, SEN_72, SEN_73, SEN_76, SEN_79, SEN_81, SEN_82, RWA_1, RWA_2, RWA_3, RWA_5, RWA_7, RWA_9, RWA_10, RWA_11, RWA_12, RWA_15, RWA_16, RWA_18, RWA_20, RWA_21, RWA_22, RWA_24, RWA_25, SGP_2, SGP_22, SGP_31, SGP_90, SGP_130, SGP_141, SGP_156, SGP_162, SGP_169, SGP_194, SGP_201, SGP_302, CHN_7, CHN_10, CHN_17, CHN_25, CHN_26, CHN_27, CHN_57, CHN_64, CHN_71, CHN_96, CHN_98, CHN_101, CHN_110, CHN_125, CHN_189, KWT_2, MYS_2, MYS_4, MYS_19, MYS_29, MYS_42, LKA_2, IND_2, IND_3, IND_4, IND_6, IND_9, IND_14, IND_29, IND_37, IND_44, IND_48, IND_61, IND_66, IND_68, IND_69, IND_71, IND_79, IND_82, IND_89, IND_97, IND_107, IND_108, IND_116, IND_119, IND_123, IND_135, IND_139, IND_140, IND_143, IND_150, IND_167, IND_169, IND_174, IND_175, IND_194, IND_210, IND_228, IND_248, IND_249, IND_256, IND_258, IND_259, IND_261, IND_264, IND_273, IND_277, IND_278, IND_282, IND_289, IND_314, IND_323, IND_332, IND_335, IND_369, IND_370, IND_372, IND_374, IND_396, IND_414, IND_416, IND_426, IND_430, IND_448, IND_474, IND_480, IND_484, IND_503, IND_505, IND_506, IND_520, IND_523, IND_537, IND_539, IND_547, IND_562, IND_567, IND_628, IND_632, IND_634, IND_637, IND_649, IND_663, IND_666, IND_681, IND_685, IND_697, IND_702, IND_714, IND_735, IND_768, IND_792, IND_794, IND_802, IND_808, IND_815, IND_828, IND_869, IND_891, IND_898, IND_981, IND_987, IND_996, IDN_6, IDN_13, IDN_51, IDN_53, TUR_5, TUR_14, TUR_16, TUR_35, TUR_48, TUR_53, TUR_69, VNM_2, ISR_11, OMN_20, LBN_3, ARE_26, ARE_38, ARE_59, ARE_64, ARE_66, ARE_69, FRO_2, FRO_3, FRO_4, BEL_1, POL_4, POL_5, ROU_2, ROU_9, ESP_9, ESP_16, ESP_17, ESP_25, ESP_33, ESP_35, ESP_50, ESP_57, ESP_61, ESP_65, ESP_103, ESP_116, ESP_134, ESP_141, ESP_147, GEO_2, ITA_2, ITA_3, ITA_9, ITA_12, ITA_34, ITA_35, ITA_40, ITA_41, ITA_55, ITA_70, ITA_79, ITA_84, ITA_107, ITA_108, ITA_125, ITA_126, ITA_143, ITA_164, ITA_165, ITA_169, ITA_177, ITA_179, ITA_184, ITA_206, ITA_223, ITA_238, ITA_243, ITA_245, ITA_247, ITA_255, ITA_259, ITA_266, ITA_274, ITA_285, ITA_290, ITA_291, ITA_297, ITA_299, ITA_303, RUS_2, RUS_7, RUS_10, RUS_25, RUS_28, RUS_29, RUS_30, RUS_36, RUS_42, FRA_1, FRA_2, FRA_4, FRA_7, FRA_8, FRA_10, FRA_16, FRA_19, FRA_35, FRA_36, FRA_44, FRA_47, FRA_52, FRA_56, FRA_60, FRA_63, FRA_65, FRA_71, FRA_76, SVK_1, HUN_1, HUN_5, HUN_17, HUN_25, UKR_1, UKR_4, HRV_5, CZE_1, CZE_2, CZE_9, CZE_17, CZE_18, CZE_19, CZE_24, CZE_25, CZE_31, CZE_35, CZE_38, CZE_41, CZE_49, CZE_56, CZE_69, CZE_73, CZE_82, CZE_85, MEX_1, MEX_6, MEX_18, MEX_24, MEX_53, MEX_55, USA_1, USA_13, USA_16, USA_21, USA_25, USA_35, USA_37, USA_41, USA_43, USA_60, USA_70, USA_78, USA_79, USA_92, USA_94, USA_97, USA_99, USA_100, USA_122, USA_137, USA_139, USA_141, USA_142, USA_149, USA_151, USA_152, USA_153, USA_155, USA_157, USA_159, USA_165, USA_173, USA_191, USA_199, USA_209, USA_219, USA_231, USA_255, USA_262, USA_285, USA_295, USA_312, USA_315, PAN_6, PAN_116, PAN_119, PAN_122, Saint Martin_2, GLP_5, CRI_5, CRI_6, CRI_8, CRI_12, CRI_13, CRI_16, CRI_18, COL_3, COL_11, COL_13, COL_32, COL_33, COL_34, COL_42, COL_45, COL_50, COL_56, COL_69, COL_70, COL_90, COL_103, COL_104, ECU_1, ECU_16, BRA_1, BRA_4, BRA_7, BRA_8, BRA_11, BRA_12, BRA_13, BRA_15, BRA_16, BRA_17, BRA_18, BRA_19, BRA_22, BRA_33, BRA_36, BRA_44, BRA_48, BRA_51, BRA_52, BRA_58, BRA_66, BRA_71, BRA_74, BRA_76, BRA_79, BRA_83, BRA_88, BRA_92, BRA_94, BRA_99, BRA_105, BRA_107, BRA_122, BRA_127, BRA_134, BRA_145, BRA_147, BRA_156, BRA_158, BRA_160, BRA_163, BRA_187, BRA_190, BRA_193, BRA_202, BRA_216, BRA_218, BRA_227, BRA_229, BRA_236, BRA_247, BRA_250, AUS_1, AUS_2, AUS_3, AUS_4, AUS_6, AUS_7, PAN_139, IDN_47, ROU_5, ESP_74, USA_8, PAN_100, CAN_14, COL_76, BRA_188  Female:  GHA_1, ZAF_2, ZAF_10, ZAF_11, ZAF_14, ZAF_17, ZAF_19, ZAF_22, ZAF_23, ZAF_24, ZAF_34, ZAF_35, ZAF_36, ZAF_47, ZAF_50, ZAF_53, ZAF_57, ZAF_59, ZAF_66, ZAF_69, ZAF_70, ZAF_75, ZAF_80, ZAF_82, ZAF_84, ZAF_89, ZAF_90, ZAF_110, ZAF_112, ZAF_121, ZAF_127, ZAF_132, ZAF_133, ZAF_147, ZAF_149, ZAF_150, ZAF_152, ZAF_153, ZAF_160, ZAF_164, ZAF_165, ZAF_169, ZAF_172, ZAF_179, ZAF_185, ZAF_189, ZAF_193, ZAF_194, ZAF_196, ZAF_207, ZAF_217, ZAF_226, ZAF_238, ZAF_240, ZAF_244, ZAF_245, ZAF_263, ZAF_265, ZAF_266, ZAF_267, ZAF_270, ZAF_293, ZAF_302, ZAF_313, ZAF_314, ZAF_319, ZAF_321, ZAF_324, ZAF_327, ZAF_329, ZAF_341, ZAF_343, ZAF_345, ZAF_349, ZAF_357, ZAF_363, ZAF_373, ZAF_374, ZAF_381, ZAF_383, ZAF_388, ZAF_396, ZAF_397, ZAF_400, ZAF_405, ZAF_408, ZAF_429, ZAF_442, ZAF_450, ZAF_451, ZAF_453, ZAF_458, ZAF_469, ZAF_470, ZAF_472, ZAF_493, ZAF_497, ZAF_499, ZAF_510, ZAF_525, ZAF_528, ZAF_532, ZAF_552, ZAF_553, ZAF_558, ZAF_562, ZAF_572, ZAF_574, ZAF_583, ZAF_585, ZAF_603, ZAF_620, ZAF_623, ZAF_632, ZAF_634, ZAF_649, ZAF_651, ZAF_660, ZAF_662, ZAF_666, ZAF_669, ZAF_683, ZAF_693, ZAF_701, ZAF_706, ZAF_708, ZAF_722, ZAF_727, ZAF_751, ZAF_754, ZAF_755, ZAF_764, ZAF_767, ZAF_771, ZAF_773, ZAF_778, ZAF_791, ZAF_797, ZAF_801, ZAF_803, ZAF_817, ZAF_833, ZAF_849, ZAF_853, ZAF_872, ZAF_873, ZAF_884, ZAF_889, ZAF_894, ZAF_897, ZAF_907, ZAF_913, ZAF_915, ZAF_926, ZAF_931, ZAF_934, ZAF_935, ZAF_953, ZAF_960, ZAF_961, ZAF_988, ZAF_992, ZAF_997, ZAF_1003, TUN_3, TUN_9, SEN_3, SEN_4, SEN_8, SEN_12, SEN_16, SEN_18, SEN_19, SEN_26, SEN_39, SEN_41, CHN_17, CHN_32, CHN_36, CHN_42, CHN_73, CHN_94, CHN_121, MYS_13, MYS_19, MYS_26, LKA_6, BGD_1, BGD_2, BGD_5, IND_4, IND_5, IND_6, IND_8, IND_13, IND_14, IND_15, IND_16, IND_19, IND_22, IND_23, IND_24, IND_25, IND_28, IND_34, IND_39, IND_41, IND_44, IND_53, IND_54, IND_55, IND_58, IND_60, IND_61, IND_64, IND_65, IND_75, IND_76, IND_77, IND_89, IND_91, IND_94, IND_98, IND_99, IND_110, IND_112, IND_115, IND_117, IND_118, IND_119, IND_120, IND_122, IND_123, IND_128, IND_130, IND_137, IND_139, IND_150, IND_160, IND_162, IND_168, IND_175, IND_176, IND_179, IND_181, IND_189, IND_191, IND_196, IND_199, IND_215, IND_216, IND_219, IND_221, IND_223, IND_225, IND_236, IND_244, IND_266, IND_281, IND_304, IND_310, IND_317, IND_318, IND_319, IND_322, IND_330, IND_348, IND_352, IND_365, IND_368, IND_386, IND_389, IND_392, IND_393, IND_396, IND_398, IND_408, IND_421, IND_424, IND_437, IND_444, IND_447, IND_465, IND_469, IND_472, IND_482, IND_487, IND_497, IND_499, IND_501, IND_502, IND_504, IND_510, IND_512, IND_514, IND_515, IND_522, IND_530, IND_541, IND_548, IND_551, IND_552, IND_554, IND_555, South Korea_2, IDN_10, IDN_23, TUR_7, TUR_28, TUR_41, TUR_53, Iran_4, TWN_2, TWN_10, TWN_24, VNM_1, VNM_5, VNM_8, VNM_16, VNM_17, PAK_1, OMN_6, OMN_14, OMN_19, OMN_20, OMN_21, OMN_23, OMN_28, LBN_4, ARE_5, ARE_6, ARE_8, ARE_11, ARE_14, ARE_15, ARE_18, ARE_20, ARE_21, ARE_23, ARE_25, ARE_36, ARE_38, FRO_2, FRO_3, FRO_7, BEL_1, BEL_3, GRC_1, DEU_3, ROU_2, ROU_4, ROU_6, ROU_7, ROU_23, ROU_24, ESP_5, ESP_16, ESP_17, ESP_31, ESP_57, ESP_67, ESP_88, ESP_90, ESP_93, GEO_1, ITA_4, ITA_10, ITA_12, ITA_35, ITA_37, ITA_38, ITA_50, ITA_58, ITA_60, ITA_64, ITA_74, ITA_77, ITA_79, ITA_81, ITA_85, ITA_86, ITA_90, ITA_98, ITA_104, ITA_121, ITA_123, ITA_126, ITA_130, ITA_139, ITA_142, ITA_155, ITA_166, ITA_195, ITA_204, ITA_215, ITA_217, ITA_218, ITA_232, RUS_2, RUS_6, RUS_8, RUS_15, RUS_18, RUS_23, RUS_25, RUS_28, RUS_33, RUS_48, RUS_49, RUS_59, RUS_74, RUS_75, RUS_81, RUS_82, FRA_6, FRA_8, FRA_13, FRA_25, FRA_26, FRA_27, FRA_34, FRA_35, FRA_40, FRA_44, SVK_1, SVK_2, HUN_3, HUN_7, HUN_10, HUN_18, HUN_27, HUN_41, HUN_43, BIH_1, CZE_1, CZE_10, CZE_11, CZE_24, CZE_27, CZE_28, CZE_35, CZE_38, CZE_45, CZE_50, CZE_66, CZE_69, CZE_71, CZE_82, CZE_85, MEX_2, MEX_3, MEX_8, MEX_21, MEX_22, MEX_30, MEX_33, MEX_35, MEX_36, MEX_40, USA_8, USA_15, USA_18, USA_41, USA_56, USA_72, USA_86, USA_101, USA_120, USA_126, USA_130, USA_134, USA_139, USA_154, USA_158, USA_167, USA_174, PAN_4, PAN_8, PAN_16, PAN_33, PAN_37, PAN_43, PAN_57, PAN_63, PAN_76, PAN_90, PAN_91, Saint Martin_3, GLP_4, GLP_6, CAN_1, CAN_5, CAN_12, COL_2, COL_18, COL_27, COL_28, COL_34, COL_35, COL_39, COL_66, COL_67, COL_68, COL_69, ECU_1, BRA_2, BRA_4, BRA_6, BRA_8, BRA_10, BRA_13, BRA_16, BRA_18, BRA_20, BRA_30, BRA_31, BRA_40, BRA_45, BRA_52, BRA_67, BRA_68, BRA_69, BRA_73, BRA_84, BRA_87, BRA_92, BRA_101, BRA_113, BRA_136, BRA_145, BRA_149, BRA_150, BRA_161, BRA_193, BRA_194, BRA_213, BRA_230, BRA_231, BRA_237, BRA_257, BRA_260, AUS_1, AUS_2, AUS_3, AUS_4, AUS_5, ESP_23, IDN_21, PAN_5 |

Key: An isolate is defined by the Alpha-3 country code_unique isolate number. Where country code is not available, the full country name is adopted. For this study, the following countries were harvested. ARE-United Arab Emirates, ARG-Argentina, AUS-Australia, AUT-Austria, BEL-Belgium, BGD-Bangladesh, BHR-Bahrain, BIH-Bosnia and Herzegovina, BRA-Brazil, CAN-Canada, CHE-Switzerland, CHL-Chile, CHN-China, CMR-Cameroon, COL-Colombia, CRI-Costa Rica, CYP-Cyprus, CZE-Czechia, DEU-Germany, DOM-Dominican Republic, DZA-Algeria, ECU-Ecuador, EGY-Egypt, ESP-Spain, FIN-Finland, FRA-France, FRO-Faroe Islands, GEO-Georgia, GHA-Ghana, GLP-Guadeloupe, GMB-Gambia, GRC-Greece, GUM-Guam, HRV-Croatia, HUN-Hungary, IDN-Indonesia, IND-India, IRQ-Iraq, ISR-Israel, ITA-Italy, Iran-Iran, KAZ-Kazakhstan, KWT-Kuwait, LBN-Lebanon, LKA-Sri Lanka, MAR-Morocco, MDA-Republic of Moldova, MDG-Madagascar, MEX-Mexico, MNG-Mongolia, MOZ-Mozambique, MYS-Malaysia, NGA-Nigeria, NZL-New Zealand, OMN-Oman, PAK-Pakistan, PAN-Panama, PER-Peru, PHL-Philippines, POL-Poland, ROU-Romania, RUS-Russian Federation, RWA-Rwanda, SAU-Saudi Arabia, SEN-Senegal, SGP-Singapore, SVK-Slovakia, SWE-Sweden, Saint Martin-Saint Martin, South Korea-South Korea, TUN-Tunisia, TUR-Turkey, TWN-Taiwan Province of China, UKR-Ukraine, USA-United States of America, VEN-Bolivarian Republic of Venezuela, VNM-Viet Nam.
